# Effectiveness of Immune Checkpoint Inhibitors in Patients With Advanced Esophageal Squamous Cell Carcinoma: A Meta-analysis Including Low PD-L1 Subgroups

**DOI:** 10.1001/jamaoncol.2022.5816

**Published:** 2022-12-08

**Authors:** Dominic Wei Ting Yap, Alberto Giovanni Leone, Nicky Zhun Hong Wong, Joseph J. Zhao, Jeremy Chee Seong Tey, Raghav Sundar, Filippo Pietrantonio

**Affiliations:** 1 Yong Loo Lin School of Medicine, National University of Singapore, Singapore; 2 Medical Oncology Department, Fondazione IRCCS Istituto Nazionale dei Tumori, Milan, Italy; 3 Department of Radiation Oncology, National University Cancer Institute, National University Hospital, Singapore; 4 Department of Haematology-Oncology, National University Cancer Institute, National University Hospital, Singapore; 5 Cancer and Stem Cell Biology Program, Duke-NUS Medical School, Singapore; 6 The N.1 Institute for Health, National University of Singapore, Singapore; 7 Singapore Gastric Cancer Consortium, Singapore

## Abstract

**Importance:**

Immune checkpoint inhibitors (ICIs) have improved survival outcomes of patients with advanced esophageal squamous cell carcinoma in both first- and second-line settings. However, the benefit of ICIs in patients with low programmed death ligand 1 (PD-L1) expression remains unclear.

**Objective:**

To derive survival data for patient subgroups with low PD-L1 expression from clinical trials comparing ICIs with chemotherapy in esophageal squamous cell carcinoma and to perform a pooled analysis.

**Data Sources:**

Kaplan-Meier curves from the randomized clinical trials were extracted after a systematic search of Scopus, Embase, PubMed, and Web of Science from inception until October 1, 2021.

**Study Selection:**

Randomized clinical trials that investigated the effectiveness of anti–PD-1–based regimens for advanced esophageal squamous cell carcinoma and that reported overall survival (OS), progression-free survival, or duration of response were included in this meta-analysis.

**Data Extraction and Synthesis:**

Kaplan-Meier curves of all-comer populations, subgroups with high PD-L1, and those with low PD-L1 (when available) were extracted from published articles. A graphic reconstructive algorithm was used to calculate time-to-event outcomes from these curves. In studies with unreported curves for subgroups with low PD-L1 expression, KMSubtraction was used to impute survival data. KMSubtraction is a workflow to derive unreported subgroup survival data with from subgroups. An individual patient data pooled analysis including previously reported and newly imputed subgroups was conducted for trials with the same treatment line and PD-L1 scoring system. Data analysis was conducted from January 1, 2022, to June 30, 2022.

**Main Outcomes and Measures:**

Primary outcomes included Kaplan-Meier curves and hazard ratios (HRs) for OS for subgroups with low PD-L1 expression. Secondary outcomes included progression-free survival and duration of response.

**Results:**

The randomized clinical trials CheckMate-648, ESCORT-1st, KEYNOTE-590, ORIENT-15, KEYNOTE-181, ESCORT, RATIONALE-302, ATTRACTION-3, and ORIENT-2 were included, totaling 4752 patients. In the pooled analysis of first-line trials that evaluated a tumor proportion score (CheckMate-648 and ESCORT-1st), no significant benefit in OS was observed with immunochemotherapy compared with chemotherapy in the subgroup of patients who had a tumor proportion score lower than 1% (HR, 0.91; 95% CI, 0.74-1.12; *P* = .38) compared with chemotherapy. In the pooled analysis of first-line trials that evaluated combined positive score (KEYNOTE-590 and ORIENT-15), there was a significant but modest OS benefit for immunochemotherapy compared with chemotherapy in the subgroup with a combined positive score lower than 10 (HR, 0.77; 95% CI, 0.62-0.94; *P* = .01).

**Conclusions and Relevance:**

Findings suggest a lack of survival benefit of ICI-based regimens in the first-line setting compared with chemotherapy alone in the subgroup with a tumor proportion score lower than 1%.

## Key Points

**Question:**

Does immunotherapy confer a survival benefit for patients with advanced esophageal squamous cell carcinoma and low expression of programmed death ligand 1?

**Findings:**

In this meta-analysis of 4752 patients with esophageal squamous cell carcinoma, immune checkpoint inhibitors were investigated in 9 first- and second-line trials. In the pooled analysis of first-line trials that used the tumor proportion score, no significant benefit in overall survival was observed with immunochemotherapy vs chemotherapy for the subgroup with a score lower than 1%; in the pooled analysis of first-line trials using the combined positive score, overall survival benefit with immunochemotherapy vs chemotherapy was modest but significant in the subgroup with a score lower than 10.

**Meaning:**

Findings suggest that novel strategies should be investigated in the subgroup of patients with a tumor proportion score lower than 1% as there is a lack of overall survival benefit of immune checkpoint inhibitor–based regimens in the first-line setting vs chemotherapy alone.

## Teaser

This meta-analysis examines whether immunotherapy confers a survival benefit for patients with advanced esophageal squamous cell carcinoma and low expression of PD-L1.

## Introduction

Immune checkpoint inhibitors (ICIs) have significantly changed the treatment landscape for patients with advanced esophageal squamous cell carcinoma (ESCC). Several randomized clinical trials (RCTs)^1,2,3,4^ demonstrated a significant benefit in overall survival (OS) with immunochemotherapy compared with doublet chemotherapy in the first-line treatment of metastatic ESCC. Other RCTs^5,6,7,8,9^ showed a significant OS advantage with anti–programmed death 1 (PD-1) monotherapy compared with single-agent chemotherapy in the second-line setting.

The results of the KEYNOTE-590 trial led to the approval of pembrolizumab in combination with chemotherapy for the first-line treatment of advanced ESCC. While the European Medicines Agency (EMA) authorized the use of pembrolizumab only for patients whose tumors expressed programmed death ligand 1 (PD-L1) with a combined positive score (CPS) of 10 or higher, the US Food and Drug Administration (FDA) approved pembrolizumab regardless of PD-L1 expression status.^10,11^ More recently, based on the results of the CheckMate-648 trial, first-line nivolumab plus chemotherapy was approved by the EMA for patients with a tumor proportion score (TPS) of 1% or higher, whereas the FDA approved both ipilimumab-nivolumab dual immunotherapy and nivolumab plus chemotherapy independent from PD-L1 status.^12,13^ Furthermore, single-agent pembrolizumab in the second-line setting is approved only by the FDA for patients with a CPS of 10 or higher, whereas nivolumab is approved by both the FDA and the EMA irrespective of PD-L1 status.^14,15^

The heterogeneity of these regulatory approvals derived from the evidence suggests that the use of immunotherapy per se or even combination regimens of immunochemotherapy may not be optimal for all patients. The survival benefit observed for patients with advanced solid cancers treated with combination therapies may not be superior to that expected from independent drug action, showing no synergistic effect between ICIs and chemotherapy.^16,17^ The effectiveness of ICIs for patients with low PD-L1 expression is unclear, and a relevant subset of these patients does not derive any benefit from immunotherapy.^18^ These findings highlight the importance of achieving greater precision in patient selection and developing better predictors of ICI response. The most validated predictive biomarker for sensitivity to ICIs is PD-L1 expression measured by the CPS or TPS. Most RCTs that have investigated the effectiveness of first-line anti–PD-1–based regimens in advanced ESCC focused mainly on the overall randomly assigned sample and PD-L1–positive (CPS ≥10 or TPS ≥1%) subgroup, without reporting the Kaplan-Meier (KM) curves for patients with low PD-L1 expression (CPS <10 or TPS <1%). Similar limitations have recently been addressed with a novel workflow, KMSubtraction, in the landscape of ICI use in advanced gastric and esophageal adenocarcinoma.^19^

With the aim of clarifying the survival benefit in the subgroup of patients with ESCC with low PD-L1 expression, after deriving individual patient data (IPD) from the reported KM curves, we investigated unreported PD-L1 subgroups in pivotal trials and conducted a series of IPD pooled analyses.

## Methods

### Study Selection

For this meta-analysis, a search was conducted on Scopus, Embase, PubMed, and Web of Science for RCTs from inception to October 1, 2021. The full search string is detailed in eTable 1 in the Supplement. Abstracts were reviewed by A.G.L. and resolved by F.P. This study included phase 3 prospective RCTs investigating the effectiveness of immunochemotherapy in advanced ESCC. If multiple publications of the same trial were retrieved, the most recent and informative publication was included. Data on race and ethnicity were not applicable to this study type and thus were not collected. This study was conducted in accordance with the Preferred Reporting Items for Systematic Reviews and Meta-analyses (PRISMA) reporting guideline for IPD.^20^

### Risk of Bias Assessment and Extraction of Reported KM Curves

Using the Cochrane Risk of Bias tool for RCTs, D.W.T.Y. and N.Z.H.W. evaluated the included trials for risk of bias.^21^ The KM curves of the all-comer population and subgroups with high PD-L1 expression were extracted from the trial articles. It was anticipated that not all trials had published the KM curves for the subgroups with low PD-L1 expression. Nonetheless, the KM curves from subgroups with low PD-L1 expression were extracted from the trials when possible and available. In addition, for trials that enrolled patients with both ESCC and esophageal adenocarcinoma, ESCC-specific subgroup data were extracted to avoid any potential confounding.

### Reconstruction of Time-to-Event Outcomes

Time-to-event outcomes were retrieved from all KM curves by methods described by Guyot et al^22^ and Liu et al.^23^ The quality of reconstruction was evaluated by inspecting the at-risk tables, hazard ratios (HRs), and shape of the KM curves.

For trials without available KM curves for the population with low PD-L1 expression, KMSubtraction was used to retrieve the survival data.^19,24^ KMSubtraction is a workflow to derive unreported subgroup survival data from known subgroups. In this instance, it was implemented to derive data for the subgroups with low PD-L1 expression from the data for all comers and the subgroups with high PD-L1 expression. Minimal-cost bipartite matching was used as the primary algorithm for matching. Monte Carlo simulations with 1000 iterations were conducted to determine the limits of error for KMSubtraction.

### Quality Assessment of Reconstructed Data

Before the pooled analysis was performed, the quality of the reconstruction was evaluated. Reconstructed KM curves for the all comers and for subgroups with high PD-L1 expression were compared with the original published KM curves. The KM curves were evaluated according to a visual comparison of curve shape, marginal HRs, and at-risk tables. KMSubtraction-derived KM curves and HRs for the subgroups with low PD-L1 expression were compared with the original published HRs.

To evaluate the effectiveness of matching, empirical cumulative distribution plots and Bland-Altman plots were used to demonstrate discrepancies in follow-up time between matched pairs. The KM curves of the matched cohorts were also plotted.

### One-Stage Pooled Analysis

To elucidate the magnitude of benefit in the subgroup of patients with low PD-L1 expression, 1-stage pooled analyses using derived IPD were conducted. A 1-stage pooled analysis was conducted for all first-line trials and was also repeated for second-line trials. In addition, because of the prevailing interest in the effect of different scoring systems on patient selection, first-line trials of studies using similar scoring systems and cutoffs—a CPS of 10 or TPS of 1%—were pooled in a 1-stage analysis.

In all analyses, the primary outcome was prespecified to be OS, and secondary outcomes included progression-free survival (PFS) and duration of response (DOR). To account for between-study heterogeneity, the shared-frailty model was used to incorporate a random-effects term. Gamma-distributed frailties were used. Hazard ratios were computed from a Cox proportional hazards regression model.

All analyses were conducted in R, version 4.1.0, using the survival, ggplot2, KMSubtraction, and IPDfromKM packages. Data analysis was conducted from January 1, 2022, to June 30, 2022. Cox proportional hazards regression was used to calculate 2-sided *P* values, with *P* < .05 used to determine statistical significance.

## Results

### Study Selection

The electronic search returned 984 potentially relevant articles. After deduplication and screening, 9 studies comprising 4752 patients were identified and included in the analysis (eFigure 1 in the Supplement).

### Baseline Characteristics of Trials Included

Nine trials were included in this analysis: CheckMate-648,^1^ ESCORT-1st,^2^ KEYNOTE-590,^3^ ORIENT-15,^4^ KEYNOTE-181,^5^ ESCORT,^6^ RATIONALE-302,^7^ ATTRACTION-3,^8^ and ORIENT-2.^9^ Six trials (CheckMate-648,^1^ ESCORT,^6^ KEYNOTE-181,^5^ KEYNOTE-590,^3^ ORIENT-15,^4^ and ORIENT-2^9^) reported HRs but not KM curves for the subgroup of patients with ESCC with low PD-L1 expression. Three trials (ESCORT-1st,^2^ RATIONALE-302,^7^ and ATTRACTION-3^8^) published KM curves and HRs. The characteristics of the trials with a breakdown of the reporting are summarized in eTable 2 in the Supplement.

Two trials (KEYNOTE-181^5^ and KEYNOTE-590^3^) enrolled patients with ESCC and esophageal adenocarcinoma. Only ESCC-specific data were extracted from these 2 trials to avoid any potential confounding. Six trials (CheckMate-648,^1^ ESCORT-1st,^2^ ESCORT,^6^ ATTRACTION-3,^8^ ORIENT-2,^9^ and ORIENT-15^4^) stratified results according to multiple cutoffs. However, only HRs were reported for intermediate outcomes, which precluded the reconstruction of time-to-event outcomes. A CPS of 10 and a TPS of 1% were the 2 cutoffs with sufficient KM curves to allow a pooled analysis.

### Reconstruction of Time-to-Event Outcomes

Time-to-event outcomes were reconstructed from the KM curves of all 9 trials for the all-comer population and the subgroup with high PD-L1 expression. Time-to-event reconstruction for the population with low PD-L1 expression depended on whether the trials originally reported the KM curves for this subgroup of patients. For the 6 trials (CheckMate-648,^1^ ESCORT,^6^ KEYNOTE-181,^5^ KEYNOTE-590,^3^ ORIENT-15,^4^ and ORIENT-2^9^) that reported HRs but not KM curves for the subgroup of patients with ESCC with low PD-L1 expression, time-to-event outcomes were derived with KMSubtraction. For the 3 trials (ESCORT-1st,^2^ RATIONALE-302,^7^ and ATTRACTION-3^8^) that published the KM curves, time-to-event outcomes were derived from the published subgroup with low PD-L1 expression. A detailed summary of where KMSubtraction was used to derive subgroup data is shown in eTable 2 in the Supplement.

### Quality Assessment of Trials and Reconstructed Data

The risk of bias was determined to be low for all studies (eFigure 2 in the Supplement). The graphic reconstruction algorithm derived IPD that resulted in HRs similar to those of the original reported curves of the all-comer subgroups and subgroups with high PD-L1 expression. A side-by-side comparison of the original curves and the reconstructed curves demonstrated a close match to original KM curves on visual inspection, marginal HRs, and comparisons of number-at-risk tables (eFigure 3 in the Supplement).

In terms of the subgroups with low PD-L1 expression, the KMSubtraction approach derived HRs similar to those previously reported. A side-by-side comparison of the original HRs and the KMSubtraction-derived curves is provided in eFigure 3 in the Supplement. As an illustration, the reported HR for the ORIENT-15 subgroup with low PD-L1 expression comparing sintilimab plus chemotherapy vs chemotherapy alone was 0.62 (95% CI, 0.45-0.85).^4^ The HR derived via KMSubtraction was 0.62 (95% CI, 0.45-0.85; *P* = .003) (eFigure 4 in the Supplement).

In terms of effectiveness of matching, good overlap between matched pairs was demonstrated on empirical cumulative distribution plots for each implementation of KMSubtraction. There were negligible mean absolute differences in follow-up time between matched pairs on the Bland-Altman plots (as seen by the clustering of data points near 0) (eFigure 5 in the Supplement). Converted limits of error were reasonable for each implementation of KMSubtraction (eFigure 6 in the Supplement). The quality assessment provided confidence to proceed with subsequent analysis.

### Overall Survival

**Figure 1.**
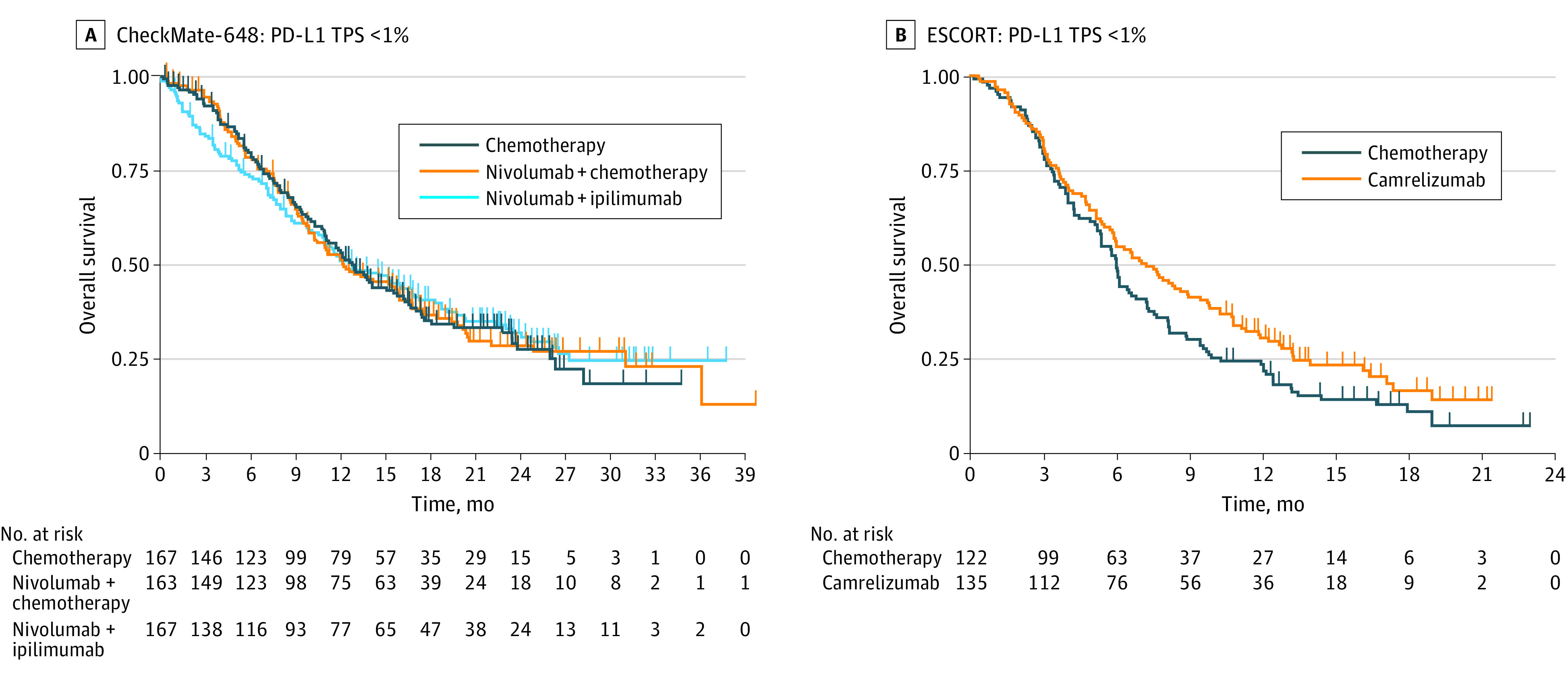
Kaplan-Meier Plots for Overall Survival in Low Programmed Death Ligand 1 (PD-L1) Subgroups Derived With KMSubtraction (Studies With the Tumor Proportion Score [TPS] Available) A, In the first-line CheckMate-648 study, there was no significant difference in overall survival for the subgroup with a TPS lower than 1% for both ipilimumab plus nivolumab dual immunotherapy (hazard ratio [HR], 0.98; 95% CI, 0.75-1.28; *P* = .89) and nivolumab-based immunochemotherapy (HR, 0.99; 95% CI, 0.76-1.30; *P* = .97) compared with chemotherapy alone. B, In the second-line ESCORT study, there was no statistically significant difference in overall survival for immunotherapy compared with chemotherapy alone (HR, 0.78; 95% CI, 0.59-1.02; *P* = .07) for the subgroup with a TPS lower than 1%.

In the derived subgroups with low PD-L1 expression in studies that used the TPS to determine the PD-L1 score, there was no difference in OS for immunotherapy-based groups compared with chemotherapy across all studies. In the first-line CheckMate-648 study, there was no significant difference in OS for the subgroup with a TPS lower than 1% both for ipilimumab-nivolumab dual immunotherapy (HR, 0.98; 95% CI, 0.75-1.28; *P* = .89) and for nivolumab-based immunochemotherapy (HR, 0.99; 95% CI, 0.76-1.30; *P* = .97) compared with chemotherapy alone (Figure 1A). In the second-line ESCORT study, there was no statistically significant difference in OS for immunotherapy compared with chemotherapy alone (HR, 0.78; 95% CI, 0.59-1.02; *P* = .07) for the subgroup with a TPS lower than 1% (Figure 1B).

**Figure 2.**
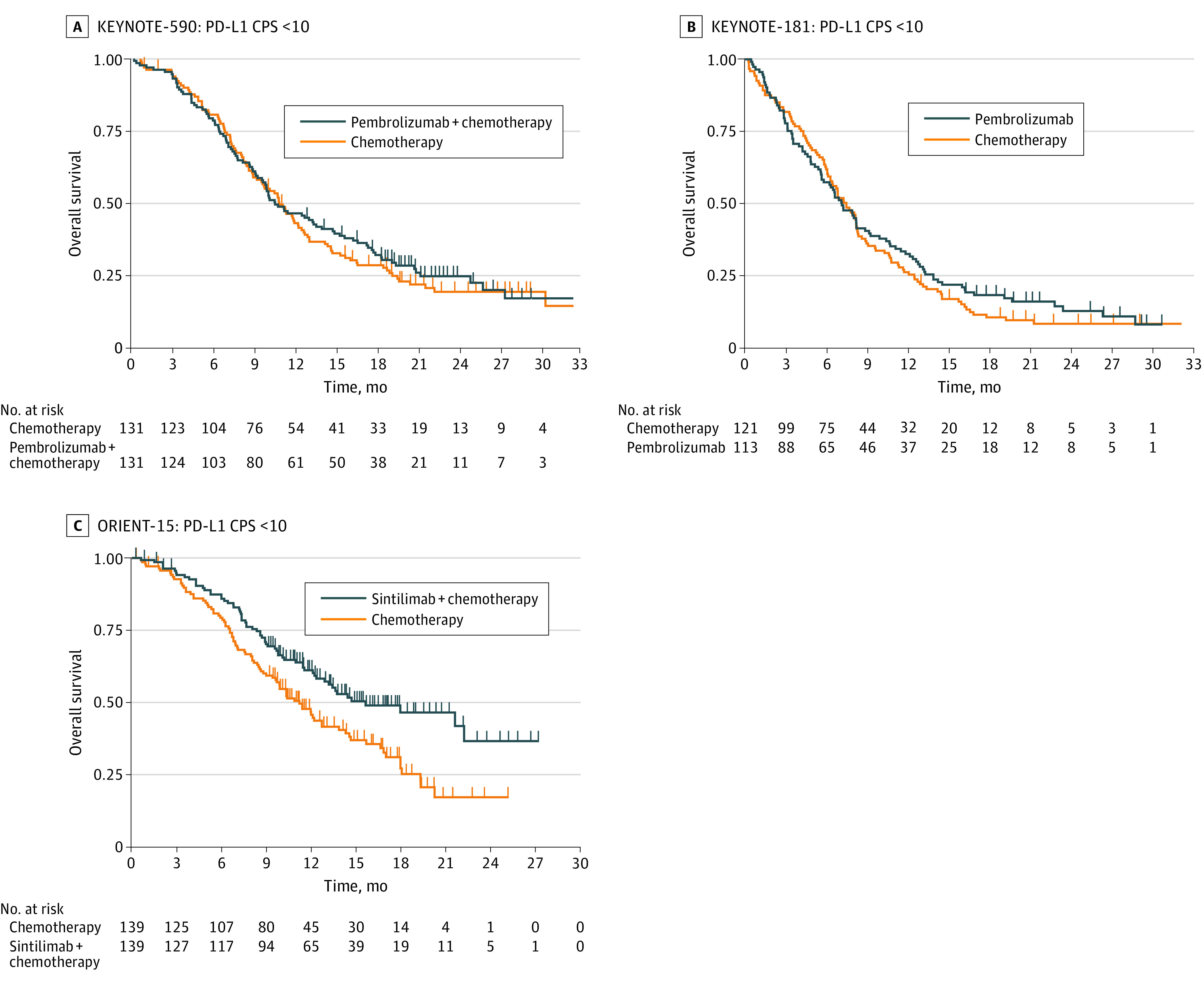
Kaplan-Meier Plots for Overall Survival in Low Programmed Death Ligand 1 (PD-L1) Subgroups Derived With KMSubtraction (Studies With the Combined Positive Score [CPS] Available) A, In the global multinational studies on pembrolizumab, the first-line KEYNOTE-590 trial subgroup with a CPS lower than 10 demonstrated no significant difference in overall survival for immunochemotherapy compared with chemotherapy alone (hazard ratio [HR], 0.91; 95% CI, 0.69-1.20; *P* = .50). B, In the second-line KEYNOTE-181 trial, for the subgroup with a CPS lower than 10, there was no significant difference in overall survival for immunotherapy compared with chemotherapy alone (HR, 0.90; 95% CI, 0.68-1.18; *P* = .44). C, In the sintilimab-based ORIENT-15 trial, for the subgroup with a CPS lower than 10, there was a significant difference in overall survival for immunochemotherapy compared with chemotherapy (HR, 0.62; 95% CI, 0.45-0.85; *P* = .003).

In studies that used the CPS to determine the PD-L1 score, there was heterogeneity in the OS results between immunotherapy- and chemotherapy-based groups. In the global multinational studies on pembrolizumab, the first-line KEYNOTE-590 trial subgroup with a CPS lower than 10 demonstrated no significant difference in OS for immunochemotherapy compared with chemotherapy alone (HR, 0.91; 95% CI, 0.69-1.20; *P* = .50) (Figure 2A). In the second-line KEYNOTE-181 trial, for the subgroup with a CPS lower than 10, there was no significant difference in OS for immunotherapy compared with chemotherapy alone (HR, 0.90; 95% CI, 0.68-1.18; *P* = .44) (Figure 2B). On the contrary, in the sintilimab-based ORIENT-15 trial, for the subgroup with a CPS lower than 10, there was a significant difference in OS for immunochemotherapy compared with chemotherapy (HR, 0.62; 95% CI, 0.45-0.85; *P* = .003) (Figure 2C).

### Progression-Free Survival

**Figure 3.**
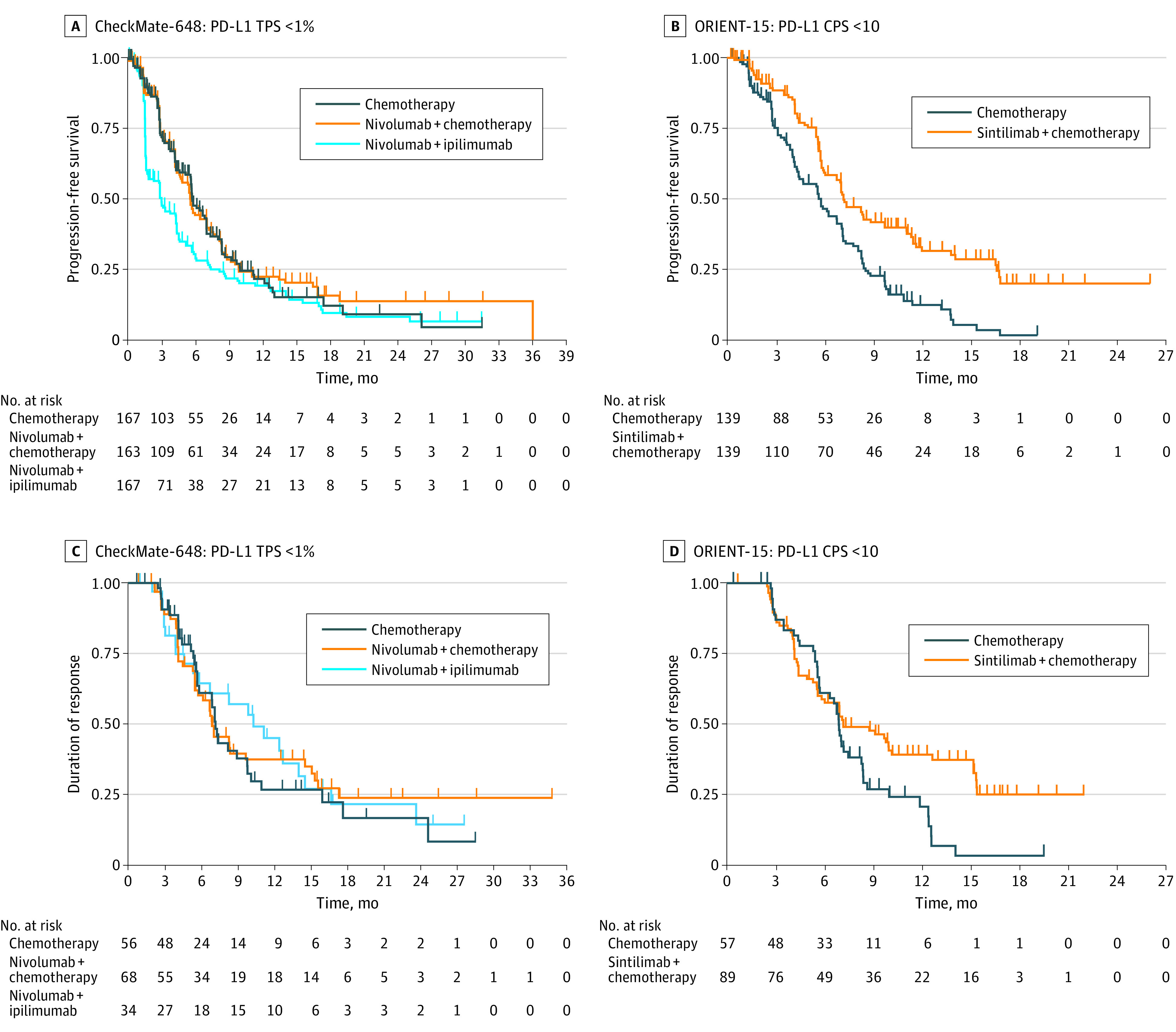
Kaplan-Meier Plots for Progression-Free Survival and Duration of Response in Low Programmed Death Ligand 1 (PD-L1) Subgroups Derived With KMSubtraction A, In the subgroup of CheckMate-648 with a tumor proportion score (TPS) lower than 1%, no significant difference in progression-free survival was observed between immunochemotherapy and chemotherapy (hazard ratio [HR], 0.98; 95% CI, 0.75-1.28; *P* = .88); there was a significantly inferior progression-free survival for ipilimumab plus nivolumab dual immunotherapy compared with chemotherapy alone (HR, 1.47; 95% CI, 1.14-1.90; *P* = .003). B, In the subgroup of ORIENT-15 with a combined positive score (CPS) lower than 10, a significant difference in progression-free survival was observed between immunochemotherapy and chemotherapy (HR, 0.52; 95% CI, 0.39-0.70; *P* < .001). C, In the subgroup of CheckMate-648 with a TPS lower than 1%, the median duration of response was 6.9 months (95% CI, 5.8-15.1 months; HR, 0.91; 95% CI, 0.58-1.44; *P* = .70) for nivolumab-based immunochemotherapy, 10.3 months (95% CI, 5.8-16.7 months; HR, 0.87; 95% CI, 0.51-1.49; *P* = .61) for nivolumab plus ipilimumab, and 7.2 months (95% CI, 5.8-10.1 months) for chemotherapy. D, In the subgroup of ORIENT-15 with a CPS lower than 10, the median duration of response was 7.2 months (95% CI, 5.8-15.2 months; HR, 0.64; 95% CI, 0.43-0.96; *P* = .03) for sintilimab-based immunochemotherapy and 6.9 months (95% CI, 5.7-8.4 months) for chemotherapy.

CheckMate-648 and ORIENT-15 reported sufficient supplementary data to enable derivation of PFS of the unreported subgroup. In the CheckMate-648 subgroup with a TPS lower than 1%, no significant difference in PFS was observed for immunochemotherapy vs chemotherapy (HR, 0.98; 95% CI, 0.75-1.28; *P* = .88). However, there was a significantly inferior PFS for ipilimumab plus nivolumab dual immunotherapy compared with chemotherapy alone (HR, 1.47; 95% CI, 1.14-1.90; *P* = .003) (Figure 3A). In the ORIENT-15 subgroup with a CPS lower than 10, a significant difference in PFS was observed for immunochemotherapy vs chemotherapy alone (HR, 0.52; 95% CI, 0.39-0.70; *P* < .001) (Figure 3B).

### Duration of Response

Similarly, CheckMate-648 and ORIENT-15 reported sufficient supplementary data to enable derivation of DOR in the unreported subgroup. In the CheckMate-648 subgroup with a TPS lower than 1%, the median DOR for nivolumab-based immunochemotherapy was 6.9 months (95% CI, 5.8-15.1 months; HR, 0.91; 95% CI, 0.58-1.44; *P* = .70) compared with chemotherapy. The DOR for the nivolumab-ipilimumab dual immunotherapy group was 10.3 months (95% CI, 5.8-16.7 months; HR, 0.87; 95% CI, 0.51-1.49; *P* = .61) compared with chemotherapy. The DOR for the chemotherapy group was 7.2 months (95% CI, 5.8-10.1 months) (Figure 3C). In the ORIENT-15 subgroup with a CPS lower than 10, the median DOR for sintilimab-based immunochemotherapy was 7.2 months (95% CI, 5.8-15.2 months; HR, 0.64; 95% CI, 0.43-0.96; *P* = .03) compared with chemotherapy. The DOR for the chemotherapy group was 6.9 months (95% CI, 5.7-8.4 months) (Figure 3D).

### One-Stage IPD-Pooled Analysis Based on Line of Therapy

The IPD pooled analysis of all first-line trials comprised CheckMate-648,^1^ ESCORT-1st,^2^ KEYNOTE-590,^3^ and ORIENT-15.^4^ There was a significant difference in OS for immunochemotherapy (HR, 0.70; 95% CI, 0.63-0.77; *P* < .001) compared with chemotherapy alone (eFigure 7A in the Supplement). There was a significant difference in PFS for immunochemotherapy (HR, 0.64; 95% CI, 0.58-0.70; *P* < .001) compared with chemotherapy alone (eFigure 7B in the Supplement).

The IPD pooled analysis of all second-line trials comprised KEYNOTE-181,^5^ ESCORT,^6^ RATIONALE-302,^7^ ATTRACTION-3,^8^ and ORIENT-2.^9^ There was a significant difference in OS for immunotherapy (HR, 0.72; 95% CI, 0.65-0.80; *P* < .001) compared with chemotherapy (eFigure 7C in the Supplement). There was a significant difference in PFS for immunotherapy (HR, 0.89; 95% CI, 0.81-0.99; *P* = .03) compared with chemotherapy alone (eFigure 7D in the Supplement).

### One-Stage IPD-Pooled Analysis Based on Scoring System

**Figure 4.**
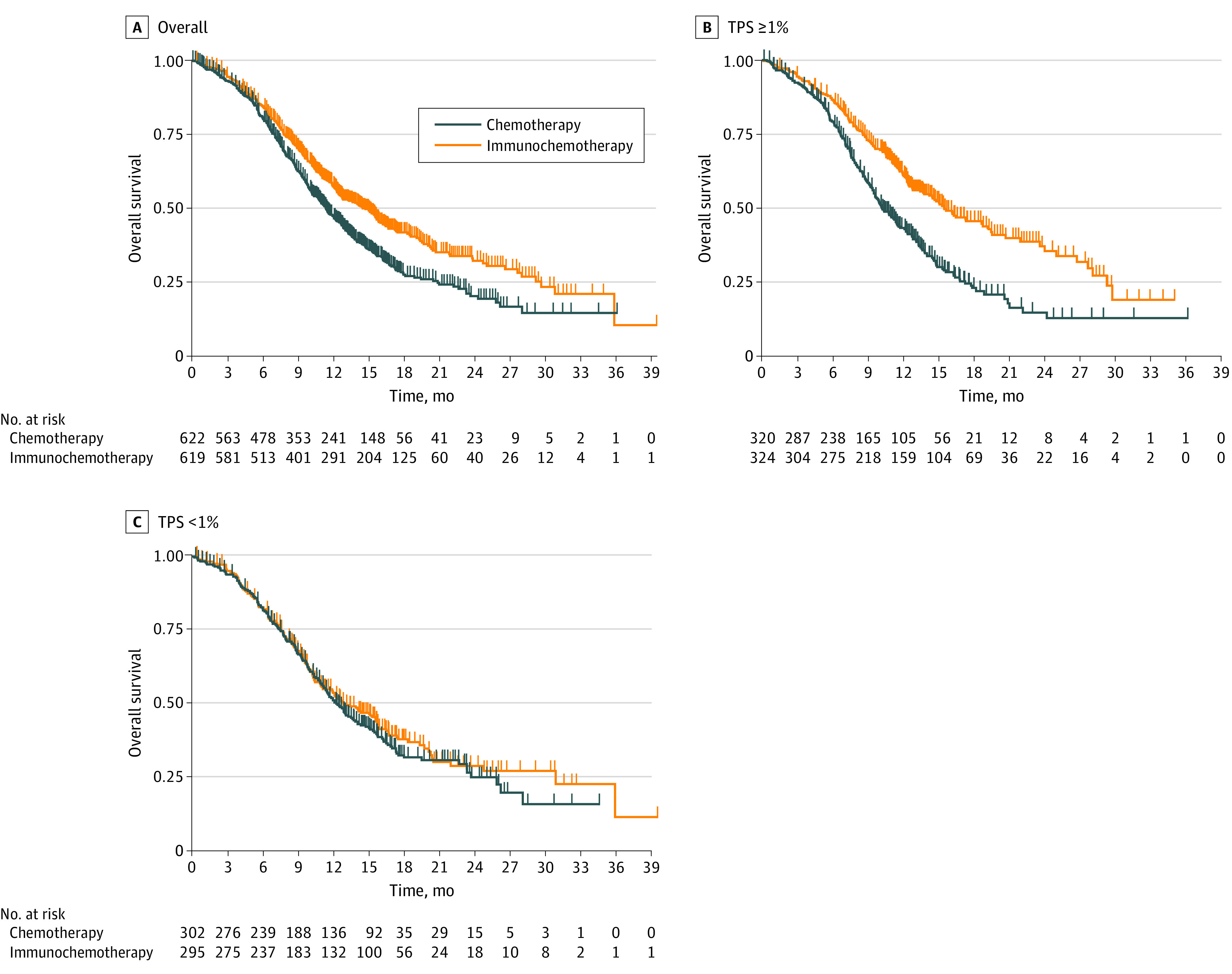
One-Stage Pooled Analysis of First-line Studies With the Tumor Proportion Score (TPS) Available (CheckMate-648 and ESCORT-1st) A, There was a significant difference in overall survival for the overall population comparing immunochemotherapy (hazard ratio [HR], 0.73; 95% CI, 0.63-0.84; *P* < .001) with chemotherapy alone. B, There was a significant difference in overall survival for the population with a TPS of 1% or higher comparing immunochemotherapy (HR, 0.57; 95% CI, 0.47-0.70; *P* < .001) with chemotherapy alone. C, There was no significant difference in overall survival for the subgroup with a TPS lower than 1% comparing immunochemotherapy (HR, 0.91; 95% CI, 0.74-1.12; *P* = .38) with chemotherapy alone.

Studies with similar PD-L1 scoring systems and cutoffs (CPS or TPS) were pooled in an IPD analysis. The 2 cutoffs of interest were a TPS of 1% and CPS of 10. In the IPD meta-analysis of first-line trials that evaluated PD-L1 expression based on TPS (CheckMate-648 and ESCORT-1st), there was a significant difference in OS for the overall population comparing immunochemotherapy (HR, 0.73; 95% CI, 0.63-0.84; *P* < .001) with chemotherapy alone (Figure 4A). Similarly, there was a significant difference in OS for the subgroup with a TPS of 1% or higher comparing immunochemotherapy (HR, 0.57; 95% CI, 0.47-0.70; *P* < .001) with chemotherapy alone (Figure 4B). However, there was no significant difference in OS for the subgroup with a TPS lower than 1% in comparing immunochemotherapy (HR, 0.91; 95% CI, 0.74-1.12; *P* = .38) with chemotherapy alone (Figure 4C).

**Figure 5.**
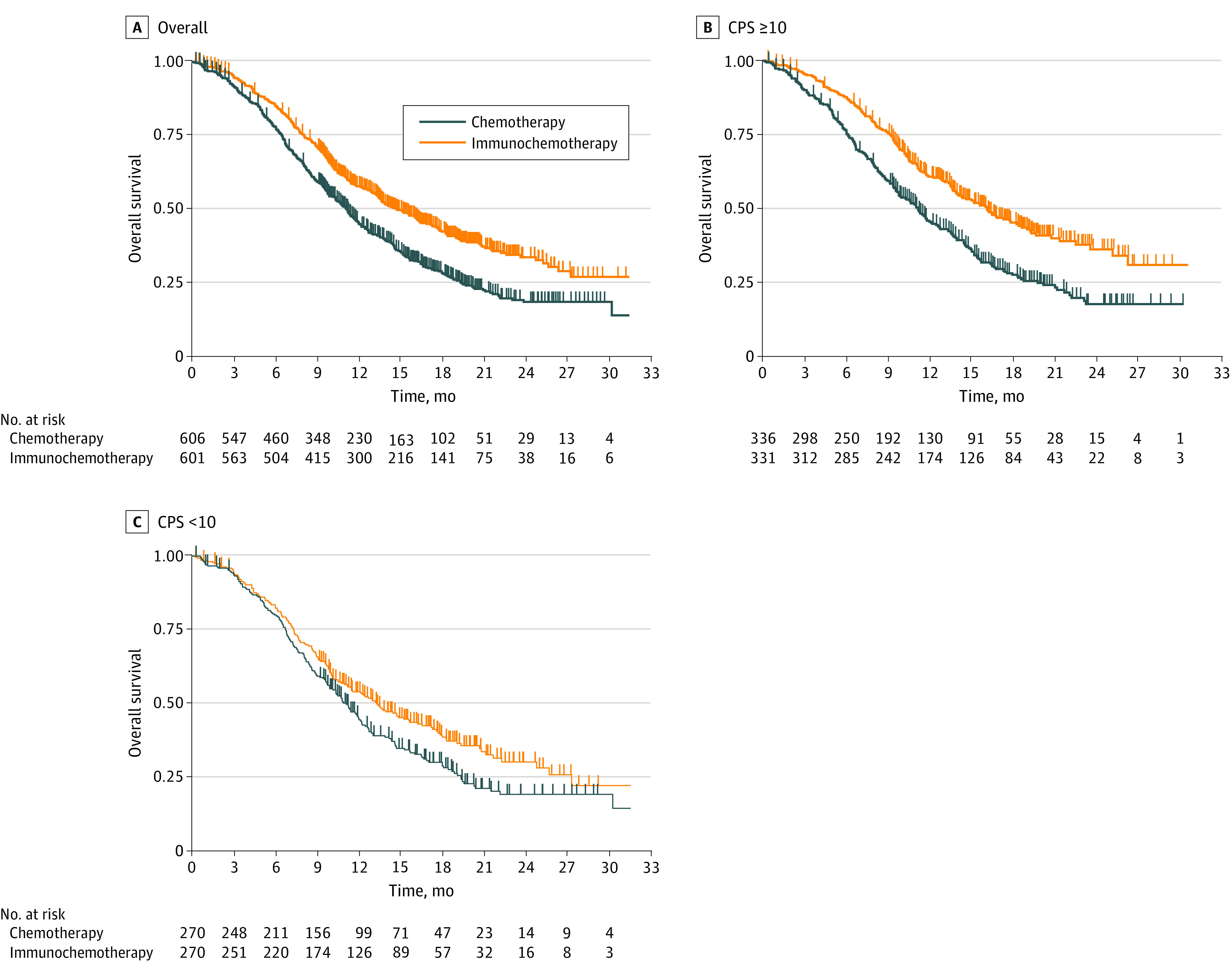
One-Stage Pooled Analysis of First-line Combined Positive Score (CPS) Studies (ORIENT-15 and KEYNOTE-590) A, There was a significant difference in overall survival for the overall group comparing immunochemotherapy (hazard ratio [HR], 0.67; 95% CI, 0.58-0.78; *P* < .001) with chemotherapy alone. B, There was a significant difference in overall survival for the group with a CPS of 10 or higher comparing immunochemotherapy (HR, 0.60; 95% CI, 0.49-0.73; *P* < .001) with chemotherapy alone. C, There was a significant difference in overall survival for the subgroup with a CPS lower than 10 comparing immunochemotherapy with chemotherapy alone (HR, 0.77; 95% CI, 0.62-0.94; *P* = .01).

In the IPD meta-analysis of first-line trials that evaluated PD-L1 expression based on CPS (ORIENT-15 and KEYNOTE-590), there was a significant difference in OS for the overall population comparing immunochemotherapy (HR, 0.67; 95% CI, 0.58-0.78; *P* < .001) with chemotherapy alone (Figure 5A). Similarly, there was also a significant difference in OS for the subgroup with a CPS of 10 or higher comparing immunochemotherapy (HR, 0.60; 95% CI, 0.49-0.73; *P* < .001) with chemotherapy alone (Figure 5B). There was also a significant difference in OS for the subgroup with a CPS lower than 10 comparing immunochemotherapy with chemotherapy (HR, 0.77; 95% CI, 0.62-0.94; *P* = .01) (Figure 5C).

## Discussion

Multiple RCTs have demonstrated the effectiveness of immunotherapy for patients with advanced ESCC, particularly for those with high PD-L1 expression. However, there remains uncertainty about the effectiveness of immunotherapy in the subgroup of patients with low or negative PD-L1 expression. Survival data for the subgroups with low PD-L1 expression have not been consistently reported in all trials because they were not part of the original planned analysis. Hence, we used KMSubtraction to derive these data and perform a pooled analysis with IPD. Our analysis revealed a trend toward lower effectiveness for patients with ESCC with low PD-L1 expression. Compared with the overall population or subgroups with high PD-L1 expression, there was no statistically significant difference in OS between anti–PD-1–based regimens vs chemotherapy alone in most populations with low PD-L1 expression, particularly in trials that measured PD-L1 by using TPS.

The results in this study suggest that PD-L1 expression is an important biomarker in selecting patients for immunotherapy. When the results for studies that used the TPS to score PD-L1 were pooled, first-line immunochemotherapy or dual immunotherapy did not provide a significant OS or PFS benefit in the subgroup with a TPS lower than 1%, which suggests that the results reported in the overall populations may be explained by the positive results in the subgroup of patients with a TPS of 1% or higher. In the subgroup with a TPS lower than 1%, neither tail of the survival curves diverged by long-term time points, suggesting that the number of patients who experience meaningful benefit from immunotherapy may be extremely limited. This finding suggests that the TPS cutoff of 1% or higher may be used to enrich patients with benefit from immunotherapy.

The DOR in the immunochemotherapy group of CheckMate-648 was superimposable with the chemotherapy group in the subgroup with a TPS lower than 1%, which suggests that the response was largely associated with chemotherapy or may have been due to a transient, nonsustained immunotherapy effect. These data are similar to those for patients with gastroesophageal adenocarcinoma who were enrolled in the CheckMate-649 trial.^25^

The situation is less straightforward for studies using a CPS cutoff of 10 given that there is some discordance between the trials. Although the pooled analysis of these studies using a CPS cutoff of 10 (ORIENT-15 and KEYNOTE-590) showed a significant benefit in the subgroup with a CPS lower than 10, it may be explained by the positive results in ORIENT-15. As such, additional research is needed to identify patients with a CPS lower than 10 who have a chance of benefit. The OS benefit observed in Asian studies (RATIONALE-302 and ORIENT-15) for patients with low PD-L1 expression as determined by the CPS, in contrast to the global pembrolizumab-based first- and second-line studies (KEYNOTE-590 and KEYNOTE-181), suggests that factors beyond PD-L1 may also determine the effectiveness of immunotherapy, such as geographic variation or molecular subtypes.^26^ Given that some patients with low PD-L1 expression experience clinically meaningful benefit, there is a need to explore additional predictive biomarkers, such as tumor mutational burden, immune signatures, and gut microbiota, which may need to be used in tandem with PD-L1 to identify responders.^27,28,29^

Clinicians should be aware of the differences between the TPS and CPS as methods of measuring PD-L1 expression. Recent work^30^ has suggested that PD-L1 expression fluctuates temporally by site of tumor biopsy or prior chemotherapy. New biopsies for patients with low PD-L1 expression may need to be considered. There exists uncertainty over the harmonization of PD-L1 assays, with one recent study^31^ showing positivity rates differing by up to 2 times when different PD-L1 immunohistochemistry assays were used. The issue of cutoffs may be of relevance because PD-L1 expression should be regarded as a continuous variable, and higher CPS cutoffs may identify patients with meaningful benefit more accurately. A recent study^32^ showed that a CPS cutoff of 20 may identify patients with advanced or recurrent head and neck squamous cell carcinoma who may derive long-term and meaningful benefit from first-line pembrolizumab-based regimens.

### Limitations

Our findings should be interpreted with caution. Although the *P* values are suggestive of nonstatistical significance in subgroups with low PD-L1 expression, these analyses were not predefined end points of the original trials. Hence, some of the individually reported subgroups with low PD-L1 expression may not be adequately powered to allow definitive conclusions specific to the original trials due to small subgroup sizes.

Although methodological precautions (through quality checks) have been taken to ensure that the derived KM curves and HRs for the low PD-L1 expression subgroups are identical, or as close to the reported HRs as possible, we acknowledge some minute differences. These may be attributed to slight differences in censoring or patient-level covariates that are impossible to account for given the lack of participant-level data and the nature of univariate survival models.

Finally, where possible and available, we advise clinicians to refer to the published observed data or future post hoc analysis from the original trials. Despite these limitations, this study demonstrated lower effectiveness of immunotherapy for patients with ESCC with low PD-L1 expression.

## Conclusions

In this meta-analysis, findings showed that patients with ESCC and low or negative PD-L1 expression who are treated with anti–PD-1–based regimens may not be conferred a survival advantage, consistent with the EMA approval of nivolumab plus chemotherapy for patients with ESCC and a TPS of 1% or higher. These data suggest that, for patients with ESCC and low PD-L1 expression, it would be prudent for clinicians to emphasize the uncertainty of benefit with the addition of immunotherapy or to minimize additional toxic effects and economic burden by avoiding immunotherapy altogether.

## Notes

Supplement. **eTable 1.** Search Information **eTable 2.** Search Information and Where KMSubtraction Was Implemented **eFigure 1.** PRISMA Diagram **eFigure 2. **Risk-of-Bias Assessment **eFigure 3. **Comparisons to Original Curves for Overall and PD-L1–High Subgroups **eFigure 4.** Example Comparisons of KMSubtraction Outcomes With Reported HRs for PD-L1–Low Subgroups **eFigure 5. **Evaluation of KMSubtraction Bipartite Matching **eFigure 6.** Convergence Plots and Histograms of Simulations **eFigure 7.** One-Stage Pooled Analysis of First-line and Second-line Studies
