# Supplementary material for: Effectiveness of Immune Checkpoint Inhibitors in Patients With Advanced Esophageal Squamous Cell Carcinoma: A Meta-analysis Including Low PD-L1 Subgroups
Source: JAMA Oncol. 2022 Dec 8;9(2):215–24. doi: 10.1001/jamaoncol.2022.5816 (PMC9857522; doi:10.1001/jamaoncol.2022.5816)
Supplement: Supplement. — eTable 1. Search Information eTable 2. Search Information and Where KMSubtraction Was Implemented eFigure 1. PRISMA Diagram eFigure 2. Risk-of-Bias Assessment eFigure 3. Comparisons to Original Curves for Overall and PD-L1–High Subgroups eFigure 4. Example Comparisons of KMSubtraction Outcomes With Reported HRs for PD-L1–Low Subgroups eFigure 5. Evaluation of KMSubtraction Bipartite Matching eFigure 6. Convergence Plots and Histograms of Simulations eFigure 7. One-Stage Pooled Analysis of First-line and Second-line Studies [file jamaoncol-e225816-s001.pdf]

Supplementary Online Content

Yap DWT, Leone AG, Wong NZH, et al. Efficacy of immune checkpoint inhibitors in patients with advanced esophageal squamous cell carcinoma: a meta-analysis including low PD-L1 subgroups. *JAMA Oncol*. Published online December 8, 2022. doi:10.1001/jamaoncol.2022.5816

eTable 1. Search Information
eTable 2. Search Information and Where KMSubtraction Was Implemented
eFigure 1. PRISMA Diagram
eFigure 2. Risk-of-Bias Assessment
eFigure 3. Comparisons to Original Curves for Overall and PD-L1–High Subgroups
eFigure 4. Example Comparisons of KMSubtraction Outcomes With Reported HRs for PD-L1–Low Subgroups
eFigure 5. Evaluation of KMSubtraction Bipartite Matching
eFigure 6. Convergence Plots and Histograms of Simulations
eFigure 7. One-Stage Pooled Analysis of First-line and Second-line Studies

This supplementary material has been provided by the authors to give readers additional information about their work.

© 2022 Yap DWT et al. *JAMA Oncology*.

eTable 1. Search Information

**Date of search**	1 October 2021
**Databases**	PubMed, Web of Science, Scopus, Embase, American Society of Clinical Oncology Meeting Library
**Search string**	(gastric OR stomach OR esophageal OR gastroesophageal OR gastro-oesophageal OR gastro-esophageal) AND (checkpoint inhibitors OR ICI OR pembrolizumab OR ipilimumab OR nivolumab OR avelumab OR camrelizumab OR durvalumab OR sintilimab OR programmed death ligand OR "PD-L1" OR "PD-1" OR immunotherapy) AND (random* AND trial)
**Search string exploded**	("gastrics"[All Fields] OR "stomach"[MeSH Terms] OR "stomach"[All Fields] OR "gastric"[All Fields] OR ("stomach"[MeSH Terms] OR "stomach"[All Fields] OR "stomachs"[All Fields] OR "stomach s"[All Fields] OR "stomachal"[All Fields] OR "stomaches"[All Fields]) OR ("esophageal"[All Fields] OR "esophagic"[All Fields] OR "esophagitis"[MeSH Terms] OR "esophagitis"[All Fields] OR "esophagitides"[All Fields] OR "oesophagal"[All Fields] OR "oesophageal"[All Fields] OR "oesophagic"[All Fields] OR "oesophagitis"[All Fields]) OR ("gastro esophageal"[All Fields] OR "gastro oesophageal"[All Fields] OR "gastro oesophagitis"[All Fields] OR "gastroesophagal"[All Fields] OR "gastroesophageal"[All Fields] OR "gastroesophagic"[All Fields] OR "gastroesophagitis"[All Fields] OR "gastrooesophageal"[All Fields]) OR ("gastro esophageal"[All Fields] OR "gastro oesophageal"[All Fields] OR "gastro oesophagitis"[All Fields] OR "gastroesophagal"[All Fields] OR "gastroesophageal"[All Fields] OR "gastroesophagic"[All Fields] OR "gastroesophagitis"[All Fields] OR "gastrooesophageal"[All Fields]) OR ("gastro esophageal"[All Fields] OR "gastro oesophageal"[All Fields] OR "gastro oesophagitis"[All Fields] OR "gastroesophagal"[All Fields] OR "gastroesophageal"[All Fields] OR "gastroesophagic"[All Fields] OR "gastroesophagitis"[All Fields] OR "gastrooesophageal"[All Fields])) AND ((("cell cycle checkpoints"[MeSH Terms] OR ("cell"[All Fields] AND "cycle"[All Fields] AND "checkpoints"[All Fields]) OR "cell cycle checkpoints"[All Fields] OR "checkpoint"[All Fields] OR "checkpoints"[All Fields]) AND ("antagonists and inhibitors"[MeSH Subheading] OR ("antagonists"[All Fields] AND "inhibitors"[All Fields]) OR "antagonists and inhibitors"[All Fields] OR "inhibitors"[All Fields] OR "inhibitor"[All Fields] OR "inhibitor s"[All Fields])) OR "ICI"[All Fields] OR ("pembrolizumab"[Supplementary Concept] OR "pembrolizumab"[All Fields]) OR ("ipilimumab"[MeSH Terms] OR "ipilimumab"[All Fields]) OR ("nivolumab"[MeSH Terms] OR "nivolumab"[All Fields] OR "nivolumab s"[All Fields]) OR ("avelumab"[Supplementary Concept] OR "avelumab"[All Fields]) OR ("camrelizumab"[Supplementary Concept] OR "camrelizumab"[All Fields]) OR ("durvalumab"[Supplementary Concept] OR "durvalumab"[All Fields]) OR ("sintilimab"[Supplementary Concept] OR "sintilimab"[All Fields]) OR (("program"[All Fields] OR "program s"[All Fields] OR "programe"[All Fields] OR "programed"[All Fields] OR "programes"[All Fields] OR "programing"[All Fields] OR "programmability"[All Fields] OR "programmable"[All Fields] OR "programmably"[All Fields] OR "programme"[All Fields] OR "programme s"[All Fields] OR "programmed"[All Fields] OR "programmer"[All Fields] OR "programmer s"[All Fields] OR "programmers"[All Fields] OR "programmes"[All Fields] OR "programming"[All Fields] OR "programmings"[All Fields] OR "programs"[All Fields]) AND ("death"[MeSH Terms] OR "death"[All Fields] OR "deaths"[All Fields]) AND ("ligand s"[All Fields] OR "liganded"[All Fields] OR "liganding"[All Fields] OR "ligands"[MeSH Terms] OR "ligands"[All Fields] OR "ligand"[All Fields])) OR "PD-L1"[All Fields] OR "PD-1"[All Fields] OR ("immunotherapy"[MeSH Terms] OR "immunotherapy"[All Fields] OR "immunotherapies"[All Fields] OR "immunotherapy s"[All Fields])) AND ("random*"[All Fields] AND ("clinical trials as topic"[MeSH Terms] OR ("clinical"[All Fields] AND "trials"[All Fields] AND "topic"[All Fields]) OR "clinical trials as topic"[All Fields] OR "trial"[All Fields] OR "trial s"[All Fields] OR "trialed"[All Fields] OR "trialing"[All Fields] OR "trials"[All Fields]))

© 2022 Yap DWT et al. *JAMA Oncology*.

eTable 2. Search Information and Where KMSubtraction Was Implemented

Trial,	PD-L1 assay and reporting method	Comparison	Outcome	Histology	"Overall" curve	"Subgroup" High PD-L1 expressing curve	Did the original publication report the low PD-L1 KM curve?	Did the original publication report the low PD-L1 Hazard ratio?	PD-L1 low curves derived by KMSubtraction: i.e. "Subtracting" "Subgroup" curve from "overall" curve
ATTRACTION-3<br>Kato, 2019<br>NCT02569242	TPS ≥1 :Positive for PD-L1 when cancerous cells with stained cell membranes account for ≥1% of at least 100 evaluable cancerous cells<br>IHC 28-8 pharmDx assay (Dako, Santa Clara, CA, USA)	Nivo/Chemo	OS	ESCC	Overall	PD-L1 TPS ≥1	Yes	Yes	NIL as originally reported in manuscript
CheckMate-648<br>Yuichiro, 2022<br>NCT03143153	TPS ≥ 1%: Percentage of tumor cells with partial or complete membrane staining in at least 100 viable TCs<br>IHC 28-8 pharmDx assay (Dako, Santa Clara, CA, USA)<br>*CPS also was retrospectively generated in the original trial. However, main KM curves were stratified based on TPS only	Nivo+Chemo/Chemo	OS	ESCC	Overall	PD-L1 TPS ≥1	No	Yes	PD-L1 TPS <1
		Nivo+Ipi/Chemo	OS	ESCC	Overall	PD-L1 TPS ≥1	No	Yes	PD-L1 TPS <1
		Nivo+Chemo/Chemo	PFS	ESCC	Overall	PD-L1 TPS ≥1	No	Yes	PD-L1 TPS <1
		Nivo+Ipi/Chemo	PFS	ESCC	Overall	PD-L1 TPS ≥1	No	Yes	PD-L1 TPS <1
		Nivo+Chemo/Chemo	DOR	ESCC	Overall	PD-L1 TPS ≥1	No	NA	PD-L1 TPS <1
		Nivo+Ipi/Chemo	DOR	ESCC	Overall	PD-L1 TPS ≥1	No	NA	PD-L1 TPS <1
ESCORT<br>Huang, 2020<br>NCT03099382	TPS ≥1%: Percentage of viable tumor cells showing partial or complete membrane staining<br>IHC 6E8 Abcam (Shuwen Biotech, Deqing, Zhejiang, China)	Camre/Chemo	OS	ESCC	Overall	PD-L1 TPS ≥1	No	Yes	PD-L1 TPS <1
ESCORT 1st<br>Luo, 2021<br>NCT03691090	TPS ≥1%: Percentage of viable tumor cells showing partial or complete membrane staining, relative to all viable tumor cells present in the sample (positive and negative)<br>IHC 6E8 Abcam (Shuwen Biotech, Deqing, Zhejiang, China)	Camre+Chemo/ Chemo	OS	ESCC	Overall	PD-L1 TPS ≥1	Yes	Yes	NIL as originally reported in manuscript
		Camre+Chemo/ Chemo	PFS	ESCC	Overall	PD-L1 TPS ≥1	Yes	Yes	NIL as originally reported in manuscript
		Camre+Chemo/ Chemo	DOR	ESCC	Overall	PD-L1 TPS ≥1	Yes	NA	NIL as originally reported in manuscript

© 2022 Yap DWT et al. *JAMA Oncology*.

KN181<br>Kojima, 2020<br>NCT02559687	CPS: defined as the number of PD-L1–positive cells (tumor cells, macrophages, and lymphocytes) divided by the total number of tumor cells, multiplied by 100<br><br>IHC 22C3 pharmDx assay (Agilent Technologies, Carpinteria, CA, USA)	Pembro/Chemo	OS	ESCC	Overall	PD-L1 CPS ≥10	No	Yes	PD-L1 CPS <10
KN590<br>Sun, 2021<br>NCT03189719	CPS: defined as the number of PD-L1–positive cells (tumor cells, macrophages, and lymphocytes) divided by the total number of tumor viable cells<br><br>IHC 22C3 pharmDx assay (Agilent Technologies, Carpinteria, CA, USA)	Pembro+Chemo/Chemo	OS	ESCC	Overall	PD-L1 CPS ≥10	No	No	PD-L1 CPS <10
ORIENT-2<br>Xu, 2022<br>NCT03116152	IHC 22C3 pharmDx assay (Dako, Santa Clara, CA, USA)<br><br>*Results for CPS and TPS subgroups were reported as hazard ratios only. Curves were only stratified based on NLR/ TCR clonality.	Sinti/Chemo	OS	ESCC	Overall	KM curves not reported (only HRs presented)	No	No	Not possible as only overall curve published, PD-L1 high subgroup curve not reported
ORIENT-15<br>Lu, 2022<br>NCT03748134	CPS: defined as the number of PD-L1 staining cells (tumor cells, macrophages, and lymphocytes) divided by the total number of tumor cells<br><br>IHC 22C3 pharmDx assay (Agilent Technologies, Carpinteria, CA, USA)	Sinti+Chemo/Chemo	OS	ESCC	Overall	PD-L1 CPS ≥10	No	Yes	PD-L1 CPS <10
		Sinti+Chemo/Chemo	PFS	ESCC	Overall	PD-L1 CPS ≥10	No	Yes	PD-L1 CPS <10
		Sinti+Chemo/Chemo	DOR	ESCC	Overall	PD-L1 CPS ≥10	No	NA	PD-L1 CPS <10
RATIONALE 302<br>Lin, 2022<br>NCT03430843	TAP: Total percentage of the tumor area covered by tumor cells with any membrane staining above background and tumor-associated immune cells with any staining above background.<br><br>IHC SP263 assay (Ventana)	Tisle/Chemo	OS	ESCC	Overall	PD-L1 TAP ≥10	Yes	Yes	NIL as originally reported in manuscript

Abbreviations: Nivo, nivolumab; Ipi, ipilimumab; Camre, camrelizumab; Pembro, pembrolizumab; Sinti, sintilimab; Tisle, Tislelizumab; Chemo, chemotherapy; PD-L1, programmed death ligand 1; ESCC, esophageal squamous cell carcinoma; CPS, combined positive score; TPS, tumor proportion score; TAP, tumor area positivity; CM648, CheckMate 648; KN590, KEYNOTE-590; KN181, KEYNOTE-181.

© 2022 Yap DWT et al. *JAMA Oncology*.

eFigure 1. PRISMA Diagram

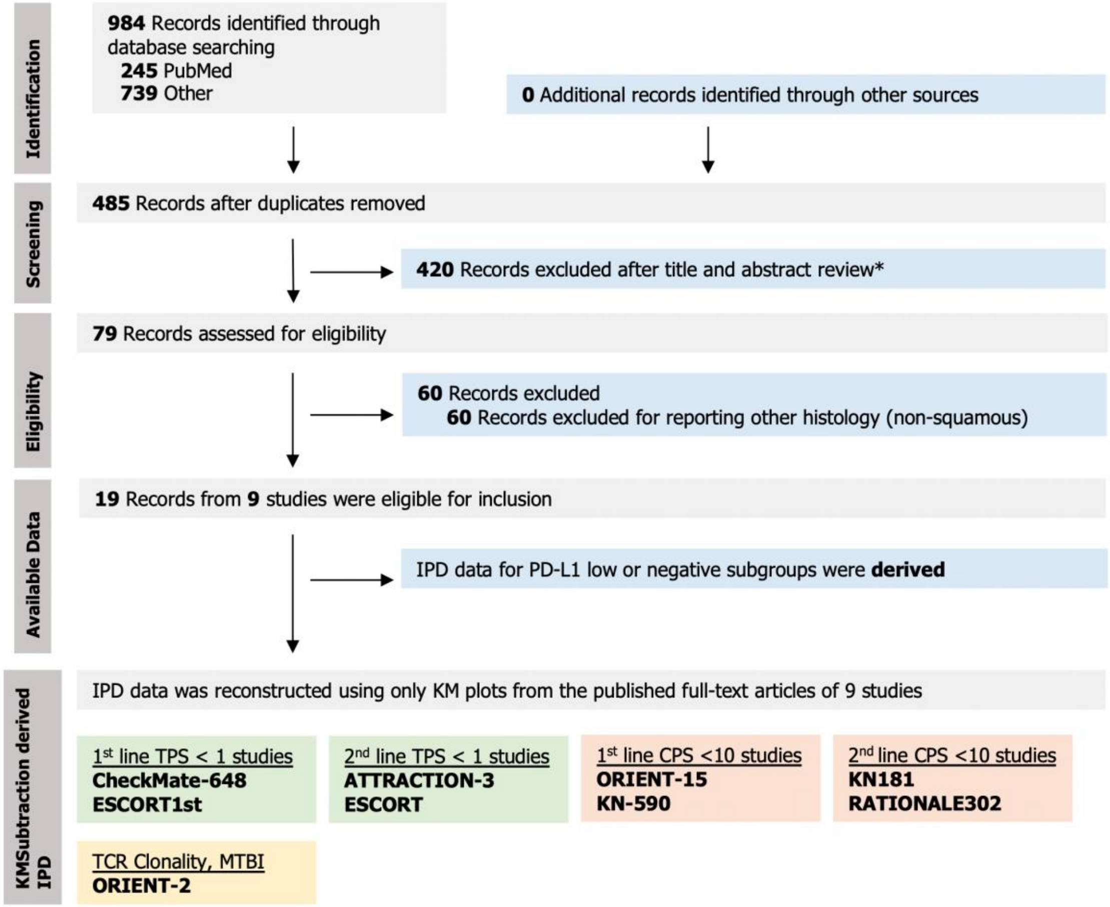

PRISMA, preferred reporting items for systematic reviews and meta-analyses; PD-L1, programmed death ligand 1; CPS, combined positive score; TPS, Tumor Proportion Score; IPD, individual patient data; KM, Kaplan-Meier; ESCC, esophageal squamous cell carcinoma; TCR, T-Cell Receptor; MTBI, Molecular Tumor Burden Index

© 2022 Yap DWT et al. *JAMA Oncology*.

eFigure 2. Risk-of-Bias Assessment

Risk of bias domains

Study	D1	D2	D3	D4	D5	Overall
CM648	+	+	+	+	+	+
ESCORT1st	+	+	+	+	+	+
KN181	+	+	+	+	+	+
RATIONALE302	+	+	+	+	+	+
ORIENT15	+	+	+	+	+	+
KN590	+	+	+	+	+	+
ATR3	+	+	+	+	+	+
ESCORT	+	+	+	+	+	+
ORIENT2	+	+	+	+	+	+

Domains:
D1: Risk of bias arising from the randomization process
D2: Risk of bias due to deviations from the intended interventions
D3: Risk of bias due to missing outcome data
D4: Risk of bias in the measurement of the outcome
D5: Risk of bias in selection of the reported result

Judgement:
+ The study is judged to be at low risk of bias for all domains for this result

D, domain; CM648, CheckMate-648; KN181, KEYNOTE-181; KN590, KEYNOTE-590; ATR3, ATTRACTION-3

© 2022 Yap DWT et al. *JAMA Oncology*.

eFigure 3. Comparisons to Original Curves for Overall and PD-L1–High Subgroups

Study, outcome, cohort	Original	Reconstructed
**ATTRACTION 3, Overall survival, overall cohort**	The original figure may be found in the primary trial manuscript<br><br>Kato K, Cho BC, Takahashi M, Okada M, Lin CY, Chin K, Kadowaki S, Ahn MJ, Hamamoto Y, Doki Y, Yen CC, Kubota Y, Kim SB, Hsu CH, Holtved E, Xynos I, Kodani M, Kitagawa Y. Nivolumab versus chemotherapy in patients with advanced oesophageal squamous cell carcinoma refractory or intolerant to previous chemotherapy (ATTRACTION-3): a multicentre, randomised, open-label, phase 3 trial. Lancet Oncol. 2019 Nov;20(11):1506-1517. doi: 10.1016/S1470-2045(19)30626-6. Epub 2019 Sep 30. Erratum in: Lancet Oncol. 2019 Nov;20(11):e613. PMID: 31582355.<br><br>Figure 2A	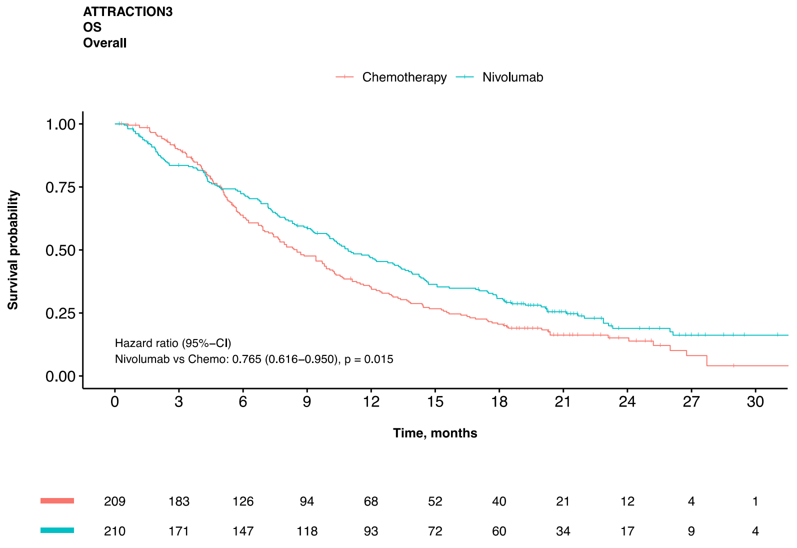

© 2022 Yap DWT et al. *JAMA Oncology*.

**ATTRACTION 3, overall survival, TPS >1%**	The original figure may be found in the primary trial manuscript<br><br>Kato K, Cho BC, Takahashi M, Okada M, Lin CY, Chin K, Kadowaki S, Ahn MJ, Hamamoto Y, Doki Y, Yen CC, Kubota Y, Kim SB, Hsu CH, Holtved E, Xynos I, Kodani M, Kitagawa Y. Nivolumab versus chemotherapy in patients with advanced oesophageal squamous cell carcinoma refractory or intolerant to previous chemotherapy (ATTRACTION-3): a multicentre, randomised, open-label, phase 3 trial. Lancet Oncol. 2019 Nov;20(11):1506-1517. doi: 10.1016/S1470-2045(19)30626-6. Epub 2019 Sep 30. Erratum in: Lancet Oncol. 2019 Nov;20(11):e613. PMID: 31582355.<br><br>Figure S2B	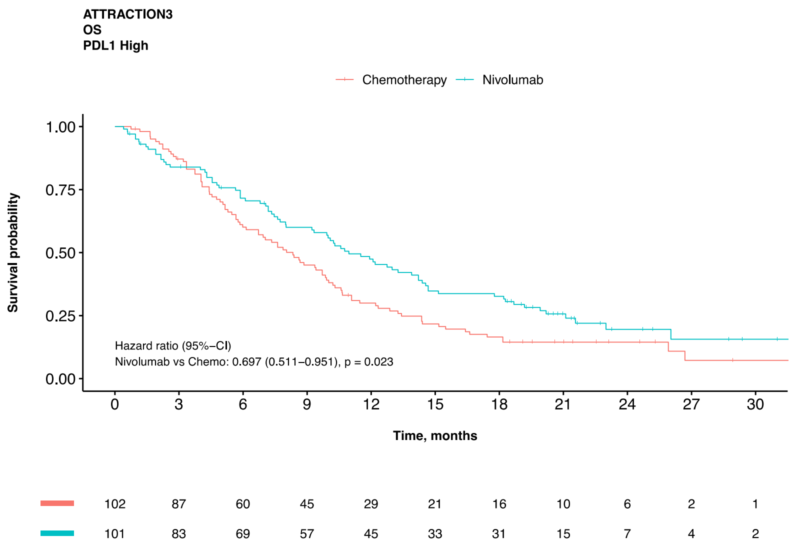

© 2022 Yap DWT et al. *JAMA Oncology*.

<table>
<tr>
<td>CM648, overall survival, overall cohort</td>
<td>The original figure may be found in the primary trial manuscript

Doki Y, Ajani JA, Kato K, Xu J, Wyrwicz L, Motoyama S, Ogata T, Kawakami H, Hsu CH, Adenis A, El Hajbi F, Di Bartolomeo M, Braghiroli MI, Holtved E, Ostoich SA, Kim HR, Ueno M, Mansoor W, Yang WC, Liu T, Bridgewater J, Makino T, Xynos I, Liu X, Lei M, Kondo K, Patel A, Gricar J, Chau I, Kitagawa Y; CheckMate 648 Trial Investigators. Nivolumab Combination Therapy in Advanced Esophageal Squamous-Cell Carcinoma. N Engl J Med. 2022 Feb 3;386(5):449-462. Doi: 10.1056/NEJMoa2111380. PMID: 35108470.

Figure 1B
Figure 2B</td>
<td>
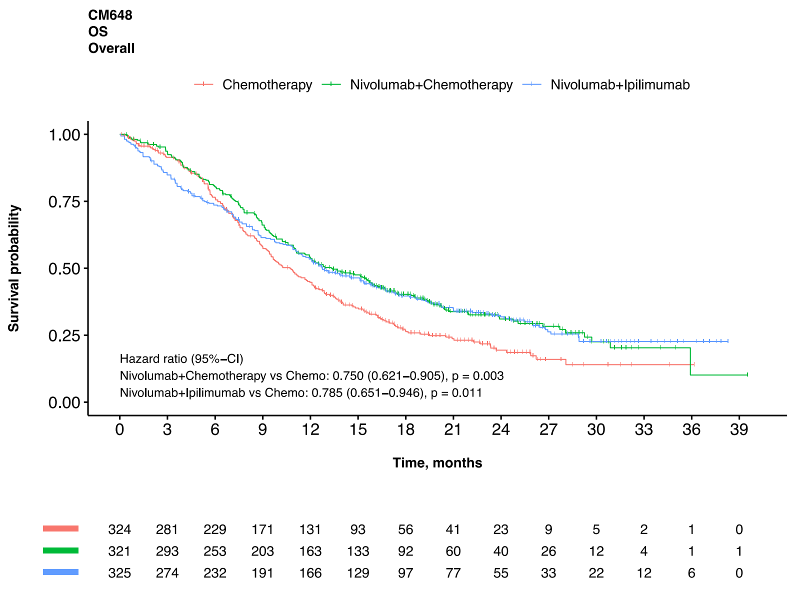

</td>
</tr>
</table>

© 2022 Yap DWT et al. *JAMA Oncology*.

<table>
<tr>
<td>CM648, overall survival, TPS ≥1</td>
<td>The original figure may be found in the primary trial manuscript

Doki Y, Ajani JA, Kato K, Xu J, Wyrwicz L, Motoyama S, Ogata T, Kawakami H, Hsu CH, Adenis A, El Hajbi F, Di Bartolomeo M, Braghiroli MI, Holtved E, Ostoich SA, Kim HR, Ueno M, Mansoor W, Yang WC, Liu T, Bridgewater J, Makino T, Xynos I, Liu X, Lei M, Kondo K, Patel A, Gricar J, Chau I, Kitagawa Y; CheckMate 648 Trial Investigators. Nivolumab Combination Therapy in Advanced Esophageal Squamous-Cell Carcinoma. N Engl J Med. 2022 Feb 3;386(5):449-462. Doi: 10.1056/NEJMoa2111380. PMID: 35108470.

Figure 1A
Figure 2A</td>
<td>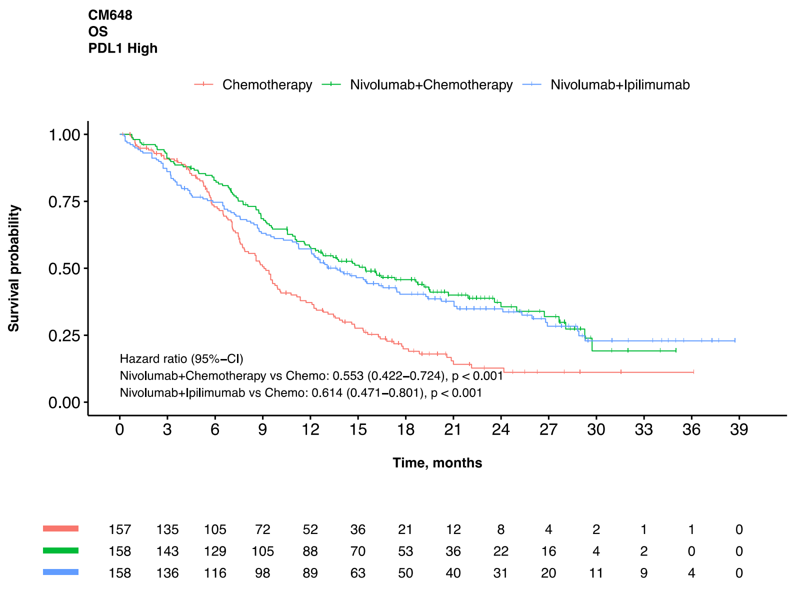
</td>
</tr>
</table>

© 2022 Yap DWT et al. *JAMA Oncology*.

**CM648, Progression Free Survival, Overall cohort**	The original figure may be found in the primary trial manuscript<br><br>Doki Y, Ajani JA, Kato K, Xu J, Wyrwicz L, Motoyama S, Ogata T, Kawakami H, Hsu CH, Adenis A, El Hajbi F, Di Bartolomeo M, Braghiroli MI, Holtved E, Ostoich SA, Kim HR, Ueno M, Mansoor W, Yang WC, Liu T, Bridgewater J, Makino T, Xynos I, Liu X, Lei M, Kondo K, Patel A, Gricar J, Chau I, Kitagawa Y; CheckMate 648 Trial Investigators. Nivolumab Combination Therapy in Advanced Esophageal Squamous-Cell Carcinoma. N Engl J Med. 2022 Feb 3;386(5):449-462. Doi: 10.1056/NEJMoa2111380. PMID: 35108470.<br><br>Figure 1D<br>Figure 2D	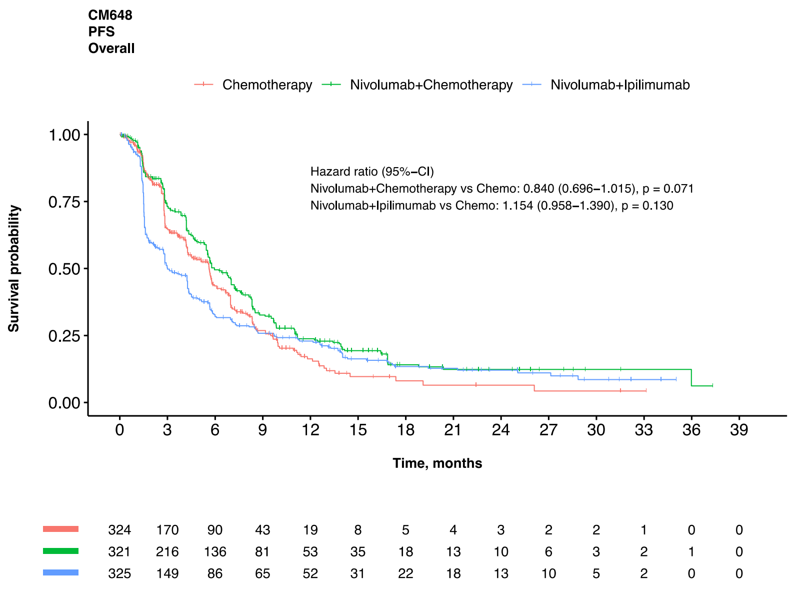

© 2022 Yap DWT et al. *JAMA Oncology*.

<table>
<tr>
<td>CM648, Progression Free Survival, TPS ≥1</td>
<td>The original figure may be found in the primary trial manuscript

Doki Y, Ajani JA, Kato K, Xu J, Wyrwicz L, Motoyama S, Ogata T, Kawakami H, Hsu CH, Adenis A, El Hajbi F, Di Bartolomeo M, Braghiroli MI, Holtved E, Ostoich SA, Kim HR, Ueno M, Mansoor W, Yang WC, Liu T, Bridgewater J, Makino T, Xynos I, Liu X, Lei M, Kondo K, Patel A, Gricar J, Chau I, Kitagawa Y; CheckMate 648 Trial Investigators. Nivolumab Combination Therapy in Advanced Esophageal Squamous-Cell Carcinoma. N Engl J Med. 2022 Feb 3;386(5):449-462. Doi: 10.1056/NEJMoa2111380. PMID: 35108470.

Figure 1C
Figure 2C</td>
<td>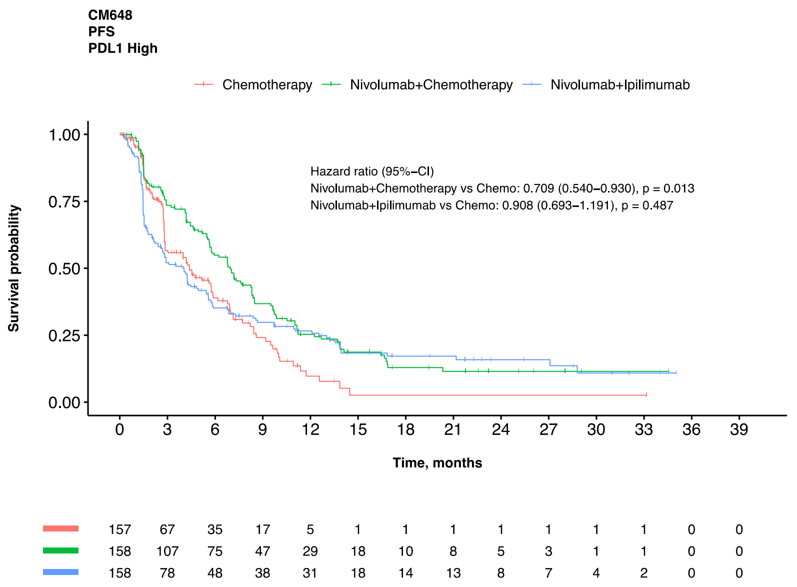
</td>
</tr>
<tr>
<td>CM648, DOR, Overall cohort</td>
<td>The original figure may be found in the primary trial manuscript

Doki Y, Ajani JA, Kato K, Xu J, Wyrwicz L, Motoyama S, Ogata T, Kawakami H, Hsu CH, Adenis A, El Hajbi F, Di Bartolomeo M, Braghiroli MI, Holtved E, Ostoich SA, Kim HR, Ueno M, Mansoor W, Yang WC, Liu T, Bridgewater J, Makino T, Xynos I, Liu X, Lei M, Kondo K, Patel A, Gricar J, Chau I, Kitagawa Y; CheckMate 648 Trial Investigators. Nivolumab Combination Therapy in Advanced Esophageal Squamous-Cell Carcinoma. N Engl J Med. 2022 Feb 3;386(5):449-462. Doi: 10.1056/NEJMoa2111380. PMID: 35108470.

Figure S2B
Figure S2D</td>
<td>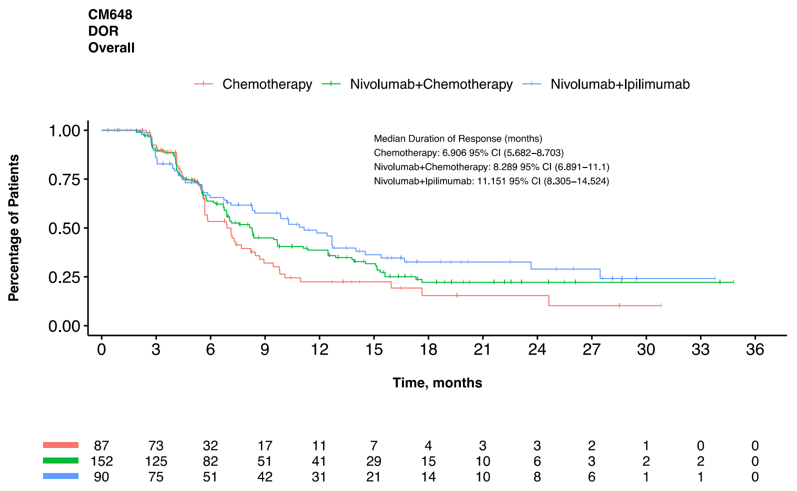
</td>
</tr>
</table>

© 2022 Yap DWT et al. *JAMA Oncology*.

<table>
<tr>
<td>CM648, DOR, TPS ≥1</td>
<td>The original figure may be found in the primary trial manuscript

Doki Y, Ajani JA, Kato K, Xu J, Wyrwicz L, Motoyama S, Ogata T, Kawakami H, Hsu CH, Adenis A, El Hajbi F, Di Bartolomeo M, Braghiroli MI, Holtved E, Ostoich SA, Kim HR, Ueno M, Mansoor W, Yang WC, Liu T, Bridgewater J, Makino T, Xynos I, Liu X, Lei M, Kondo K, Patel A, Gricar J, Chau I, Kitagawa Y; CheckMate 648 Trial Investigators. Nivolumab Combination Therapy in Advanced Esophageal Squamous-Cell Carcinoma. N Engl J Med. 2022 Feb 3;386(5):449-462. Doi: 10.1056/NEJMoa2111380. PMID: 35108470.

Figure S2A
Figure S2C</td>
<td>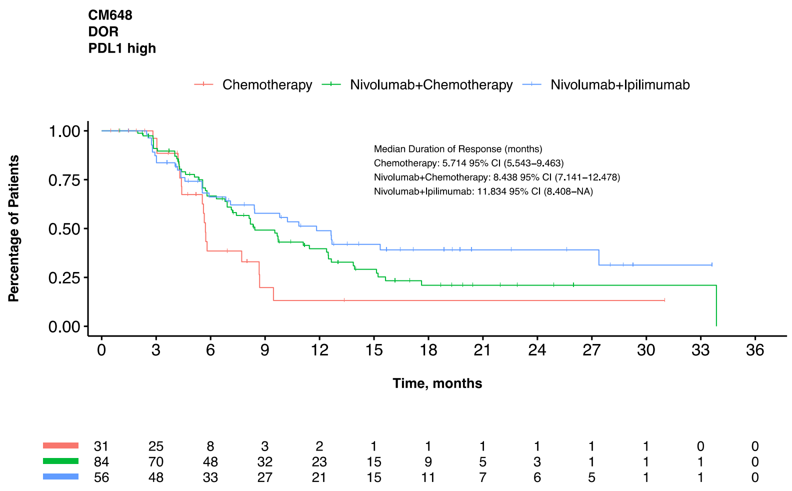
</td>
</tr>
<tr>
<td>ESCORT, overall survival, overall cohort</td>
<td>The original figure may be found in the primary trial manuscript

Huang J, Xu J, Chen Y, Zhuang W, Zhang Y, Chen Z, Chen J, Zhang H, Niu Z, Fan Q, Lin L, Gu K, Liu Y, Ba Y, Miao Z, Jiang X, Zeng M, Chen J, Fu Z, Gan L, Wang J, Zhan X, Liu T, Li Z, Shen L, Shu Y, Zhang T, Yang Q, Zou J; ESCORT Study Group. Camrelizumab versus investigator's choice of chemotherapy as second-line therapy for advanced or metastatic oesophageal squamous cell carcinoma (ESCORT): a multicentre, randomised, open-label, phase 3 study. Lancet Oncol. 2020 Jun;21(6):832-842. doi: 10.1016/S1470-2045(20)30110-8. Epub 2020 May 13. PMID: 32416073.

Figure 2A</td>
<td>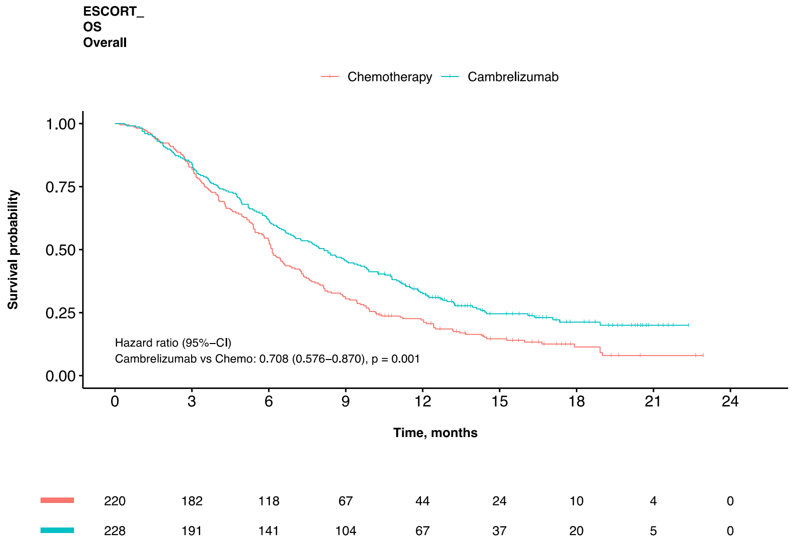
</td>
</tr>
</table>

© 2022 Yap DWT et al. *JAMA Oncology*.

**ESCORT, overall survival, TPS≥1**	The original figure may be found in the primary trial manuscript<br><br>Huang J, Xu J, Chen Y, Zhuang W, Zhang Y, Chen Z, Chen J, Zhang H, Niu Z, Fan Q, Lin L, Gu K, Liu Y, Ba Y, Miao Z, Jiang X, Zeng M, Chen J, Fu Z, Gan L, Wang J, Zhan X, Liu T, Li Z, Shen L, Shu Y, Zhang T, Yang Q, Zou J; ESCORT Study Group. Camrelizumab versus investigator's choice of chemotherapy as second-line therapy for advanced or metastatic oesophageal squamous cell carcinoma (ESCORT): a multicentre, randomised, open-label, phase 3 study. Lancet Oncol. 2020 Jun;21(6):832-842. doi: 10.1016/S1470-2045(20)30110-8. Epub 2020 May 13. PMID: 32416073.<br><br>Figure S3	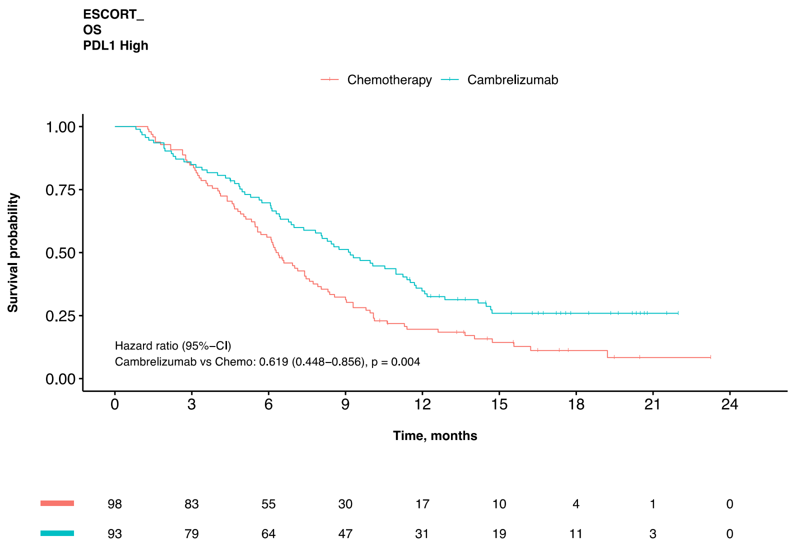
**ESCORT1, overall survival, overall cohort**	The original figure may be found in the primary trial manuscript<br><br>Luo H, Lu J, Bai Y, Mao T, Wang J, Fan Q, Zhang Y, Zhao K, Chen Z, Gao S, Li J, Fu Z, Gu K, Liu Z, Wu L, Zhang X, Feng J, Niu Z, Ba Y, Zhang H, Liu Y, Zhang L, Min X, Huang J, Cheng Y, Wang D, Shen Y, Yang Q, Zou J, Xu RH; ESCORT-1st Investigators. Effect of Camrelizumab vs Placebo Added to Chemotherapy on Survival and Progression-Free Survival in Patients With Advanced or Metastatic Esophageal Squamous Cell Carcinoma: The ESCORT-1st Randomized Clinical Trial. JAMA. 2021 Sep 14;326(10):916-925. doi: 10.1001/jama.2021.12836. PMID: 34519801; PMCID: PMC8441593.<br><br>Figure 2A	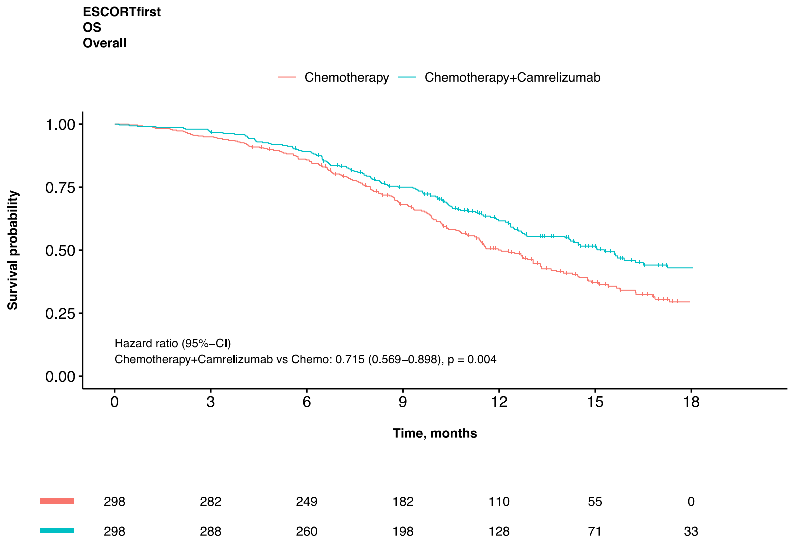

© 2022 Yap DWT et al. *JAMA Oncology*.

**ESCORT1, overall survival, TPS≥1**	The original figure may be found in the primary trial manuscript<br><br>Luo H, Lu J, Bai Y, Mao T, Wang J, Fan Q, Zhang Y, Zhao K, Chen Z, Gao S, Li J, Fu Z, Gu K, Liu Z, Wu L, Zhang X, Feng J, Niu Z, Ba Y, Zhang H, Liu Y, Zhang L, Min X, Huang J, Cheng Y, Wang D, Shen Y, Yang Q, Zou J, Xu RH; ESCORT-1st Investigators. Effect of Camrelizumab vs Placebo Added to Chemotherapy on Survival and Progression-Free Survival in Patients With Advanced or Metastatic Esophageal Squamous Cell Carcinoma: The ESCORT-1st Randomized Clinical Trial. JAMA. 2021 Sep 14;326(10):916-925. doi: 10.1001/jama.2021.12836. PMID: 34519801; PMCID: PMC8441593.<br><br>eFigure 5B	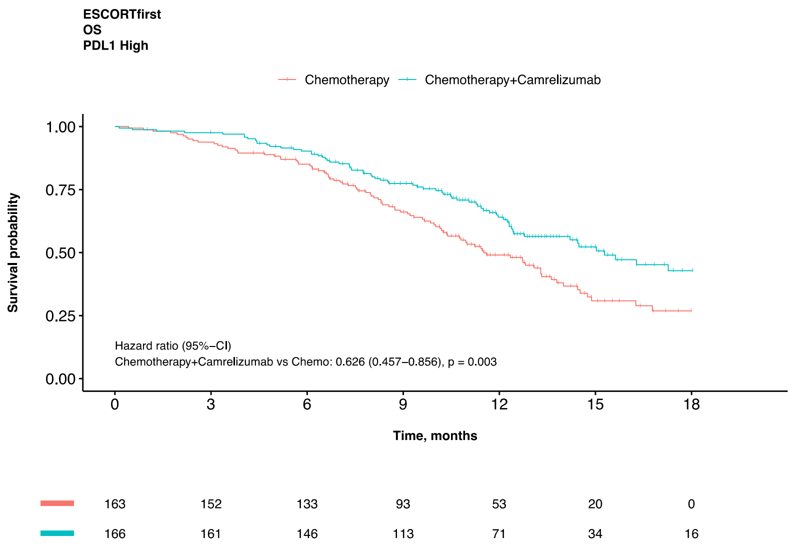
**KN181, overall survival, overall cohort**	The original figure may be found in the primary trial manuscript<br><br>Kojima T, Shah MA, Muro K, Francois E, Adenis A, Hsu CH, Doi T, Moriwaki T, Kim SB, Lee SH, Bennouna J, Kato K, Shen L, Enzinger P, Qin SK, Ferreira P, Chen J, Girotto G, de la Fouchardiere C, Senellart H, Al-Rajabi R, Lordick F, Wang R, Suryawanshi S, Bhagia P, Kang SP, Metges JP; KEYNOTE-181 Investigators. Randomized Phase III KEYNOTE-181 Study of Pembrolizumab Versus Chemotherapy in Advanced Esophageal Cancer. J Clin Oncol. 2020 Dec 10;38(35):4138-4148. doi: 10.1200/JCO.20.01888. Epub 2020 Oct 7. PMID: 33026938.<br><br>Figure 2B	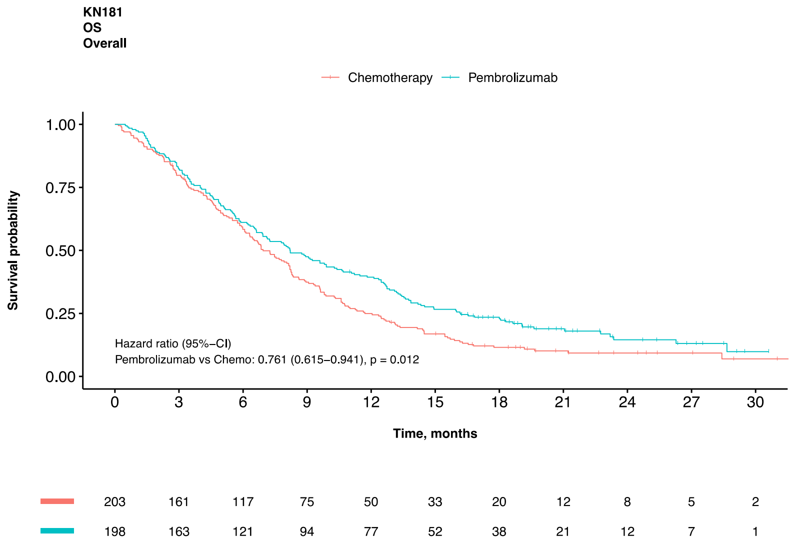

© 2022 Yap DWT et al. *JAMA Oncology*.

**KN181, overall survival, CPS≥10**	The original figure may be found in the primary trial manuscript<br><br>Kojima T, Shah MA, Muro K, Francois E, Adenis A, Hsu CH, Doi T, Moriwaki T, Kim SB, Lee SH, Bennouna J, Kato K, Shen L, Enzinger P, Qin SK, Ferreira P, Chen J, Girotto G, de la Fouchardiere C, Senellart H, Al-Rajabi R, Lordick F, Wang R, Suryawanshi S, Bhagia P, Kang SP, Metges JP; KEYNOTE-181 Investigators. Randomized Phase III KEYNOTE-181 Study of Pembrolizumab Versus Chemotherapy in Advanced Esophageal Cancer. J Clin Oncol. 2020 Dec 10;38(35):4138-4148. doi: 10.1200/JCO.20.01888. Epub 2020 Oct 7. PMID: 33026938.<br><br>Figure S4	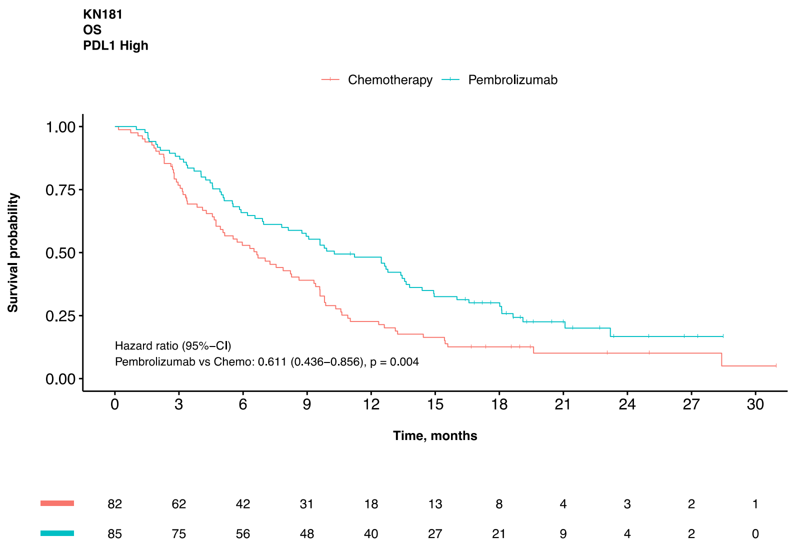
**KN590, overall survival, overall cohort**	The original figure may be found in the primary trial manuscript<br><br>Sun JM, Shen L, Shah MA, Enzinger P, Adenis A, Doi T, Kojima T, Metges JP, Li Z, Kim SB, Cho BC, Mansoor W, Li SH, Sunpaweravong P, Maqueda MA, Goekkurt E, Hara H, Antunes L, Fountzilas C, Tsuji A, Oliden VC, Liu Q, Shah S, Bhagia P, Kato K; KEYNOTE-590 Investigators. Pembrolizumab plus chemotherapy versus chemotherapy alone for first-line treatment of advanced oesophageal cancer (KEYNOTE-590): a randomised, placebo-controlled, phase 3 study. Lancet. 2021 Aug 28;398(10302):759-771. doi: 10.1016/S0140-6736(21)01234-4. Erratum in: Lancet. 2021 Nov 20;398(10314):1874. PMID: 34454674.<br><br>Figure 2B	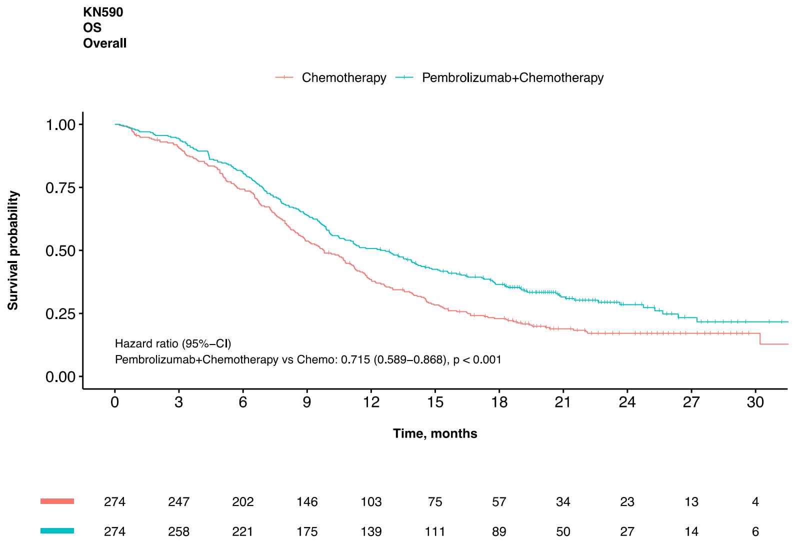

© 2022 Yap DWT et al. *JAMA Oncology*.

<table>
<tr>
<td>KN590, overall survival, CPS≥10</td>
<td>The original figure may be found in the primary trial manuscript

Sun JM, Shen L, Shah MA, Enzinger P, Adenis A, Doi T, Kojima T, Metges JP, Li Z, Kim SB, Cho BC, Mansoor W, Li SH, Sunpaweravong P, Maqueda MA, Goekkurt E, Hara H, Antunes L, Fountzilas C, Tsuji A, Oliden VC, Liu Q, Shah S, Bhagia P, Kato K; KEYNOTE-590 Investigators. Pembrolizumab plus chemotherapy versus chemotherapy alone for first-line treatment of advanced oesophageal cancer (KEYNOTE-590): a randomised, placebo-controlled, phase 3 study. Lancet. 2021 Aug 28;398(10302):759-771. doi: 10.1016/S0140-6736(21)01234-4. Erratum in: Lancet. 2021 Nov 20;398(10314):1874. PMID: 34454674.

Figure 2A</td>
<td>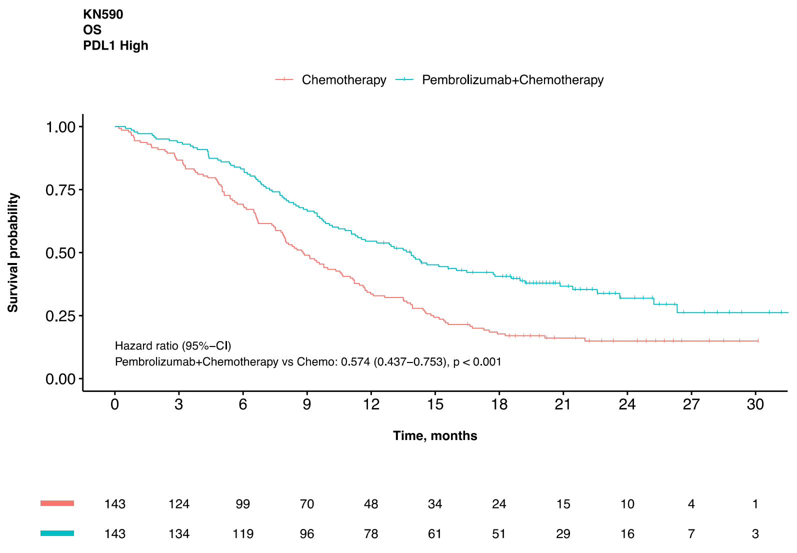
</td>
</tr>
<tr>
<td>ORIENT15, overall survival, overall cohort</td>
<td>The original figure may be found in the primary trial manuscript

Lu Z, Wang J, Shu Y, Liu L, Kong L, Yang L, Wang B, Sun G, Ji Y, Cao G, Liu H, Cui T, Li N, Qiu W, Li G, Hou X, Luo H, Xue L, Zhang Y, Yue W, Liu Z, Wang X, Gao S, Pan Y, Galais MP, Zaanan A, Ma Z, Li H, Wang Y, Shen L; ORIENT-15 study group. Sintilimab versus placebo in combination with chemotherapy as first line treatment for locally advanced or metastatic oesophageal squamous cell carcinoma (ORIENT-15): multicentre, randomised, double blind, phase 3 trial. BMJ. 2022 Apr 19;377:e068714. doi: 10.1136/bmj-2021-068714. PMID: 35440464; PMCID: PMC9016493.

Figure 2 (top)</td>
<td>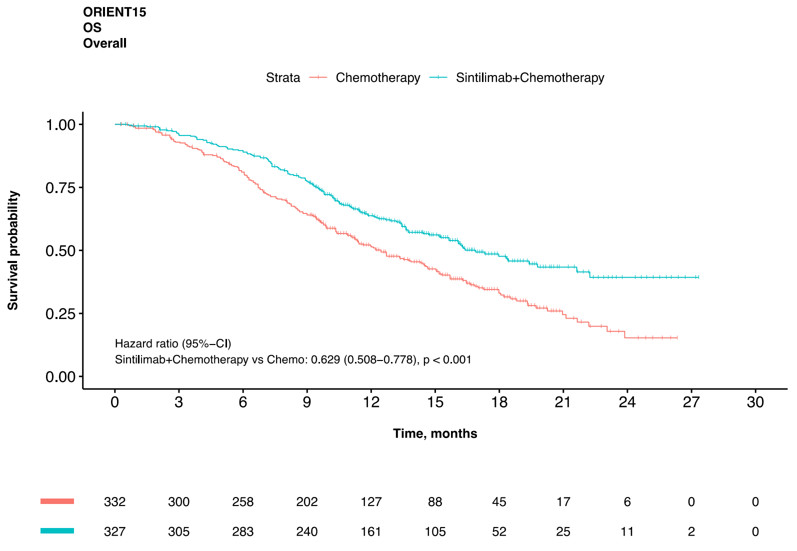
</td>
</tr>
</table>

© 2022 Yap DWT et al. *JAMA Oncology*.

**ORIENT15, overall survival, CPS≥10**	The original figure may be found in the primary trial manuscript<br><br>Lu Z, Wang J, Shu Y, Liu L, Kong L, Yang L, Wang B, Sun G, Ji Y, Cao G, Liu H, Cui T, Li N, Qiu W, Li G, Hou X, Luo H, Xue L, Zhang Y, Yue W, Liu Z, Wang X, Gao S, Pan Y, Galais MP, Zaanan A, Ma Z, Li H, Wang Y, Shen L; ORIENT-15 study group. Sintilimab versus placebo in combination with chemotherapy as first line treatment for locally advanced or metastatic oesophageal squamous cell carcinoma (ORIENT-15): multicentre, randomised, double blind, phase 3 trial. BMJ. 2022 Apr 19;377:e068714. doi: 10.1136/bmj-2021-068714. PMID: 35440464; PMCID: PMC9016493.<br><br>Figure 2 (bottom)	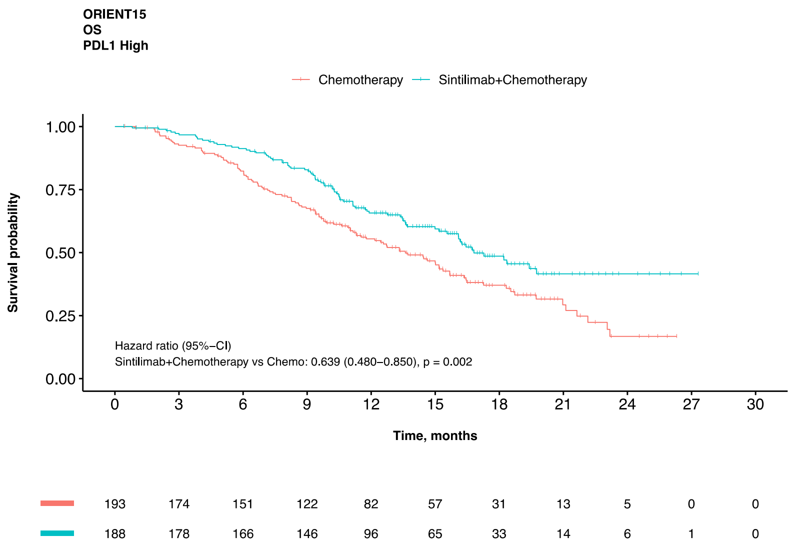
**ORIENT15, Progression Free Survival, Overall cohort**	The original figure may be found in the primary trial manuscript<br><br>Lu Z, Wang J, Shu Y, Liu L, Kong L, Yang L, Wang B, Sun G, Ji Y, Cao G, Liu H, Cui T, Li N, Qiu W, Li G, Hou X, Luo H, Xue L, Zhang Y, Yue W, Liu Z, Wang X, Gao S, Pan Y, Galais MP, Zaanan A, Ma Z, Li H, Wang Y, Shen L; ORIENT-15 study group. Sintilimab versus placebo in combination with chemotherapy as first line treatment for locally advanced or metastatic oesophageal squamous cell carcinoma (ORIENT-15): multicentre, randomised, double blind, phase 3 trial. BMJ. 2022 Apr 19;377:e068714. doi: 10.1136/bmj-2021-068714. PMID: 35440464; PMCID: PMC9016493.<br><br>Figure 4 (top)	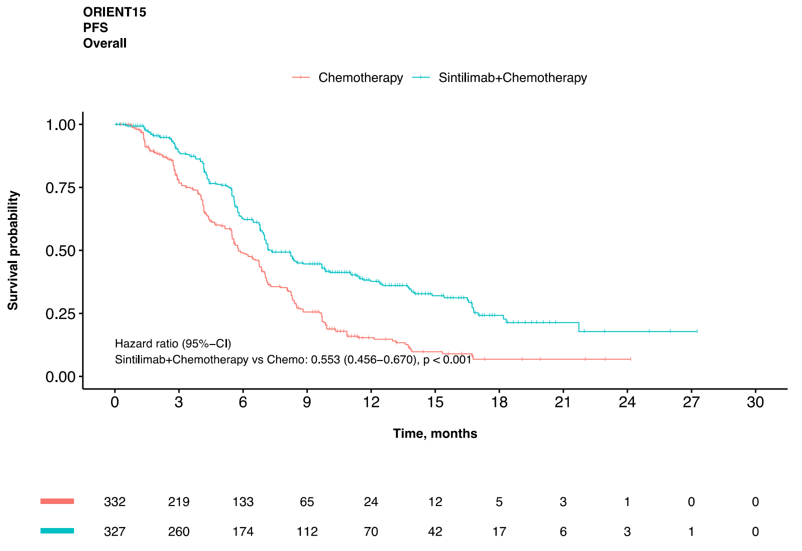

© 2022 Yap DWT et al. *JAMA Oncology*.

**ORIENT15, Progression Free Survival, TPS ≥1**	The original figure may be found in the primary trial manuscript<br><br>Lu Z, Wang J, Shu Y, Liu L, Kong L, Yang L, Wang B, Sun G, Ji Y, Cao G, Liu H, Cui T, Li N, Qiu W, Li G, Hou X, Luo H, Xue L, Zhang Y, Yue W, Liu Z, Wang X, Gao S, Pan Y, Galais MP, Zaanan A, Ma Z, Li H, Wang Y, Shen L; ORIENT-15 study group. Sintilimab versus placebo in combination with chemotherapy as first line treatment for locally advanced or metastatic oesophageal squamous cell carcinoma (ORIENT-15): multicentre, randomised, double blind, phase 3 trial. BMJ. 2022 Apr 19;377:e068714. doi: 10.1136/bmj-2021-068714. PMID: 35440464; PMCID: PMC9016493.<br><br>Figure 4 (bottom)	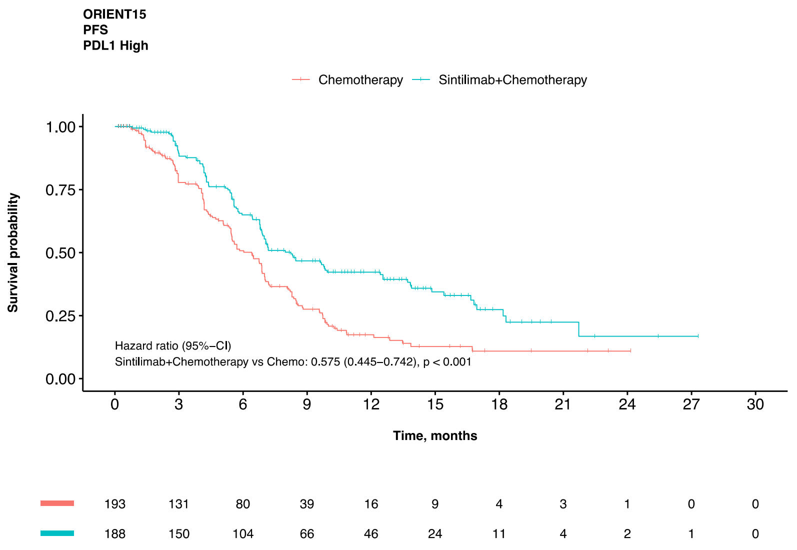
**ORIENT15, DOR, Overall cohort**	The original figure may be found in the primary trial manuscript<br><br>Lu Z, Wang J, Shu Y, Liu L, Kong L, Yang L, Wang B, Sun G, Ji Y, Cao G, Liu H, Cui T, Li N, Qiu W, Li G, Hou X, Luo H, Xue L, Zhang Y, Yue W, Liu Z, Wang X, Gao S, Pan Y, Galais MP, Zaanan A, Ma Z, Li H, Wang Y, Shen L; ORIENT-15 study group. Sintilimab versus placebo in combination with chemotherapy as first line treatment for locally advanced or metastatic oesophageal squamous cell carcinoma (ORIENT-15): multicentre, randomised, double blind, phase 3 trial. BMJ. 2022 Apr 19;377:e068714. doi: 10.1136/bmj-2021-068714. PMID: 35440464; PMCID: PMC9016493.<br><br>Figure S2	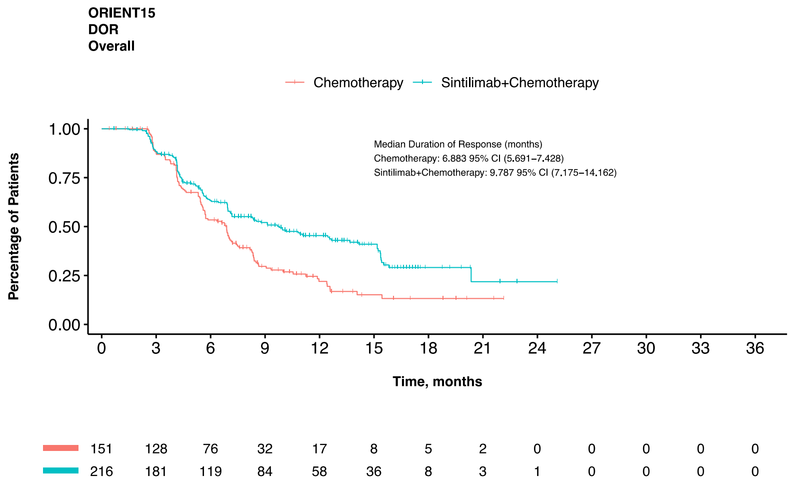

© 2022 Yap DWT et al. *JAMA Oncology*.

<table>
<tr>
<td>ORIENT15,
DOR, TPS ≥1</td>
<td>The original figure may be found in the primary trial manuscript

Lu Z, Wang J, Shu Y, Liu L, Kong L, Yang L, Wang B, Sun G, Ji Y, Cao G, Liu H, Cui T, Li N, Qiu W, Li G, Hou X, Luo H, Xue L, Zhang Y, Yue W, Liu Z, Wang X, Gao S, Pan Y, Galais MP, Zaanan A, Ma Z, Li H, Wang Y, Shen L; ORIENT-15 study group. Sintilimab versus placebo in combination with chemotherapy as first line treatment for locally advanced or metastatic oesophageal squamous cell carcinoma (ORIENT-15): multicentre, randomised, double blind, phase 3 trial. BMJ. 2022 Apr 19;377:e068714. doi: 10.1136/bmj-2021-068714. PMID: 35440464; PMCID: PMC9016493.

Figure S3</td>
<td>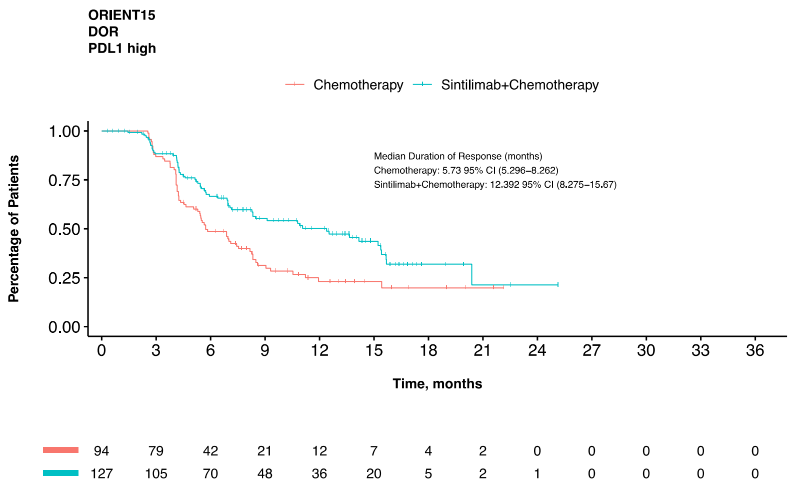
</td>
</tr>
<tr>
<td>RATIONALE30
2,
overall
survival,
overall cohort</td>
<td>The original figure may be found in the primary trial manuscript

Shen L, Kato K, Kim SB, Ajani JA, Zhao K, He Z, Yu X, Shu Y, Luo Q, Wang J, Chen Z, Niu Z, Zhang L, Yi T, Sun JM, Chen J, Yu G, Lin CY, Hara H, Bi Q, Satoh T, Pazo-Cid R, Arkenau HT, Borg C, Lordick F, Li L, Ding N, Tao A, Shi J, Van Cutsem E; RATIONALE-302 Investigators. Tislelizumab Versus Chemotherapy as Second-Line Treatment for Advanced or Metastatic Esophageal Squamous Cell Carcinoma (RATIONALE-302): A Randomized Phase III Study. J Clin Oncol. 2022 Sep 10;40(26):3065-3076. doi: 10.1200/JCO.21.01926. Epub 2022 Apr 20. PMID: 35442766; PMCID: PMC9462531.

Figure 2A</td>
<td>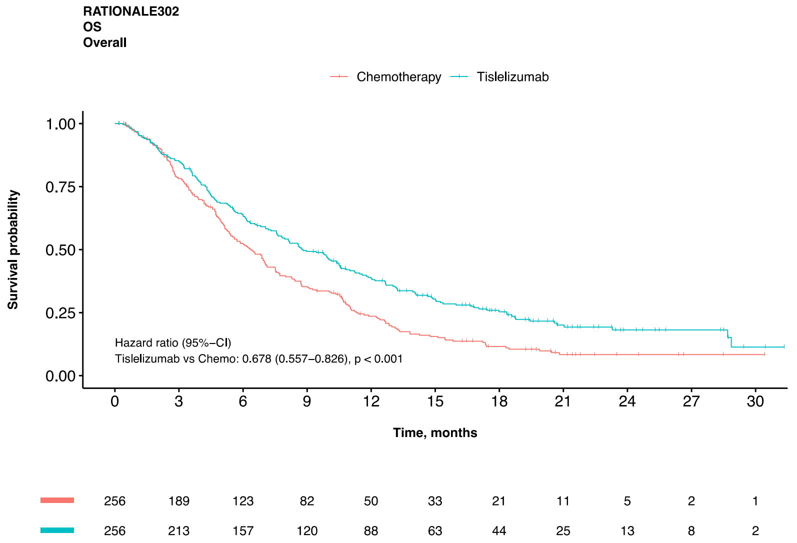
</td>
</tr>
</table>

© 2022 Yap DWT et al. *JAMA Oncology*.

<table>
<tr>
<td>RATIONALE302, overall survival, TAP≥10</td>
<td>The original figure may be found in the primary trial manuscript

Shen L, Kato K, Kim SB, Ajani JA, Zhao K, He Z, Yu X, Shu Y, Luo Q, Wang J, Chen Z, Niu Z, Zhang L, Yi T, Sun JM, Chen J, Yu G, Lin CY, Hara H, Bi Q, Satoh T, Pazo-Cid R, Arkenau HT, Borg C, Lordick F, Li L, Ding N, Tao A, Shi J, Van Cutsem E; RATIONALE-302 Investigators. Tislelizumab Versus Chemotherapy as Second-Line Treatment for Advanced or Metastatic Esophageal Squamous Cell Carcinoma (RATIONALE-302): A Randomized Phase III Study. J Clin Oncol. 2022 Sep 10;40(26):3065-3076. doi: 10.1200/JCO.21.01926. Epub 2022 Apr 20. PMID: 35442766; PMCID: PMC9462531.

Figure 2B</td>
<td>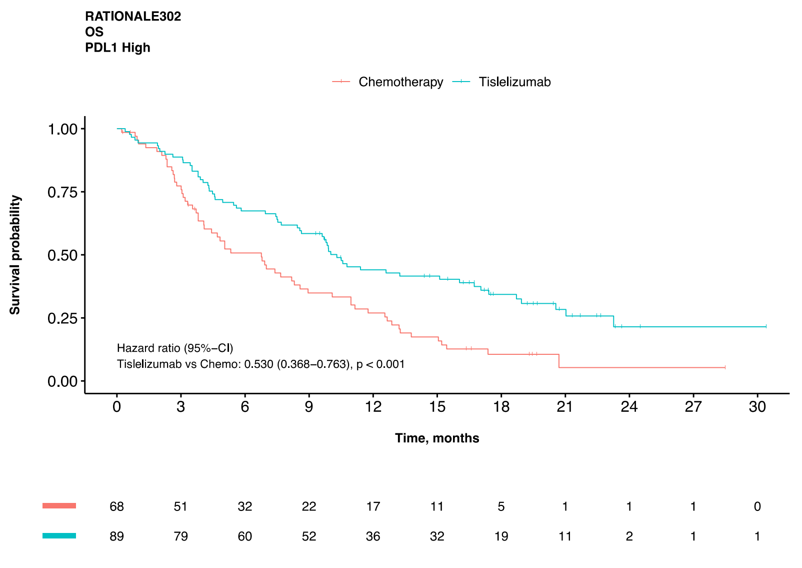
</td>
</tr>
</table>

Abbreviations: CPS, combined positive score; TPS, tumor proportion score; Chemo, chemotherapy; Immuno, immunotherapy; Nivo, nivolumab; Ipi, ipilimumab; Camre, camrelizumab; Pembro, pembrolizumab; Sinti, sintilimab; Tisle, Tislelizumab; HR, hazard ratio; CI, confidence interval; PD-L1, programmed death ligand 1

© 2022 Yap DWT et al. *JAMA Oncology*.

eFigure 4. Example Comparisons of KMSubtraction Outcomes With Reported HRs for PD-L1–Low Subgroups

Trial, comparison	PD-L1 expression subgroup	Outcome	Reported		KMSubtraction with bipartite matching
			Hazard ratio (95%-CI)	p-value	Curve, hazard ratio (95%-CI)
ORIENT15 *(Esophageal squamous cell carcinoma)*					
Sintilimab + Chemothrapy (139) vs Chemotherapy (139)	CPS < 10	OS	0.62 (0.45-0.85)	NR	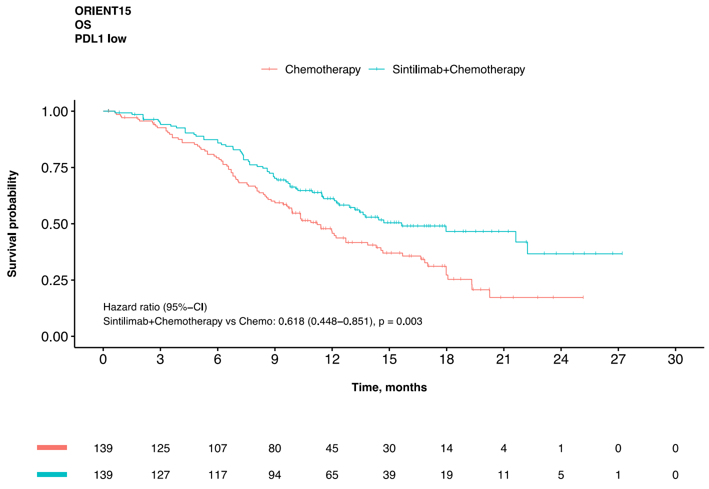

© 2022 Yap DWT et al. *JAMA Oncology*.

Checkmate-648 (Esophageal squamous cell carcinoma)				
Nivo+Chemo vs Chemo	TPS <1	OS	0.98 (0.76-1.28)	NR
Nivo+Ipi vs Chemo	TPS <1	OS	0.96 (0.74-1.25)	NR

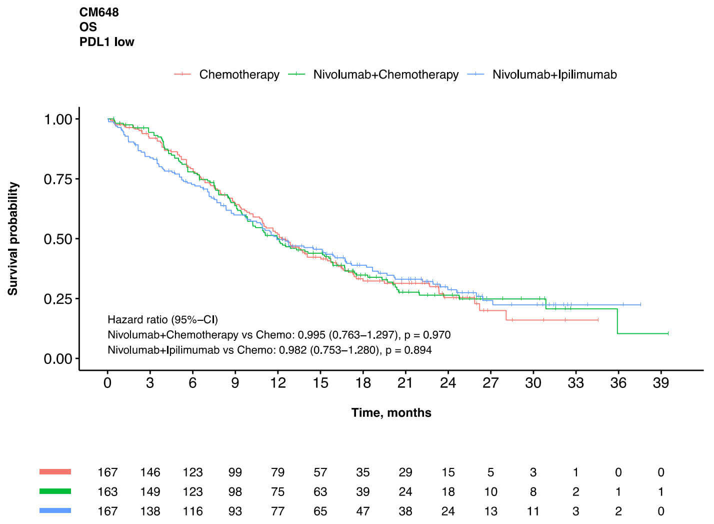

KN590				
Pembro + Chemo vs Chemo	CPS < 10	OS	0.99 (0.74-1.32)	HR

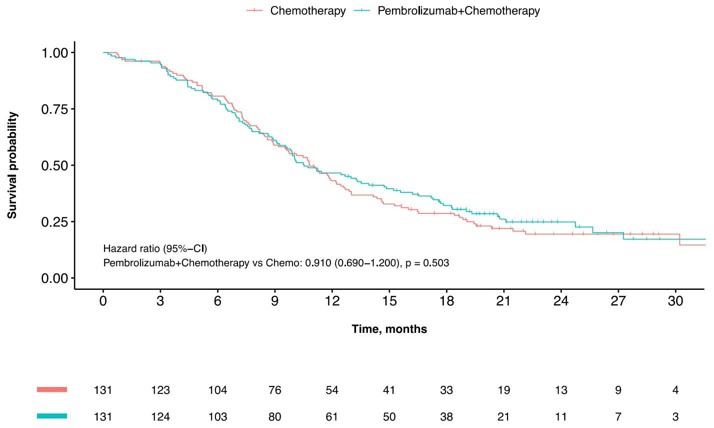

ESCORT

© 2022 Yap DWT et al. *JAMA Oncology*.

Camre vs Chemo	TPS <1	OS	0.82 (0.62-1.09)	NR	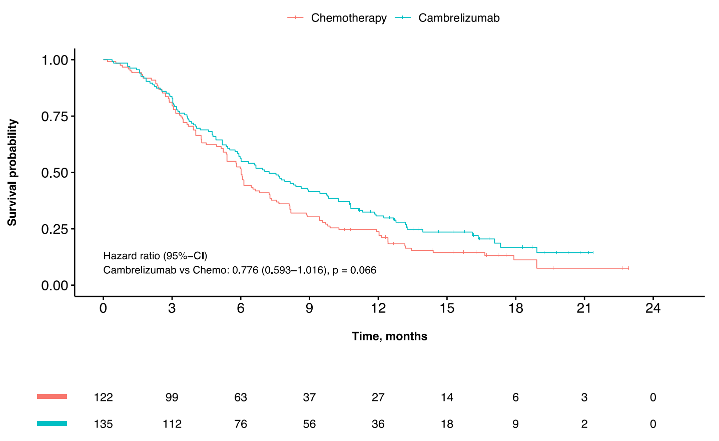
KN181					
Pembro vs Chemo	CPS <10	OS	0.88 (0.66-1.16)		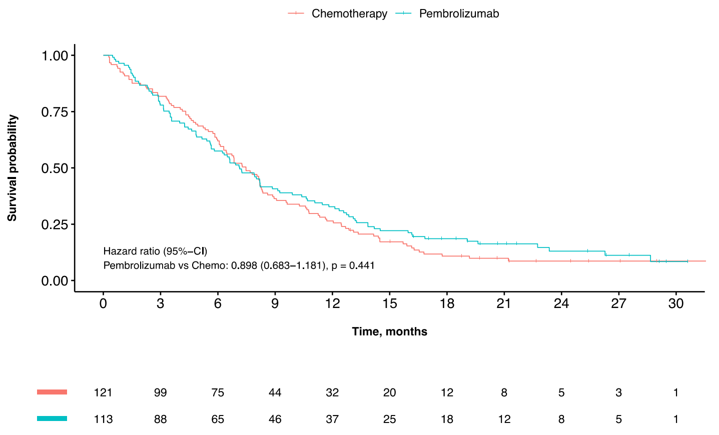

Abbreviations: Sinti, Sintilimab; Nivo, nivolumab; Ipi, ipilimumab; Pembro, Pembrolizumab; Chemo, chemotherapy; CI, confidence interval; PD-L1, programmed death ligand 1 ESCC, esophageal squamous cell carcinoma; NR, not reported; TPS, tumor proportion score.

© 2022 Yap DWT et al. *JAMA Oncology*.

eFigure 5. Evaluation of KMSubtraction Bipartite Matching

**CM648 DOR TPS ≥1% (Chemo)**	
**Empirical cumulative distribution between follow-up time of matched pairs and Bland-Altman plots to explore discrepancies between matched pairs**	**Kaplan Meier Curves and Cox-proportional hazard’s model comparing matched pairs**

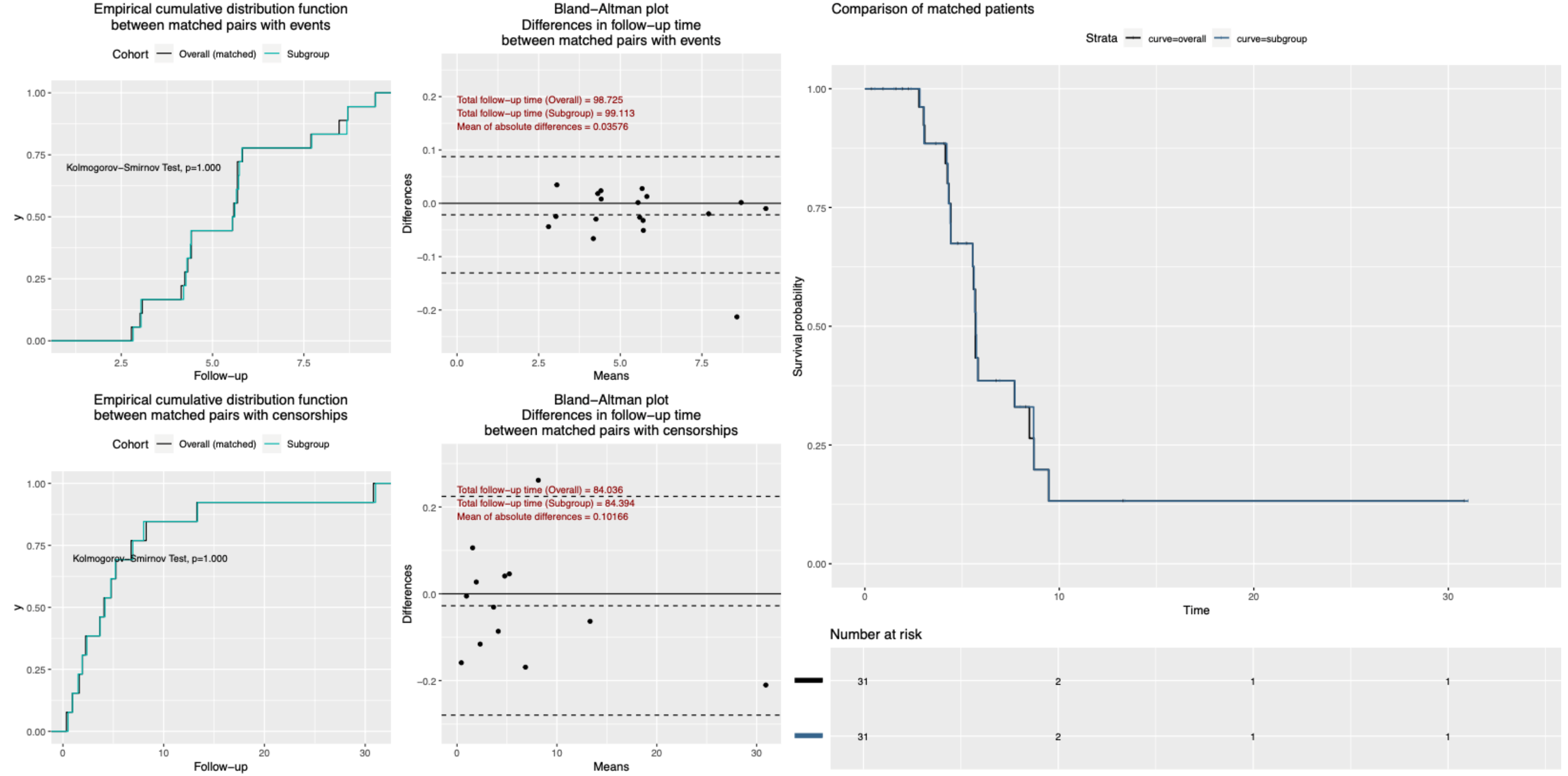

© 2022 Yap DWT et al. *JAMA Oncology*.

CM648 DOR TPS ≥1% (Nivo + Ipi)	
**Empirical cumulative distribution between follow-up time of matched pairs and Bland-Altman plots to explore discrepancies between matched pairs**	**Kaplan Meier Curves and Cox-proportional hazard’s model comparing matched pairs**
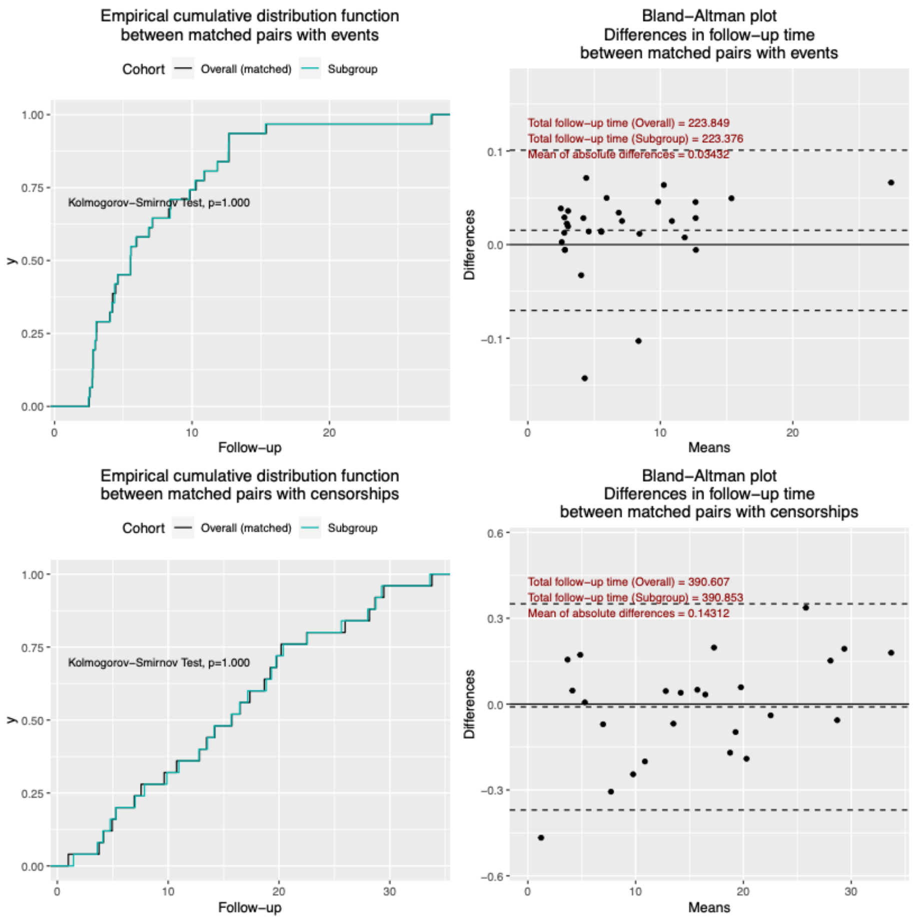	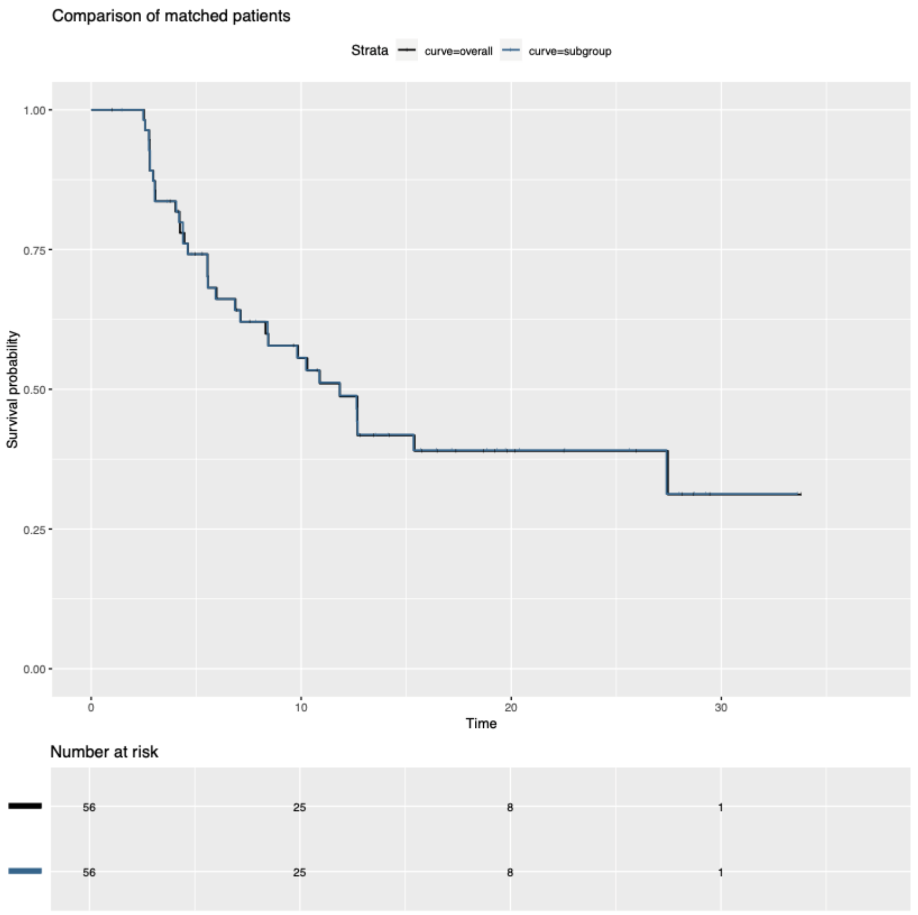

© 2022 Yap DWT et al. *JAMA Oncology*.

CM648 DOR TPS ≥1% (Nivo + Chemo)

Empirical cumulative distribution between follow-up time of matched pairs and Bland-Altman plots to explore discrepancies between matched pairs	Kaplan Meier Curves and Cox-proportional hazard's model comparing matched pairs
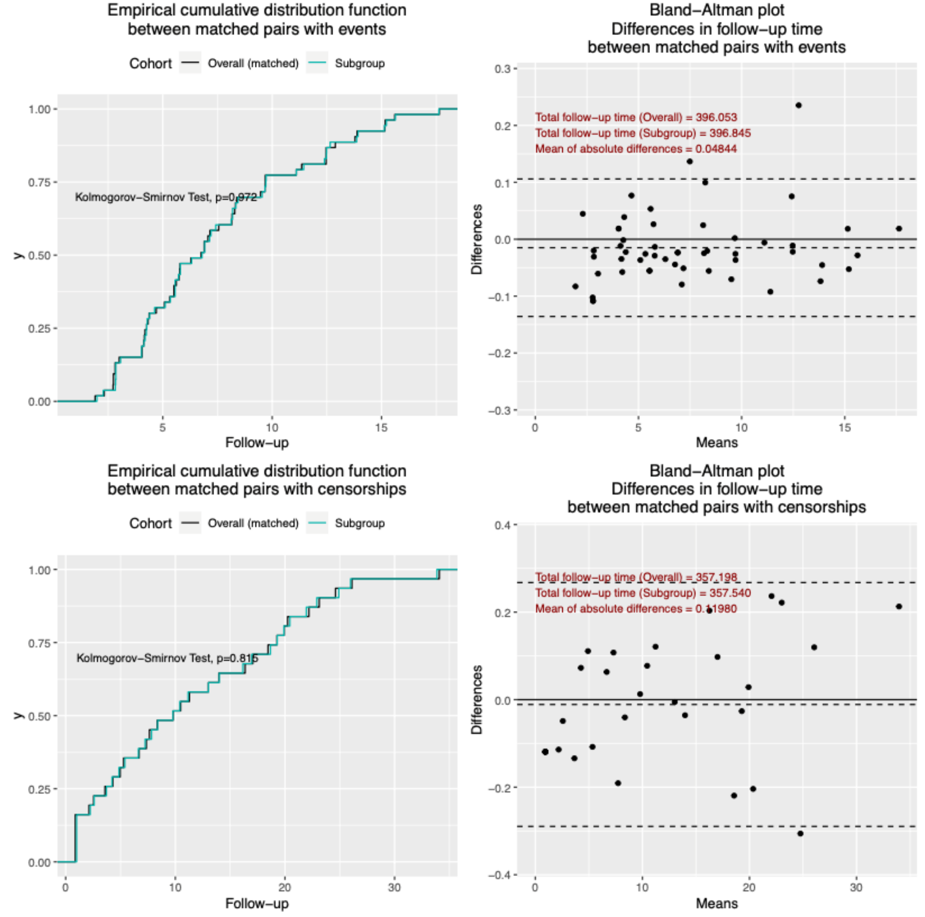	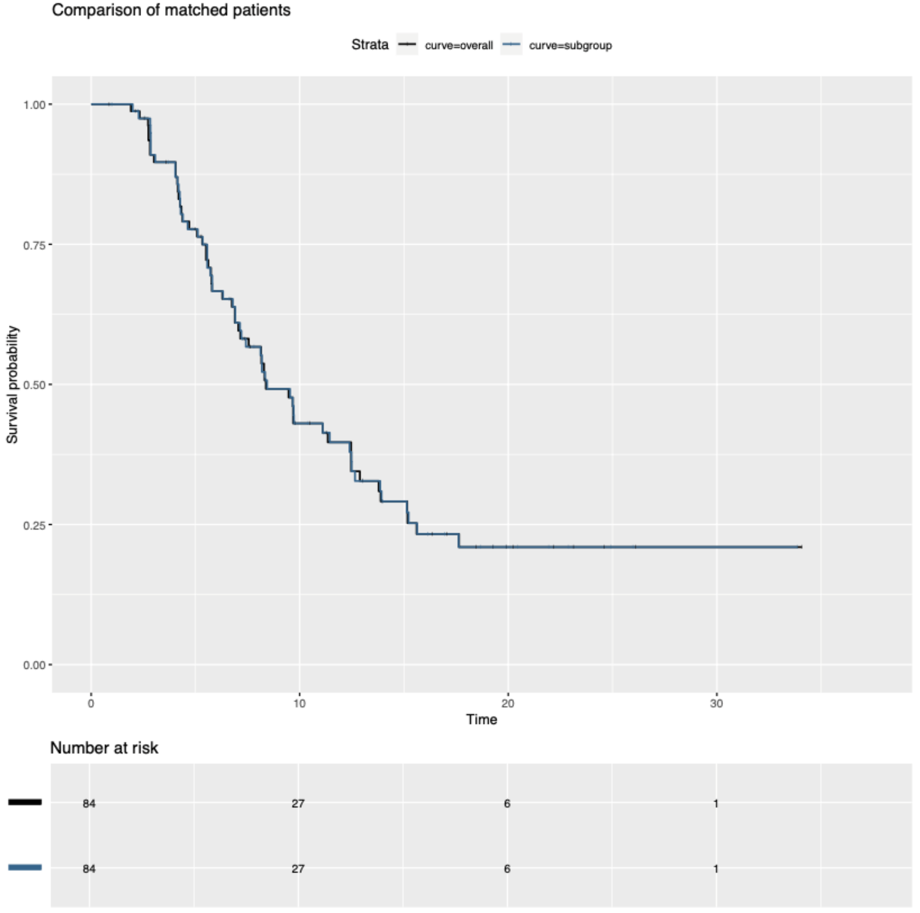

© 2022 Yap DWT et al. *JAMA Oncology*.

CM648 OS TPS ≥1% (Chemo)

Empirical cumulative distribution between follow-up time of matched pairs and Bland-Altman plots to explore discrepancies between matched pairs	Kaplan Meier Curves and Cox-proportional hazard’s model comparing matched pairs
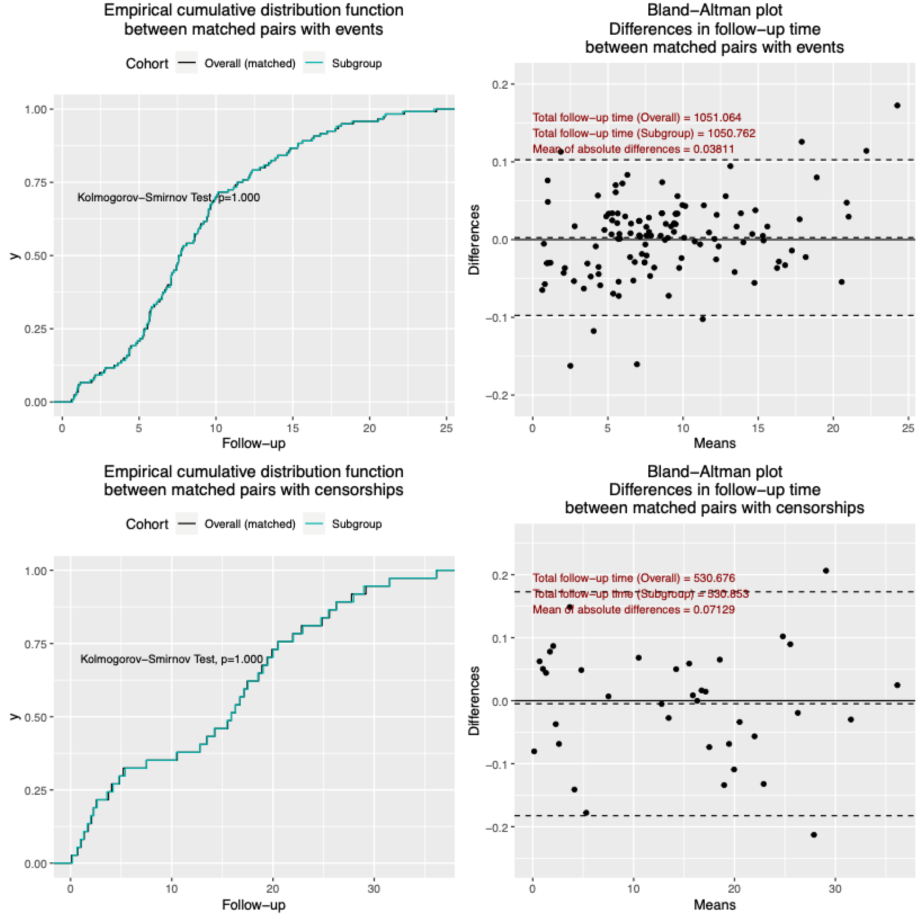	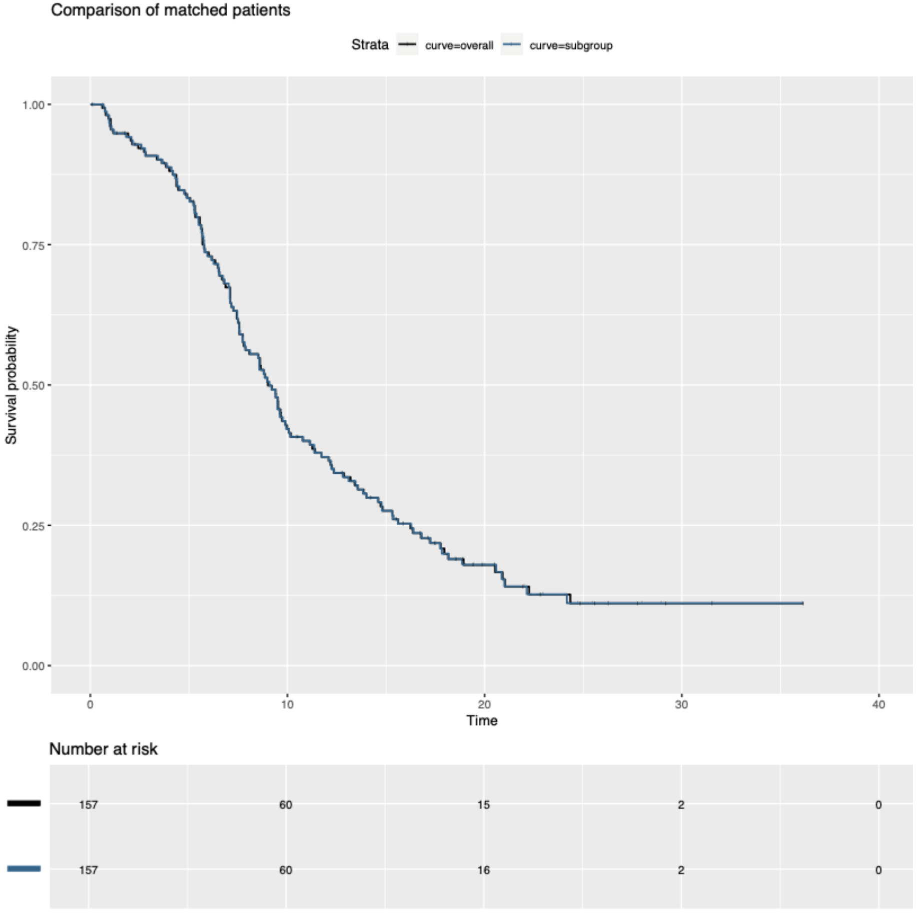

© 2022 Yap DWT et al. *JAMA Oncology*.

CM648 OS TPS ≥1% (Nivo + Ipi)	
**Empirical cumulative distribution between follow-up time of matched pairs and Bland-Altman plots to explore discrepancies between matched pairs**	**Kaplan Meier Curves and Cox-proportional hazard's model comparing matched pairs**

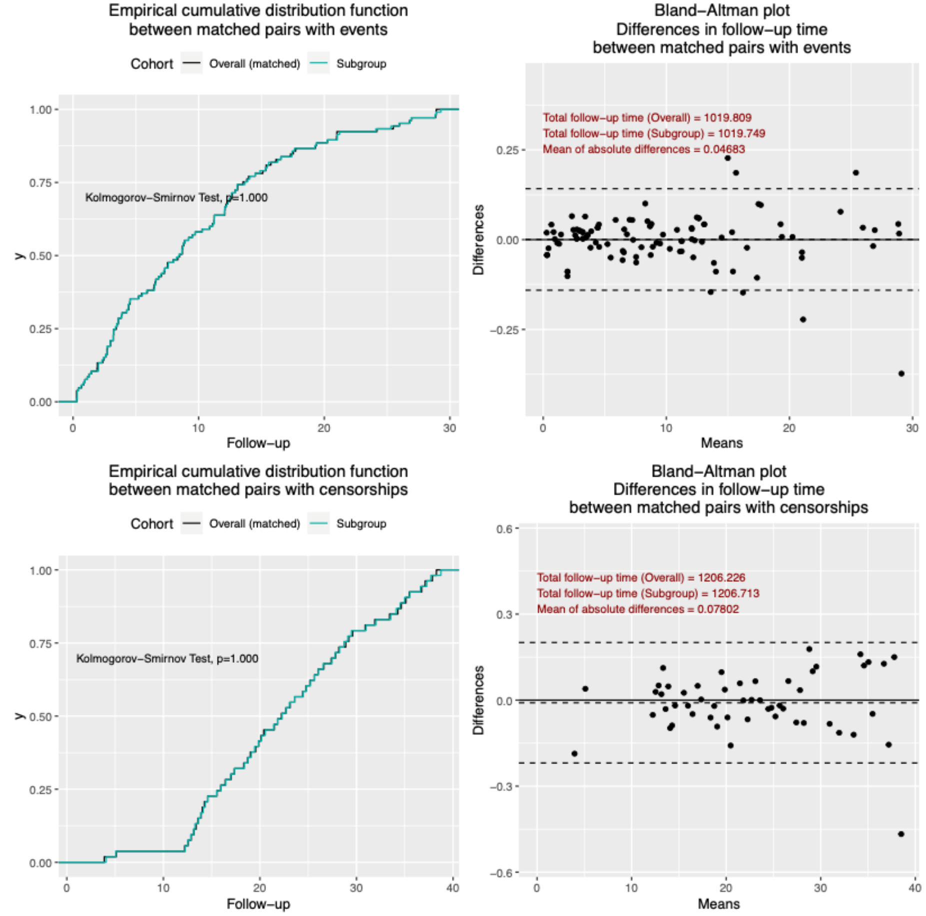

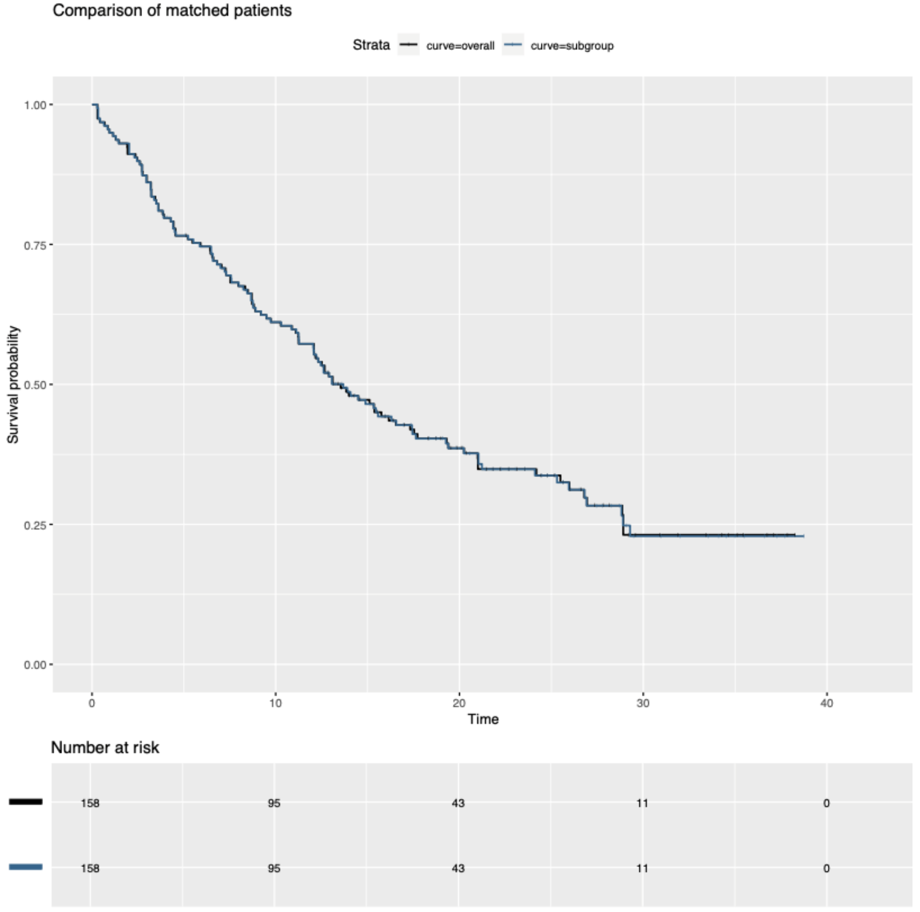

© 2022 Yap DWT et al. *JAMA Oncology*.

CM648 OS TPS ≥1% (Nivo + Chemo)

Empirical cumulative distribution between follow-up time of matched pairs and Bland-Altman plots to explore discrepancies between matched pairs	Kaplan Meier Curves and Cox-proportional hazard's model comparing matched pairs
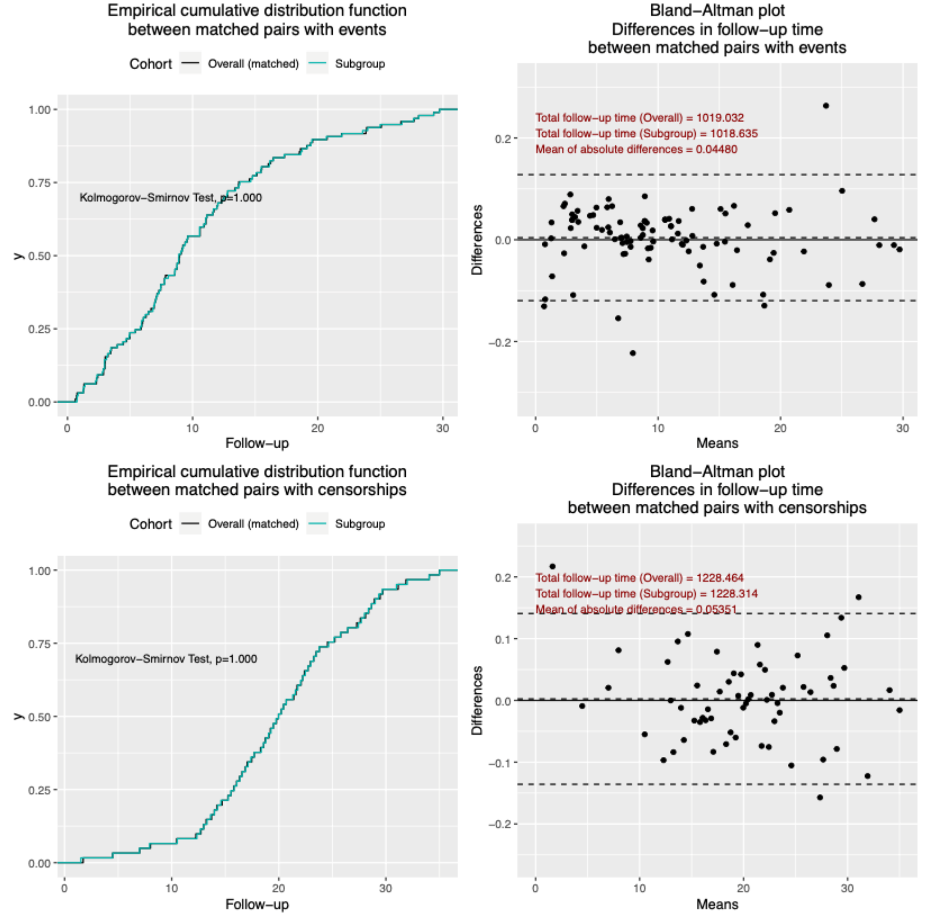	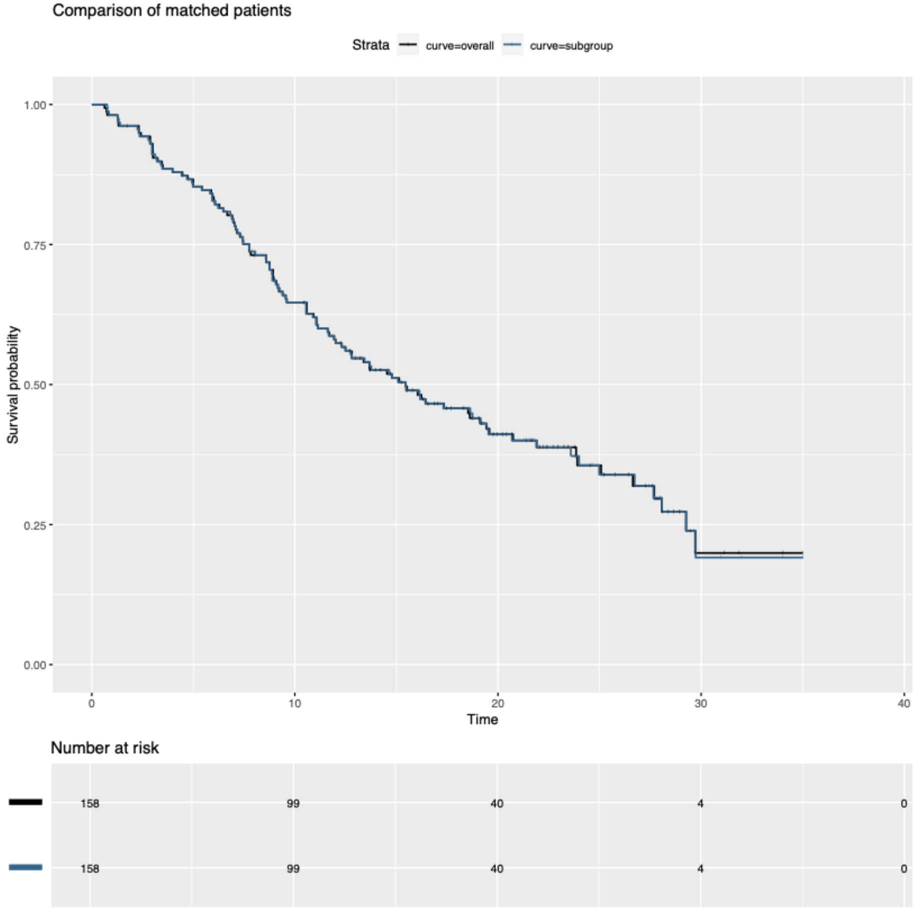

© 2022 Yap DWT et al. *JAMA Oncology*.

CM648 PFS TPS ≥1% (Chemo)

Empirical cumulative distribution between follow-up time of matched pairs and Bland-Altman plots to explore discrepancies between matched pairs	Kaplan Meier Curves and Cox-proportional hazard’s model comparing matched pairs
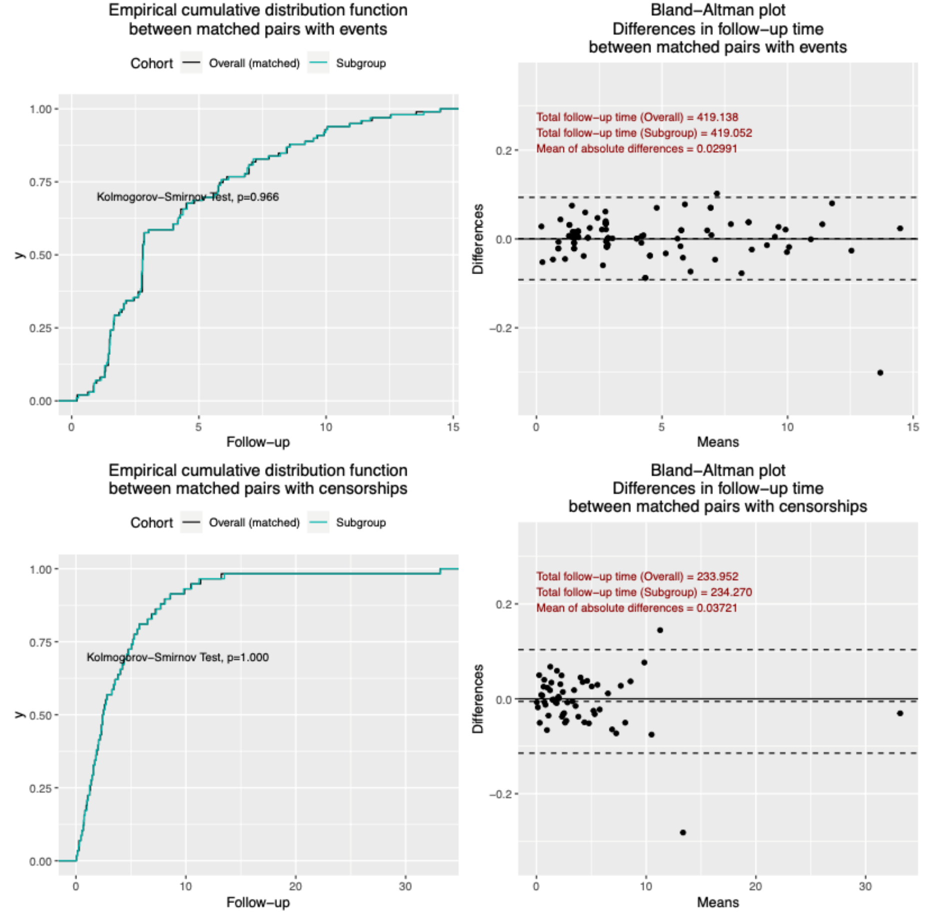	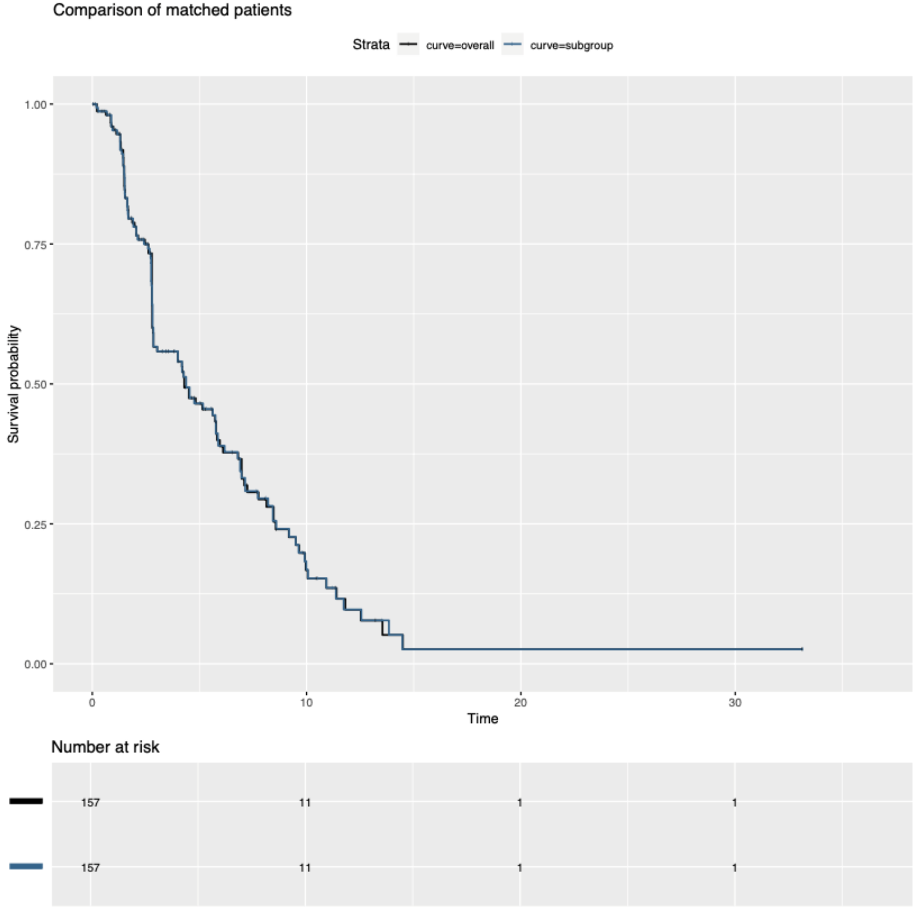

© 2022 Yap DWT et al. *JAMA Oncology*.

CM648 PFS TPS ≥1% (Nivo + Ipi)

Empirical cumulative distribution between follow-up time of matched pairs and Bland-Altman plots to explore discrepancies between matched pairs

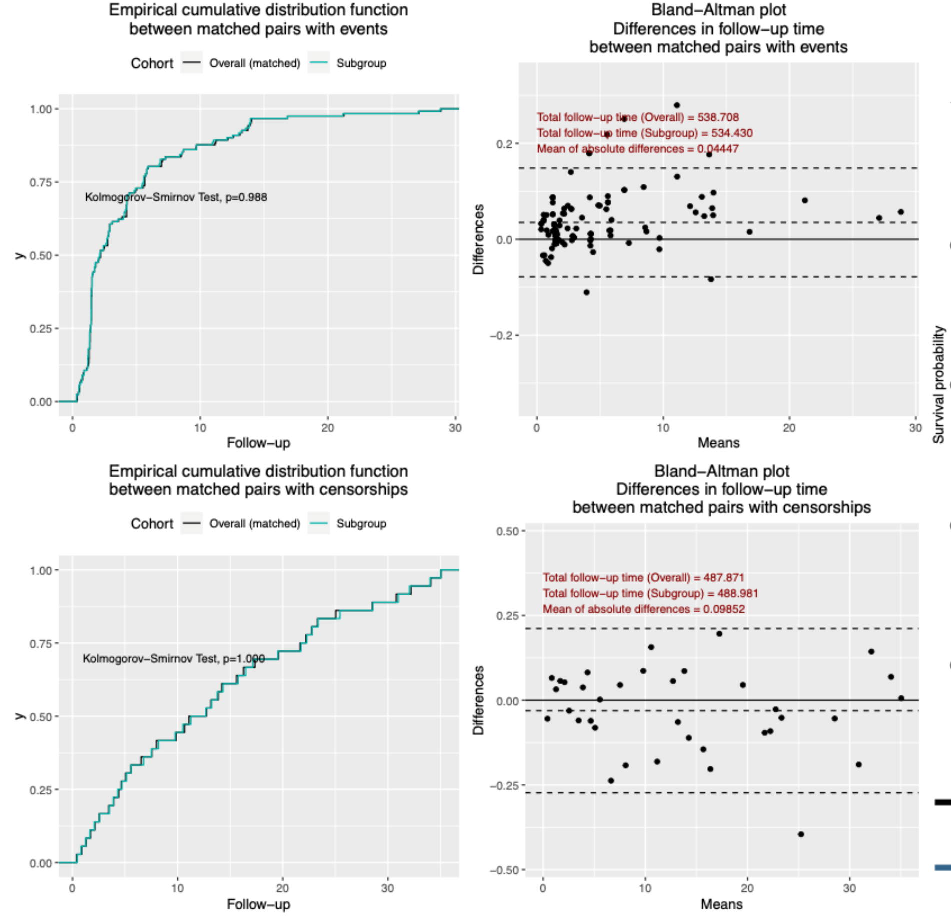

Kaplan Meier Curves and Cox-proportional hazard's model comparing matched pairs

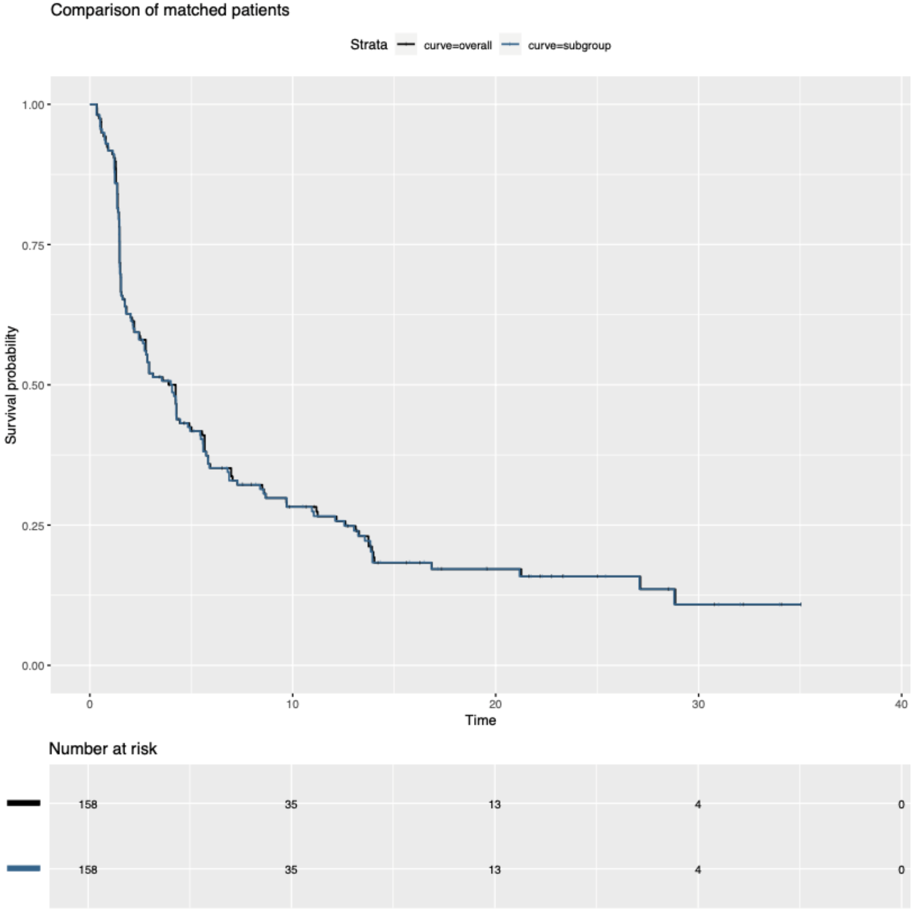

© 2022 Yap DWT et al. *JAMA Oncology*.

CM648 PFS TPS ≥1% (Nivo + Chemo)

Empirical cumulative distribution between follow-up time of matched pairs and Bland-Altman plots to explore discrepancies between matched pairs	Kaplan Meier Curves and Cox-proportional hazard's model comparing matched pairs

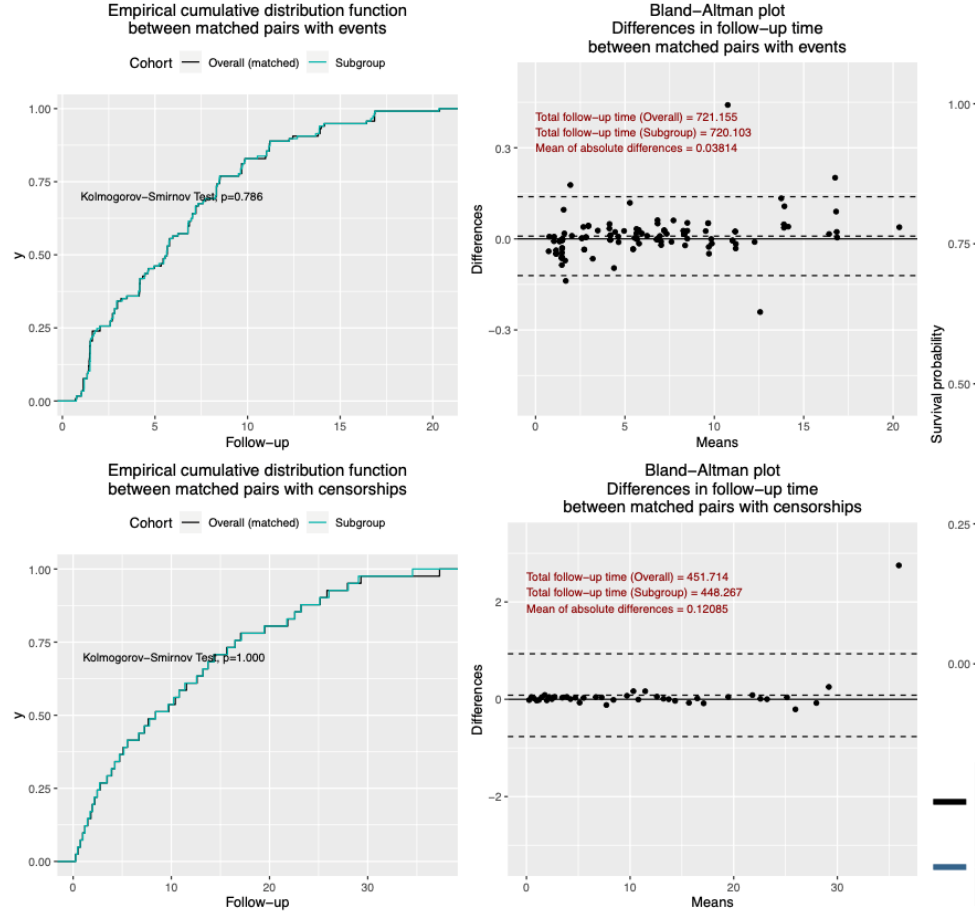

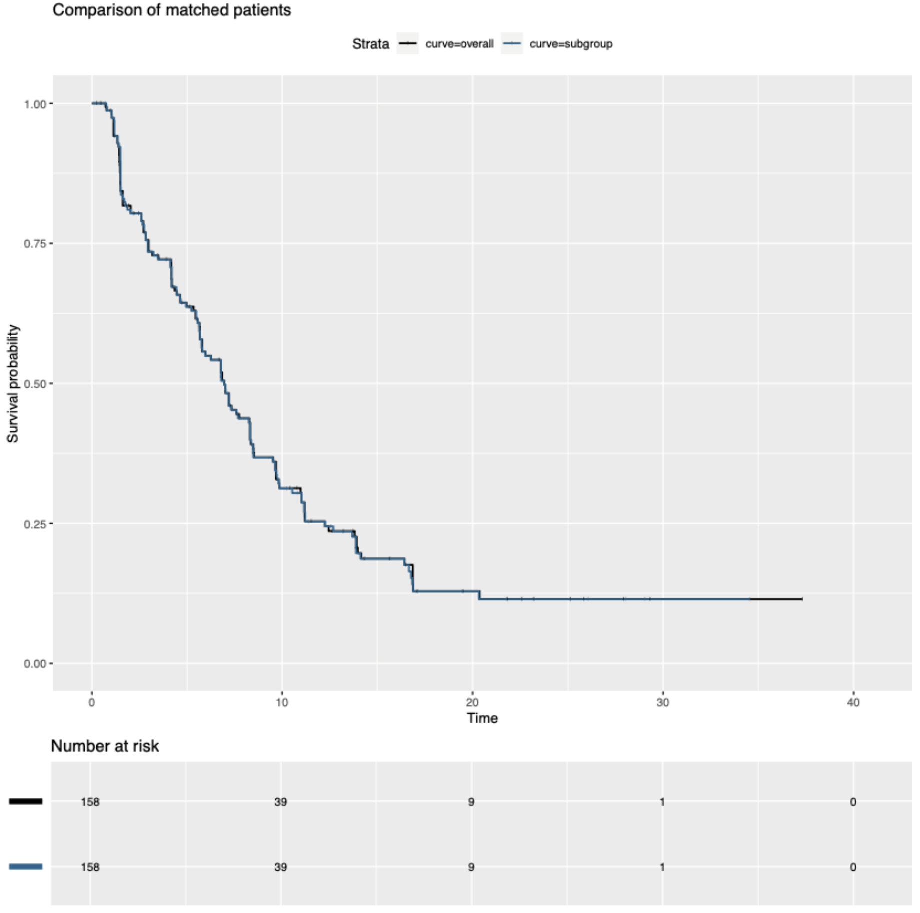

© 2022 Yap DWT et al. *JAMA Oncology*.

ESCORT1st OS TPS ≥1% (Chemo)

Empirical cumulative distribution between follow-up time of matched pairs and Bland-Altman plots to explore discrepancies between matched pairs	Kaplan Meier Curves and Cox-proportional hazard’s model comparing matched pairs
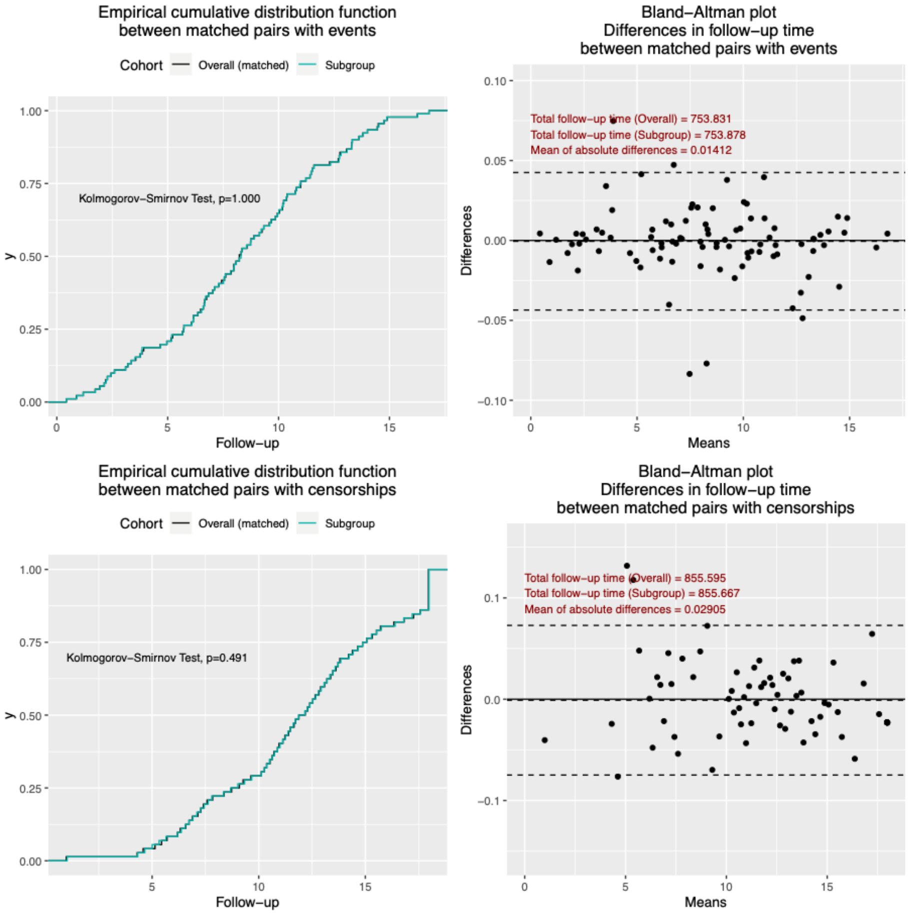	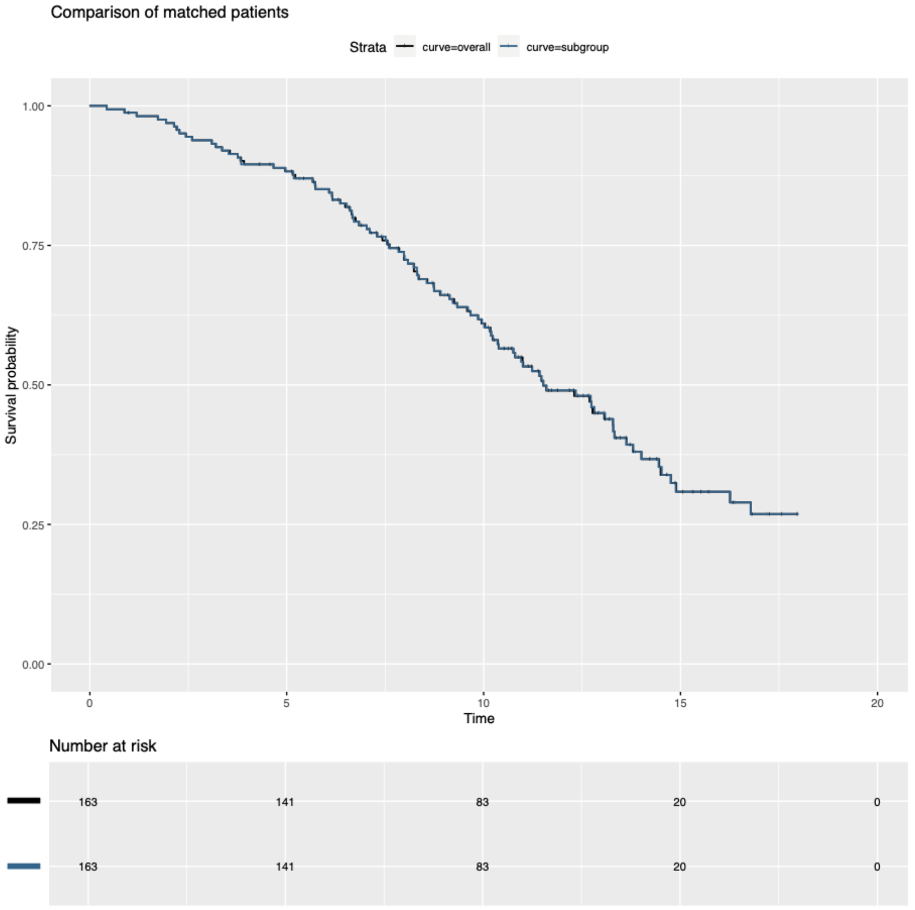

© 2022 Yap DWT et al. *JAMA Oncology*.

ESCORT1st OS TPS ≥1% (Camre + Chemo)	
**Empirical cumulative distribution between follow-up time of matched pairs and Bland-Altman plots to explore discrepancies between matched pairs**	**Kaplan Meier Curves and Cox-proportional hazard’s model comparing matched pairs**

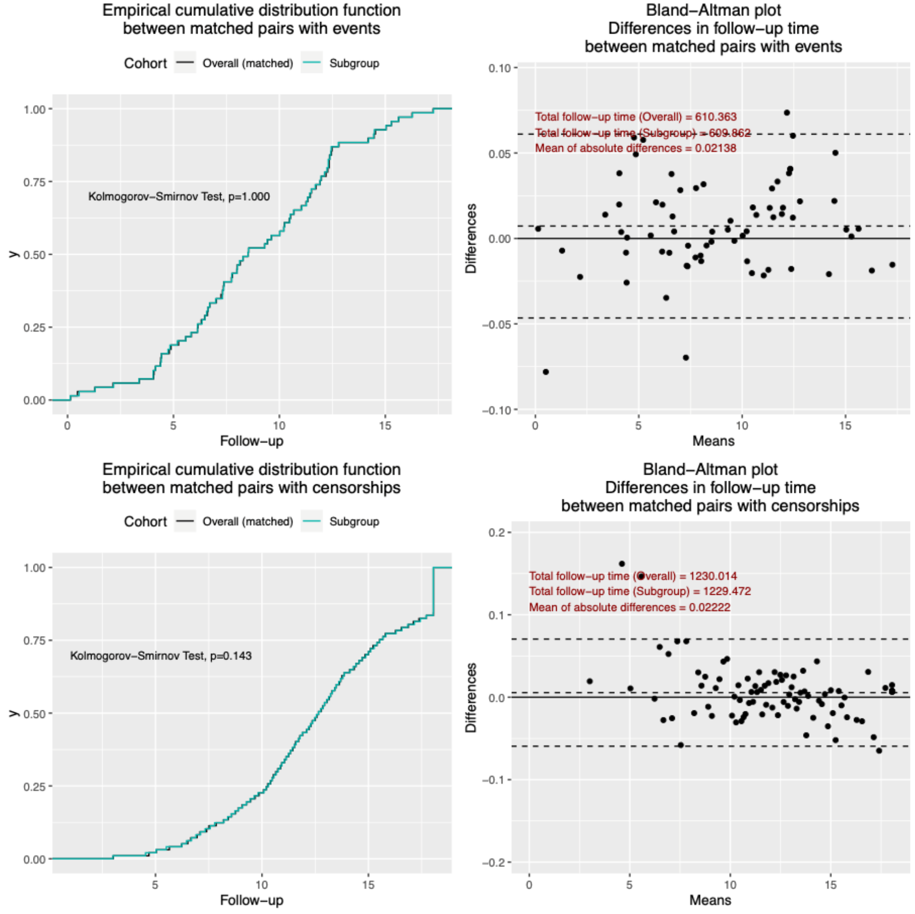

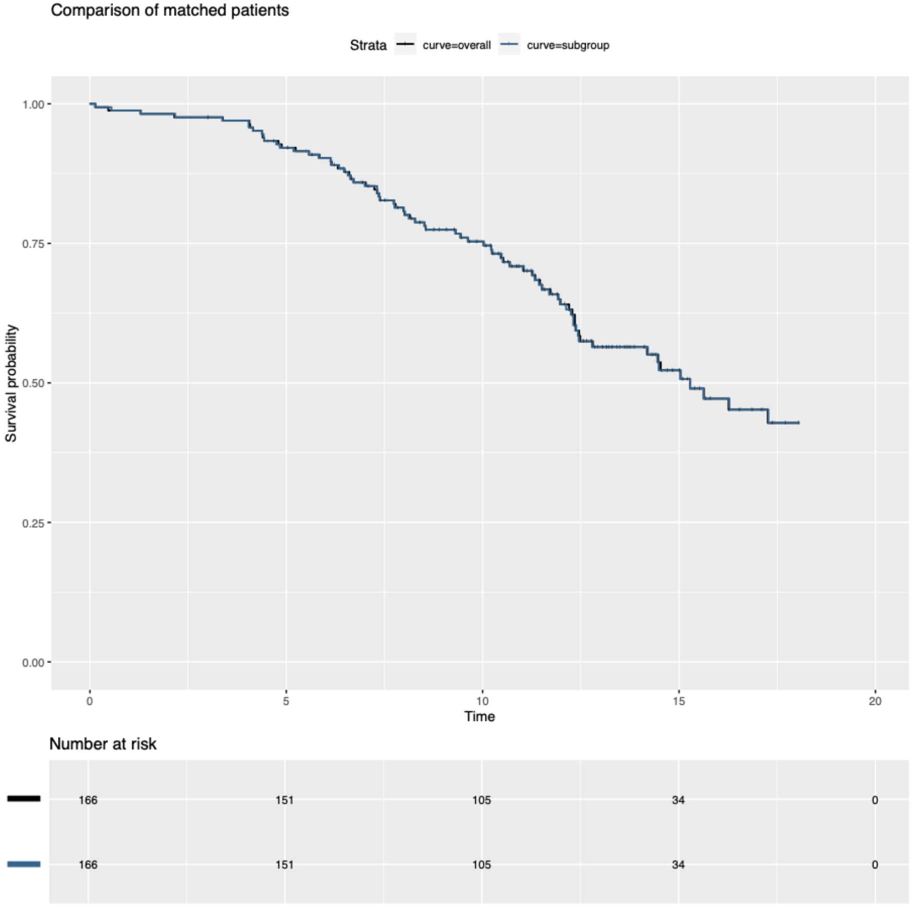

© 2022 Yap DWT et al. *JAMA Oncology*.

KN181 OS CPS ≥10% (Chemo)

Empirical cumulative distribution between follow-up time of matched pairs and Bland-Altman plots to explore discrepancies between matched pairs

Kaplan Meier Curves and Cox-proportional hazard's model comparing matched pairs

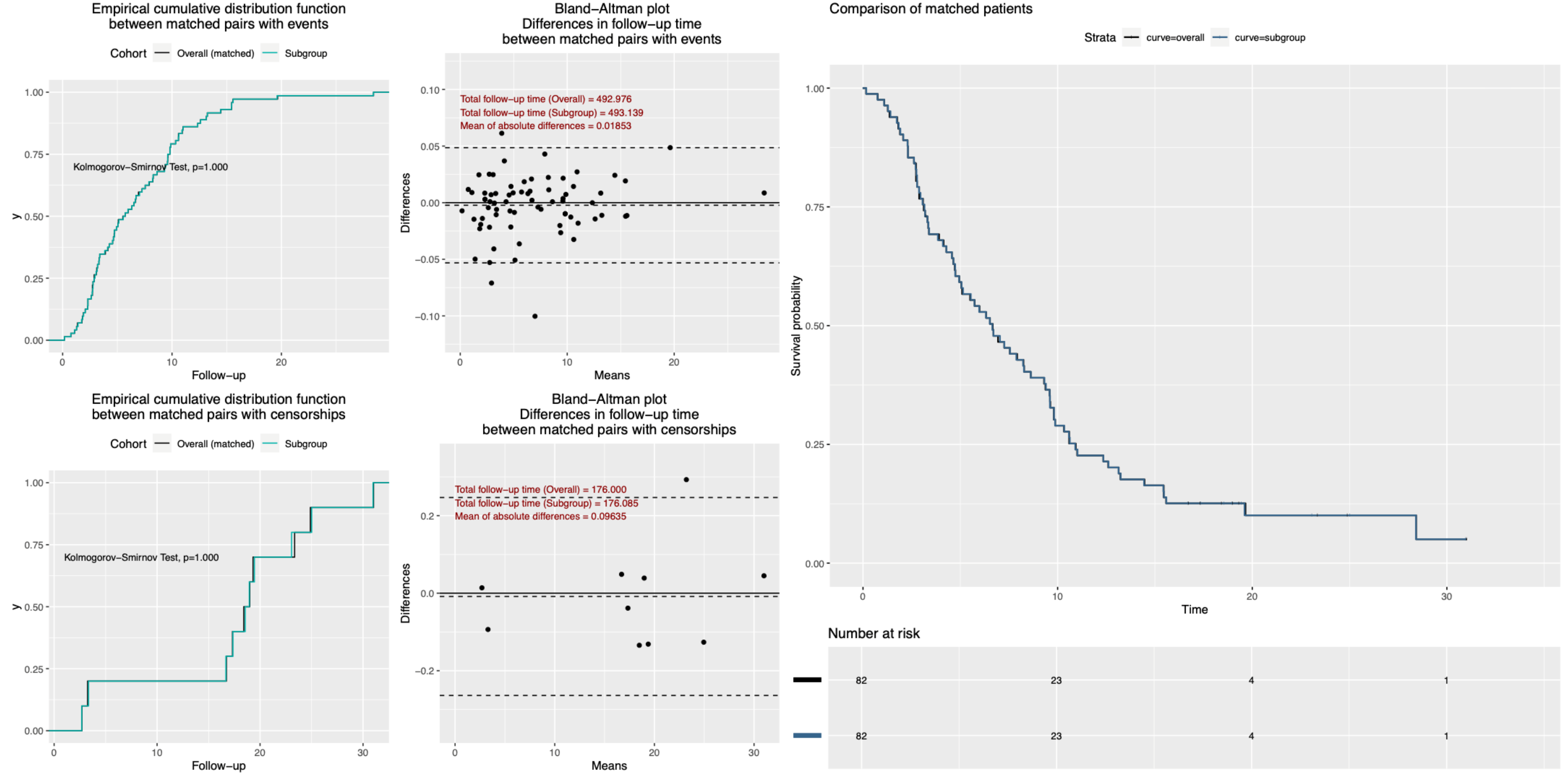

© 2022 Yap DWT et al. *JAMA Oncology*.

KN181 OS CPS ≥10% (Pembro)

Empirical cumulative distribution between follow-up time of matched pairs and Bland-Altman plots to explore discrepancies between matched pairs	Kaplan Meier Curves and Cox-proportional hazard's model comparing matched pairs

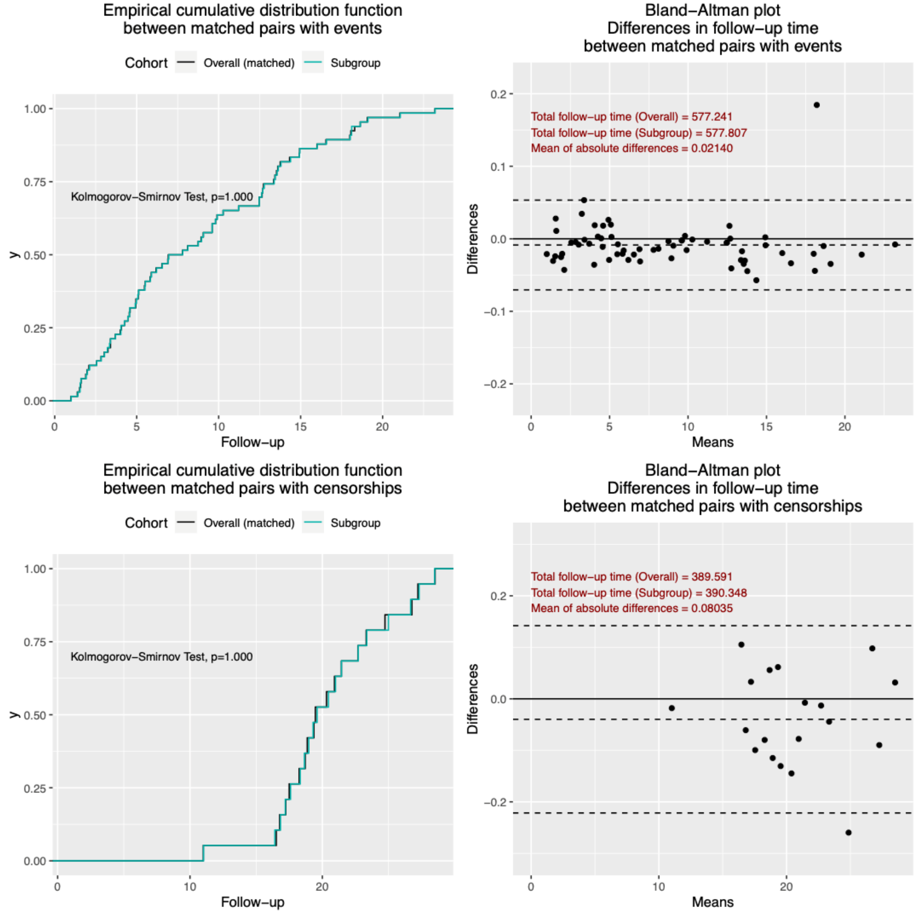

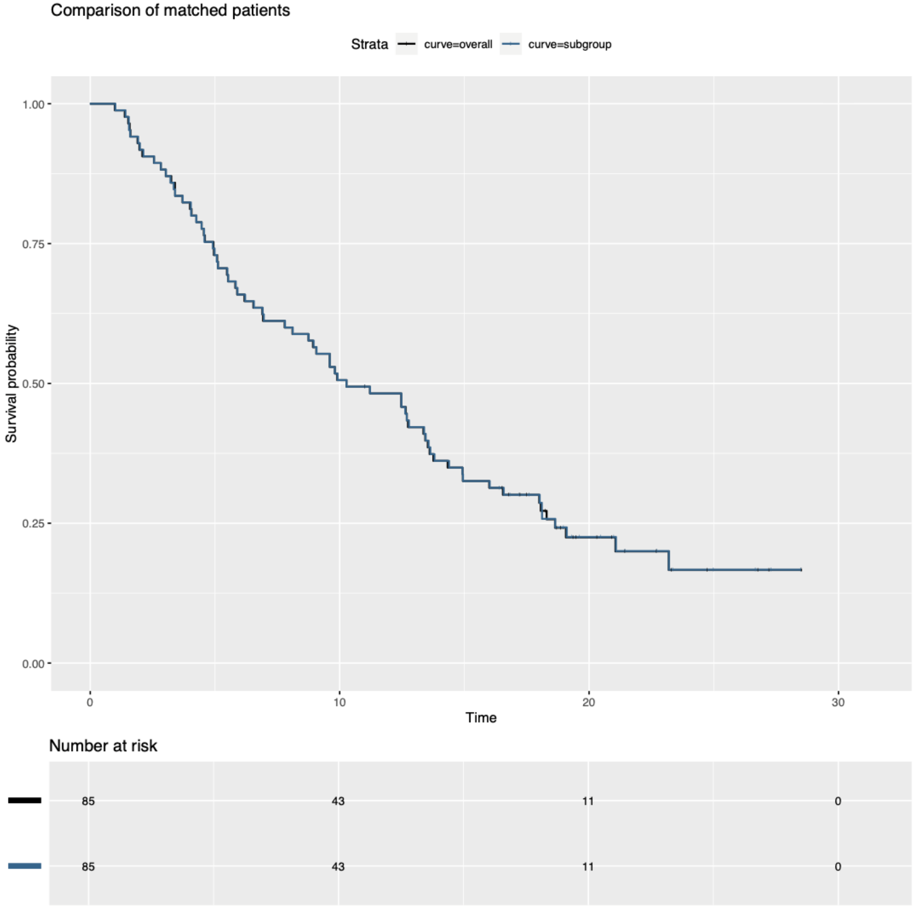

© 2022 Yap DWT et al. *JAMA Oncology*.

RATIONALE302 OS TAP ≥10% (Chemo)	
**Empirical cumulative distribution between follow-up time of matched pairs and Bland-Altman plots to explore discrepancies between matched pairs**	**Kaplan Meier Curves and Cox-proportional hazard's model comparing matched pairs**

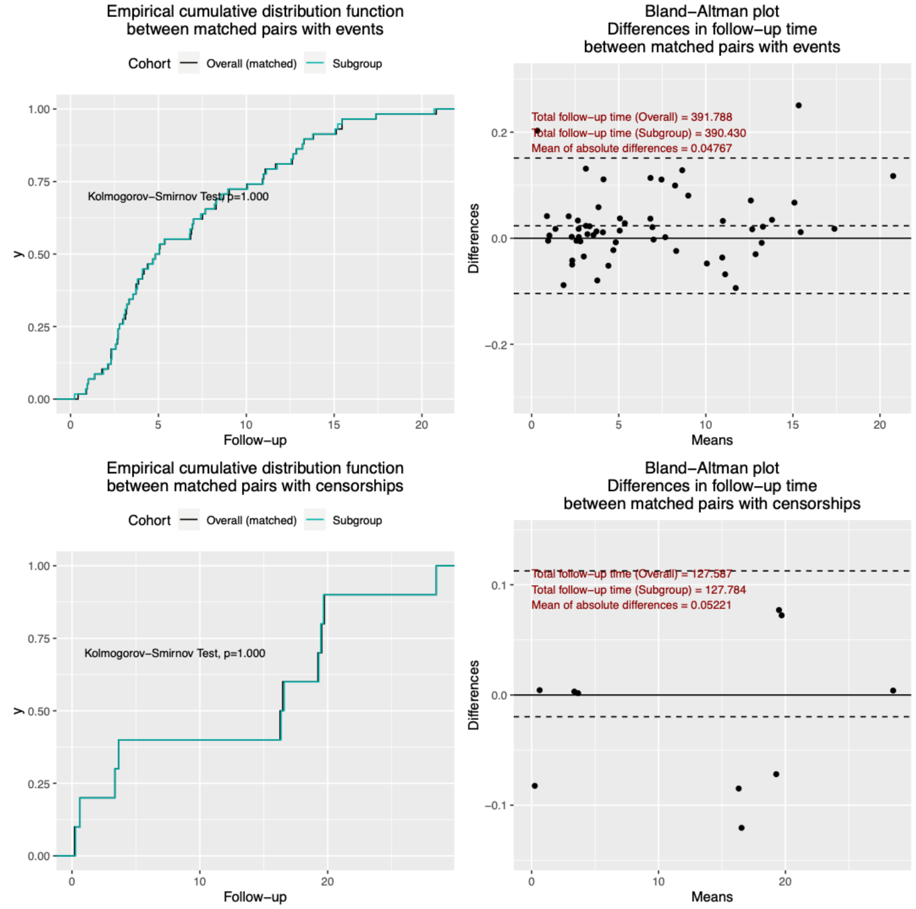

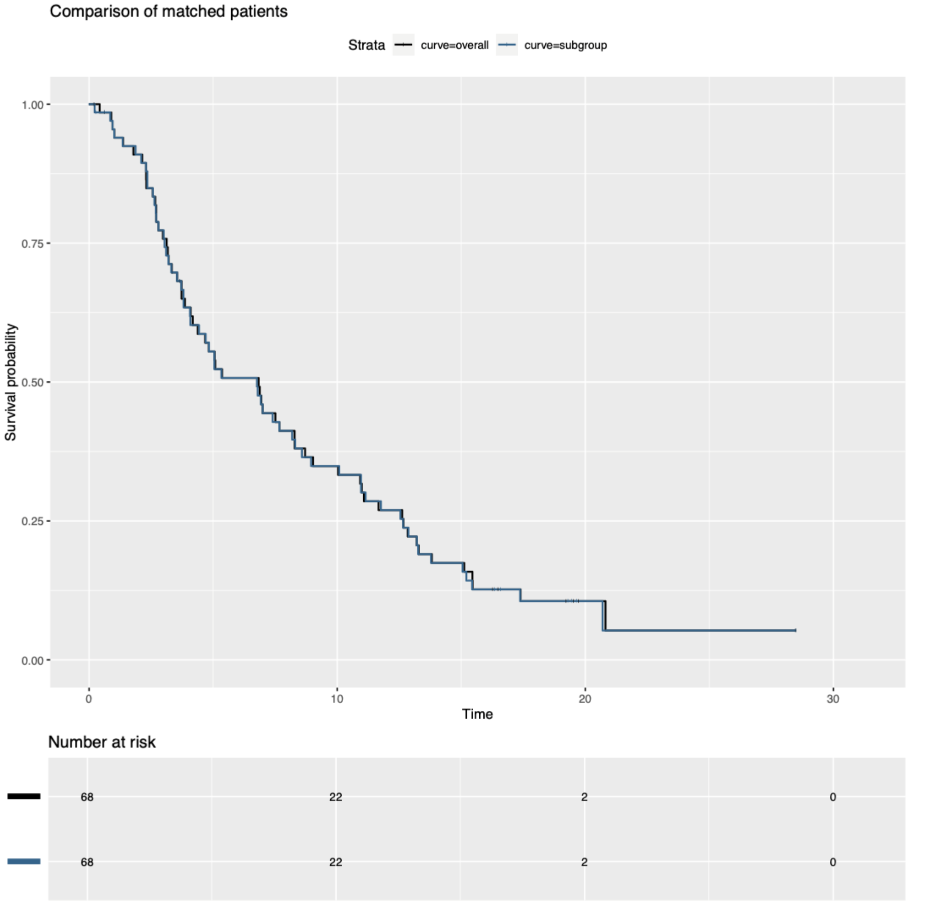

© 2022 Yap DWT et al. *JAMA Oncology*.

RATIONALE302 OS TAP ≥10% (Tisle)

Empirical cumulative distribution between follow-up time of matched pairs and Bland-Altman plots to explore discrepancies between matched pairs	Kaplan Meier Curves and Cox-proportional hazard’s model comparing matched pairs

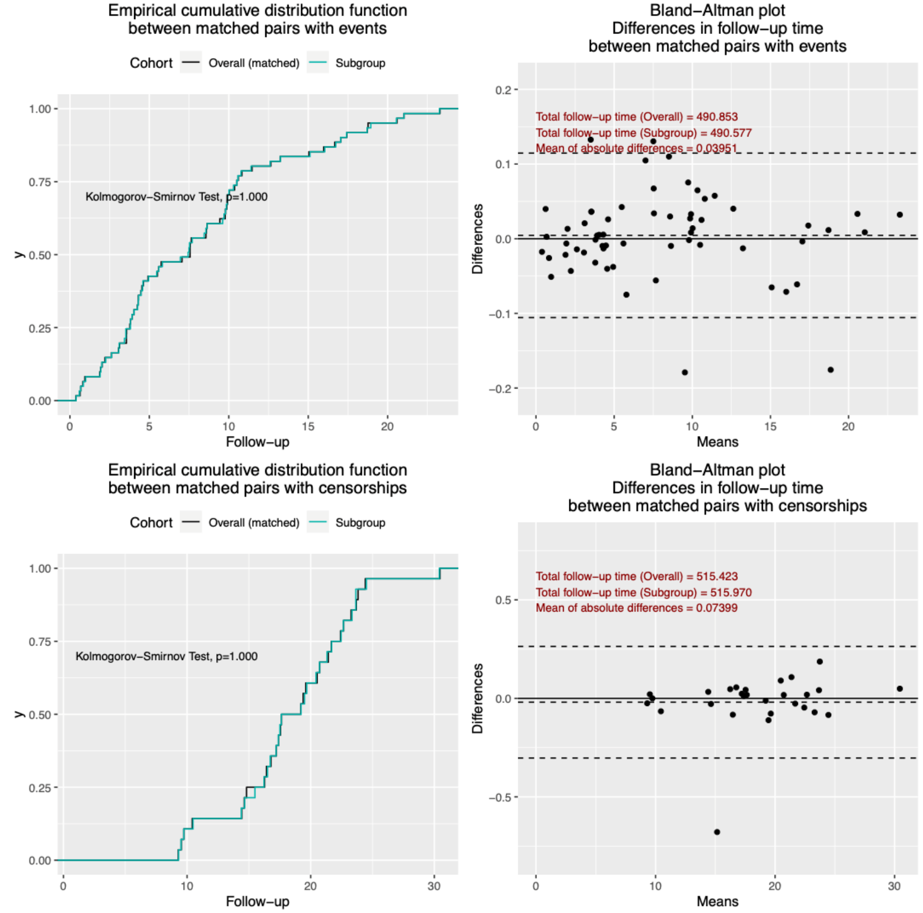

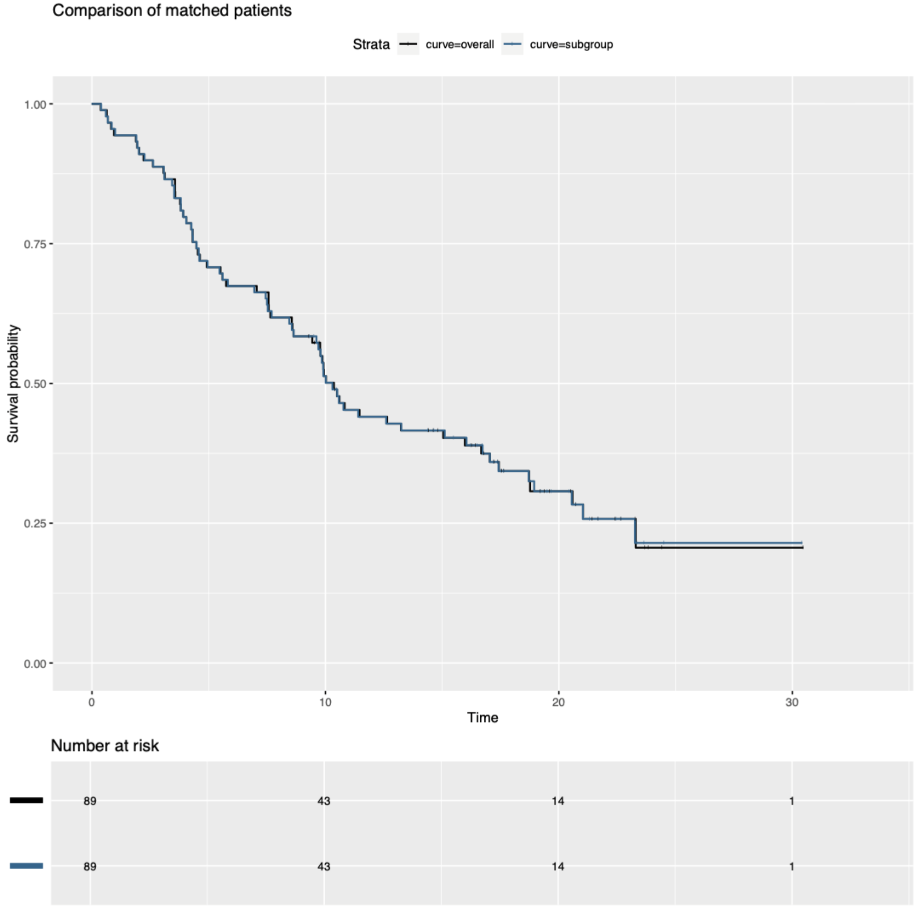

© 2022 Yap DWT et al. *JAMA Oncology*.

ORIENT15 OS CPS ≥10% (Chemo)

Empirical cumulative distribution between follow-up time of matched pairs and Bland-Altman plots to explore discrepancies between matched pairs	Kaplan Meier Curves and Cox-proportional hazard's model comparing matched pairs

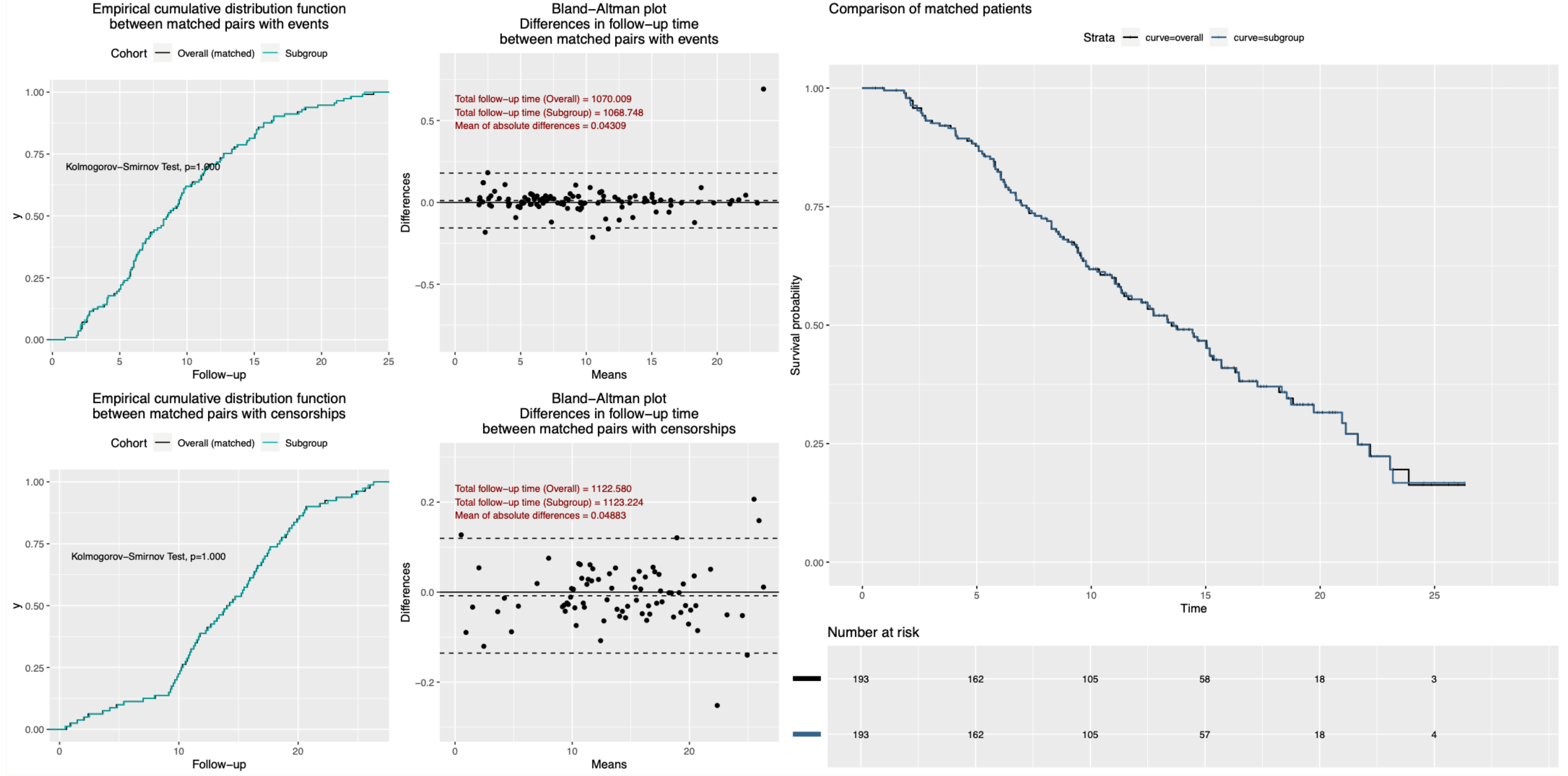

© 2022 Yap DWT et al. *JAMA Oncology*.

ORIENT15 OS CPS ≥10% (Sinti + Chemo)

Empirical cumulative distribution between follow-up time of matched pairs and Bland-Altman plots to explore discrepancies between matched pairs	Kaplan Meier Curves and Cox-proportional hazard’s model comparing matched pairs

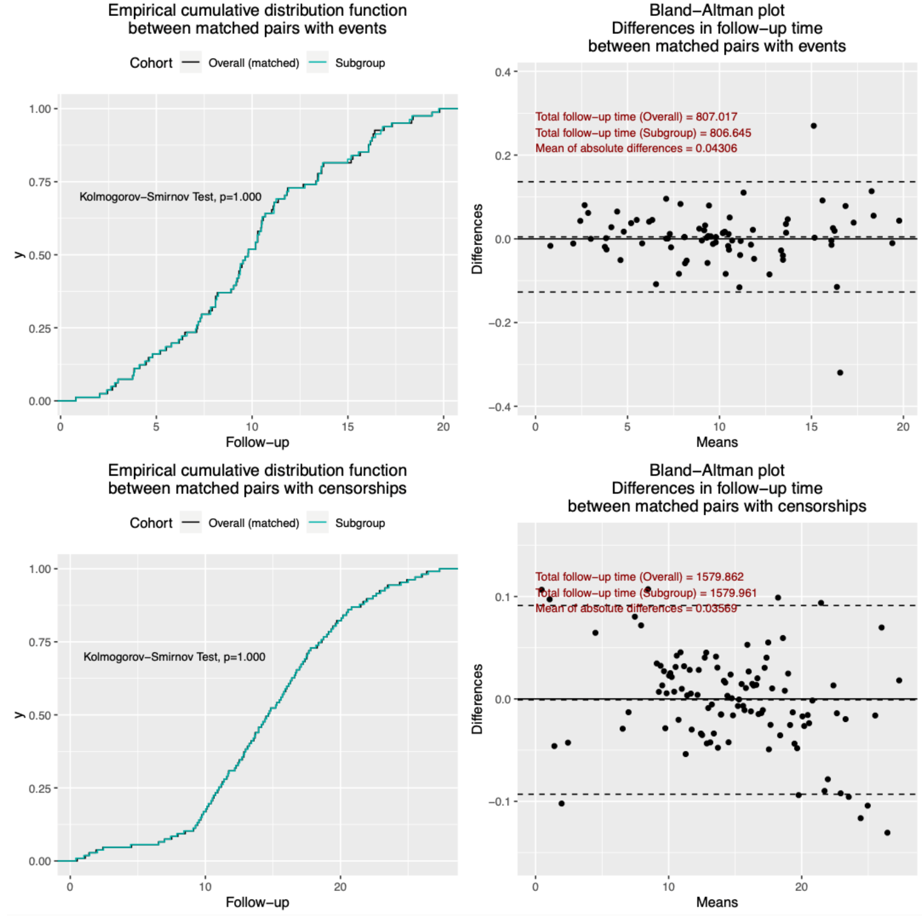

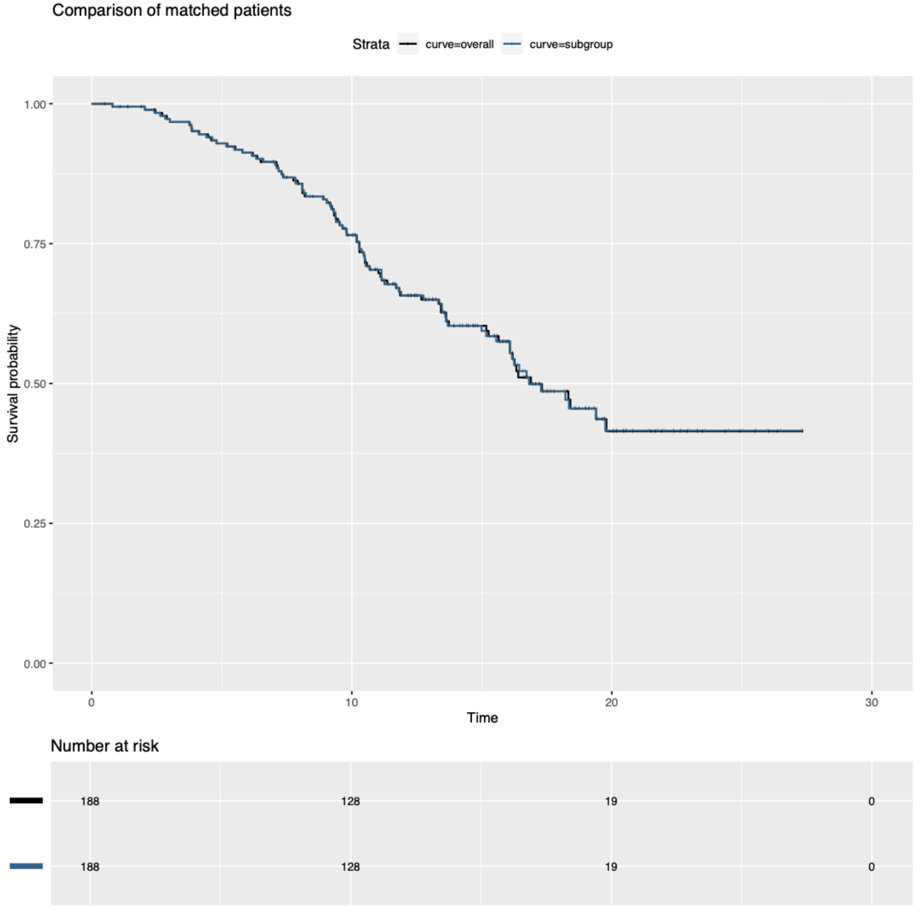

© 2022 Yap DWT et al. *JAMA Oncology*.

ORIENT15 PFS CPS ≥10% (Chemo)

Empirical cumulative distribution between follow-up time of matched pairs and Bland-Altman plots to explore discrepancies between matched pairs	Kaplan Meier Curves and Cox-proportional hazard's model comparing matched pairs

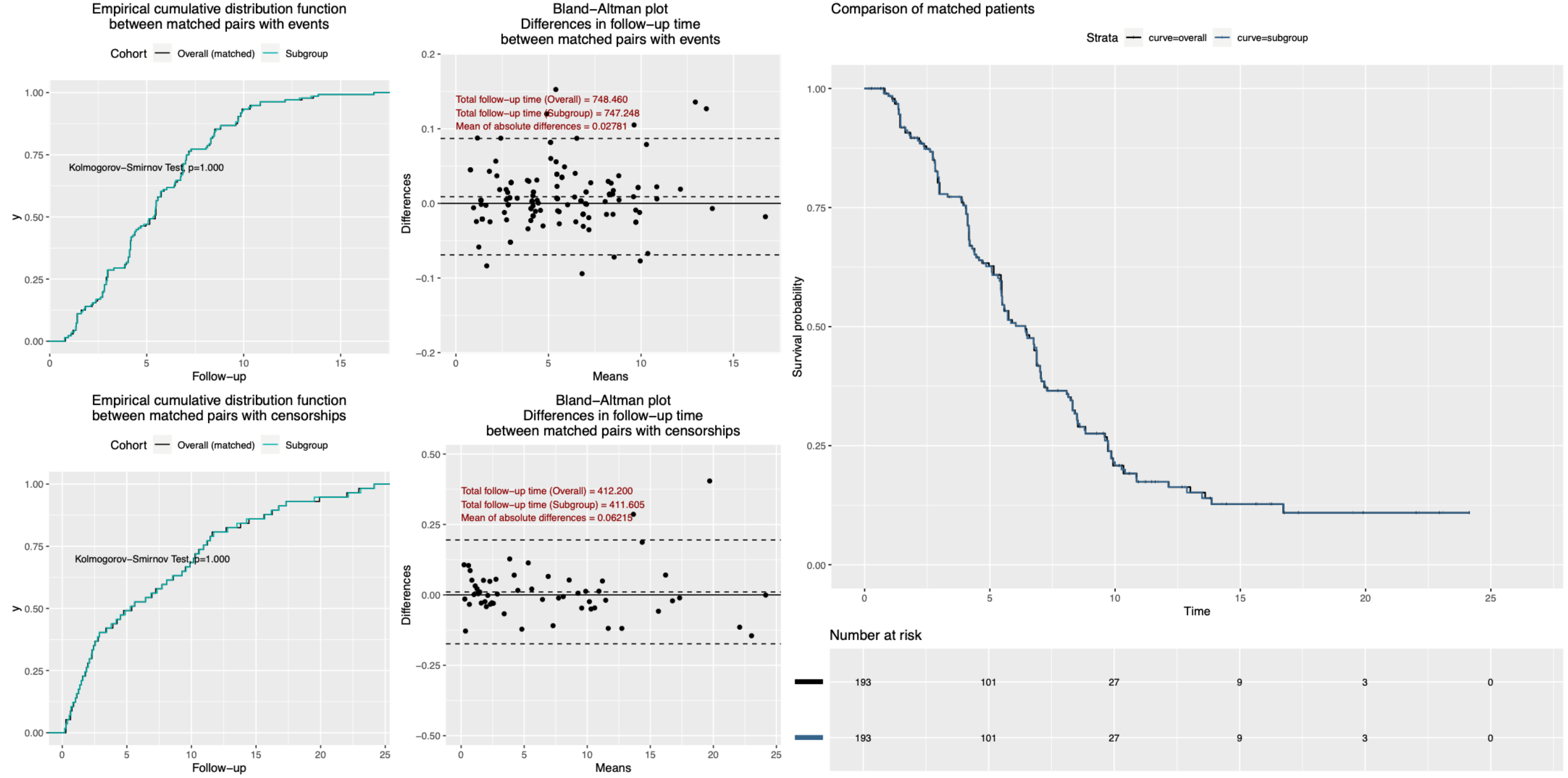

© 2022 Yap DWT et al. *JAMA Oncology*.

ORIENT15 PFS CPS ≥10% (Sinti + Chemo)

Empirical cumulative distribution between follow-up time of matched pairs and Bland-Altman plots to explore discrepancies between matched pairs	Kaplan Meier Curves and Cox-proportional hazard's model comparing matched pairs
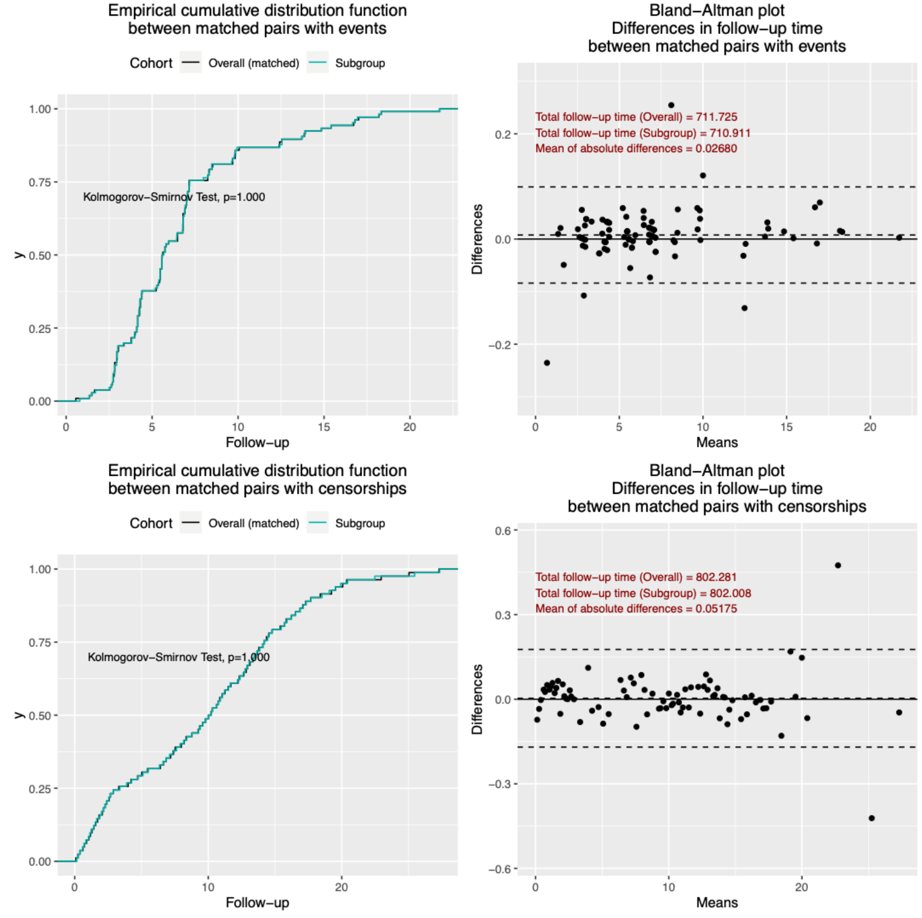	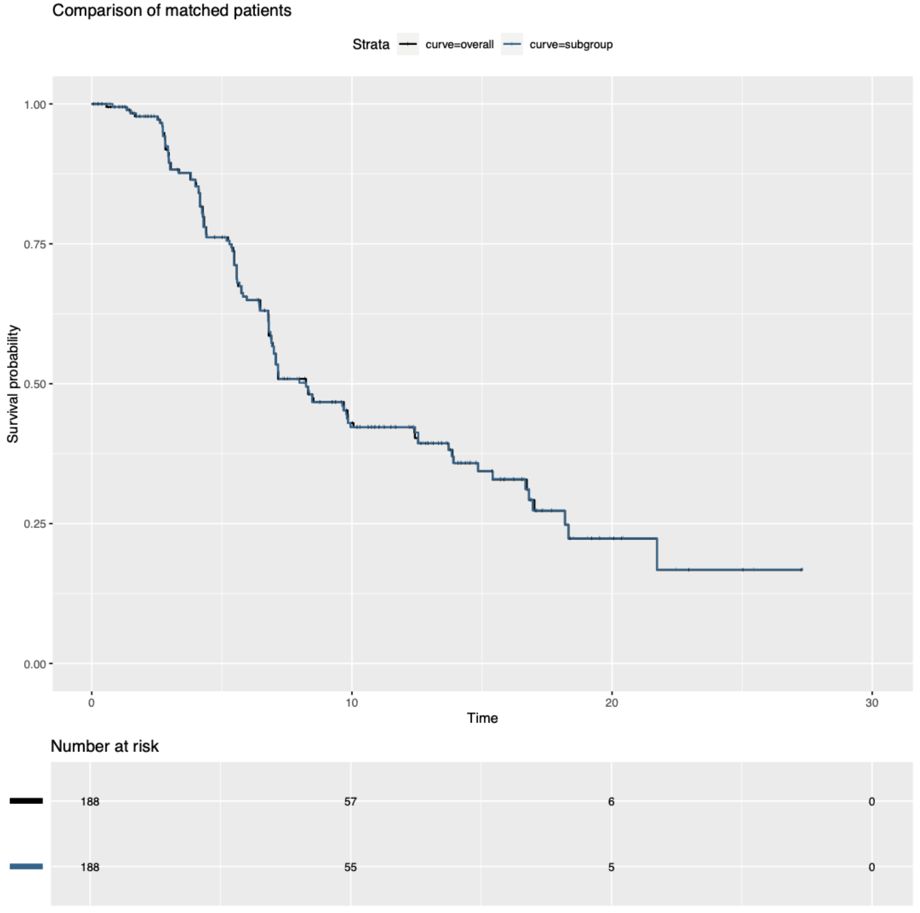

© 2022 Yap DWT et al. *JAMA Oncology*.

KN590 OS CPS ≥10% (Chemo)

Empirical cumulative distribution between follow-up time of matched pairs and Bland-Altman plots to explore discrepancies between matched pairs	Kaplan Meier Curves and Cox-proportional hazard's model comparing matched pairs
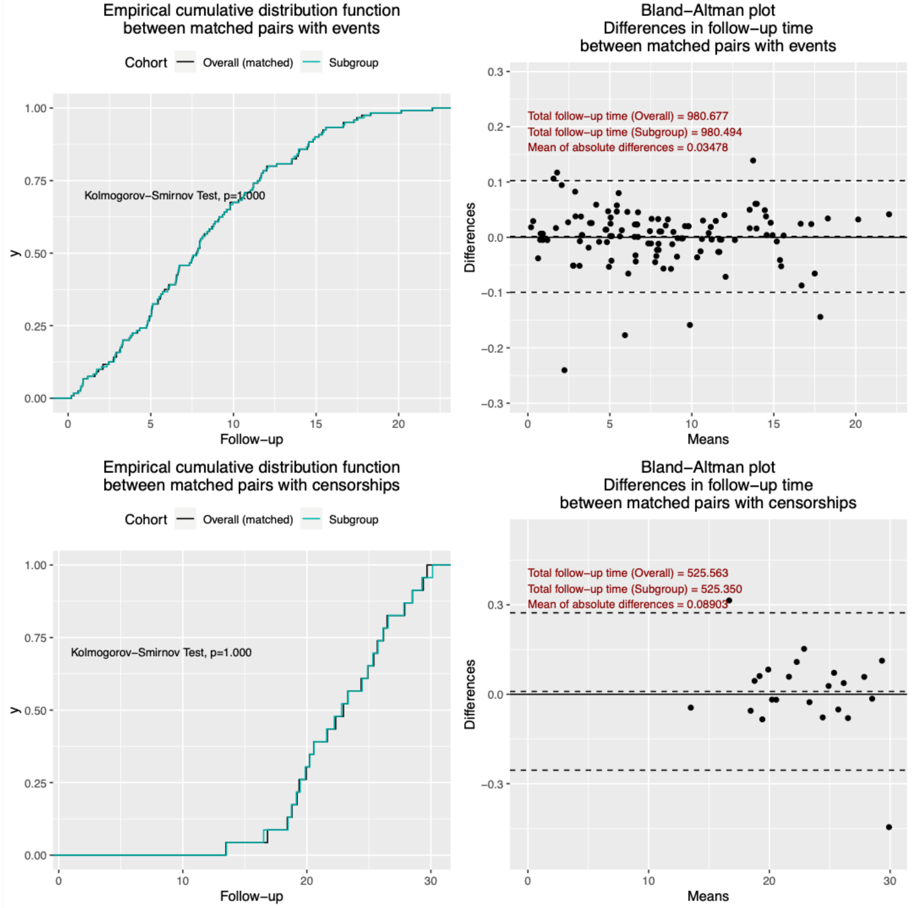	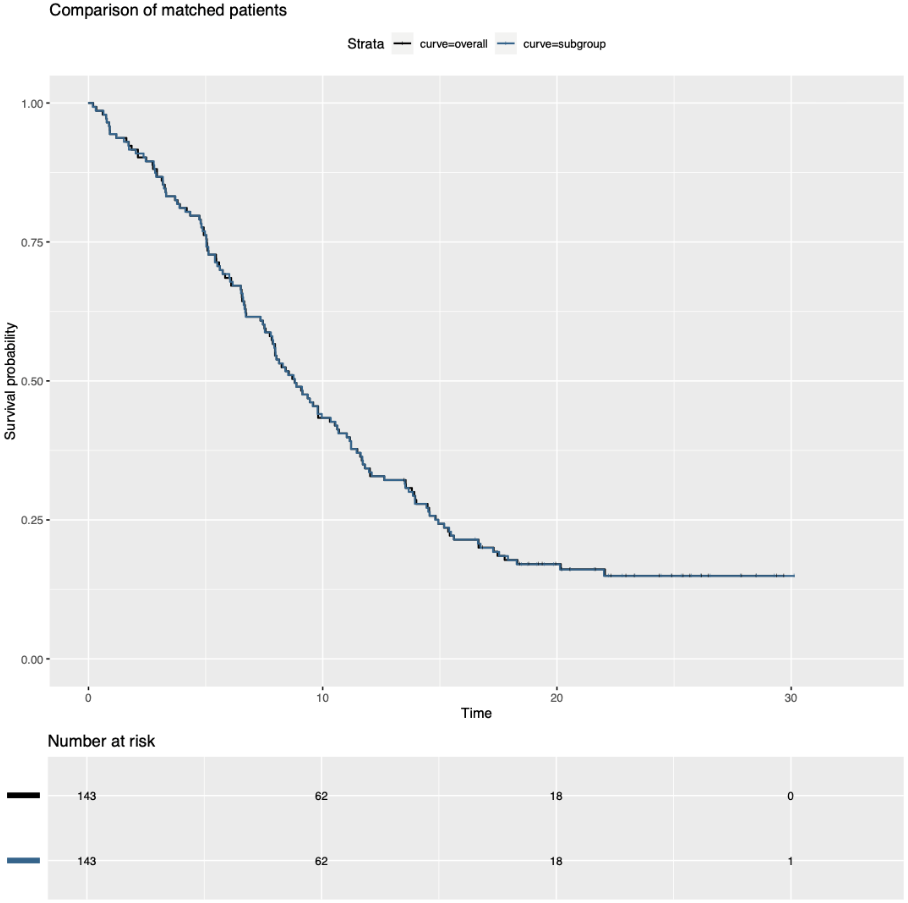

© 2022 Yap DWT et al. *JAMA Oncology*.

KN590 OS CPS ≥10% (Pembro + Chemo)

Empirical cumulative distribution between follow-up time of matched pairs and Bland-Altman plots to explore discrepancies between matched pairs	Kaplan Meier Curves and Cox-proportional hazard's model comparing matched pairs
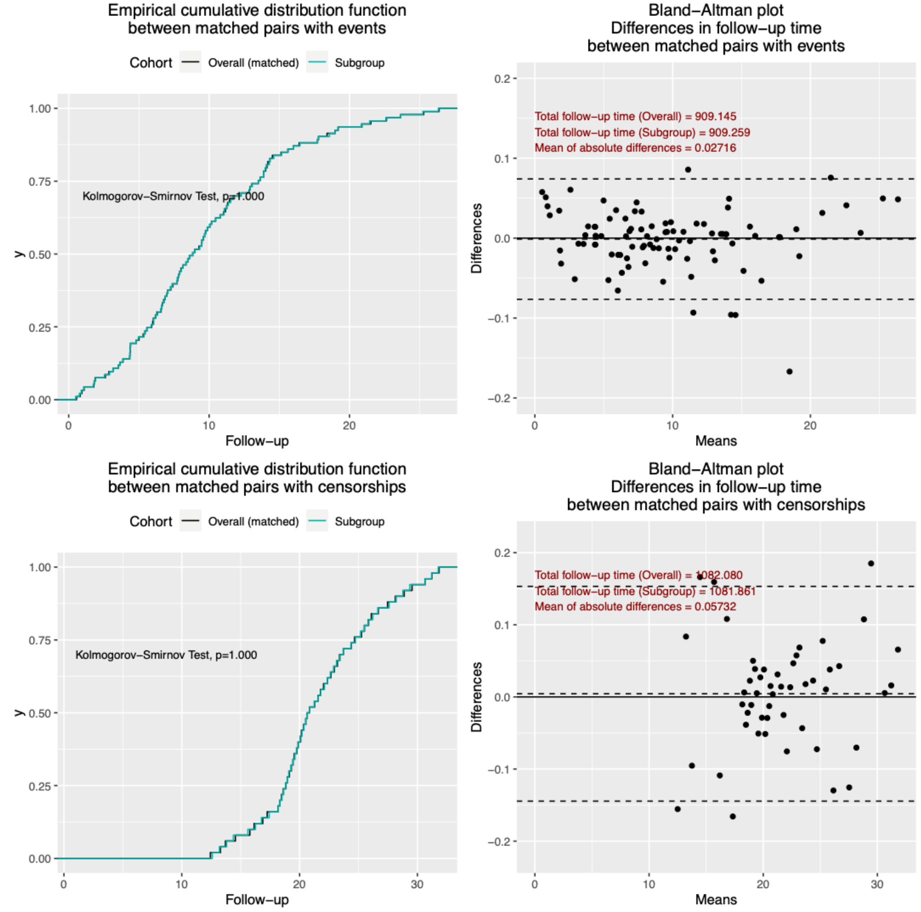	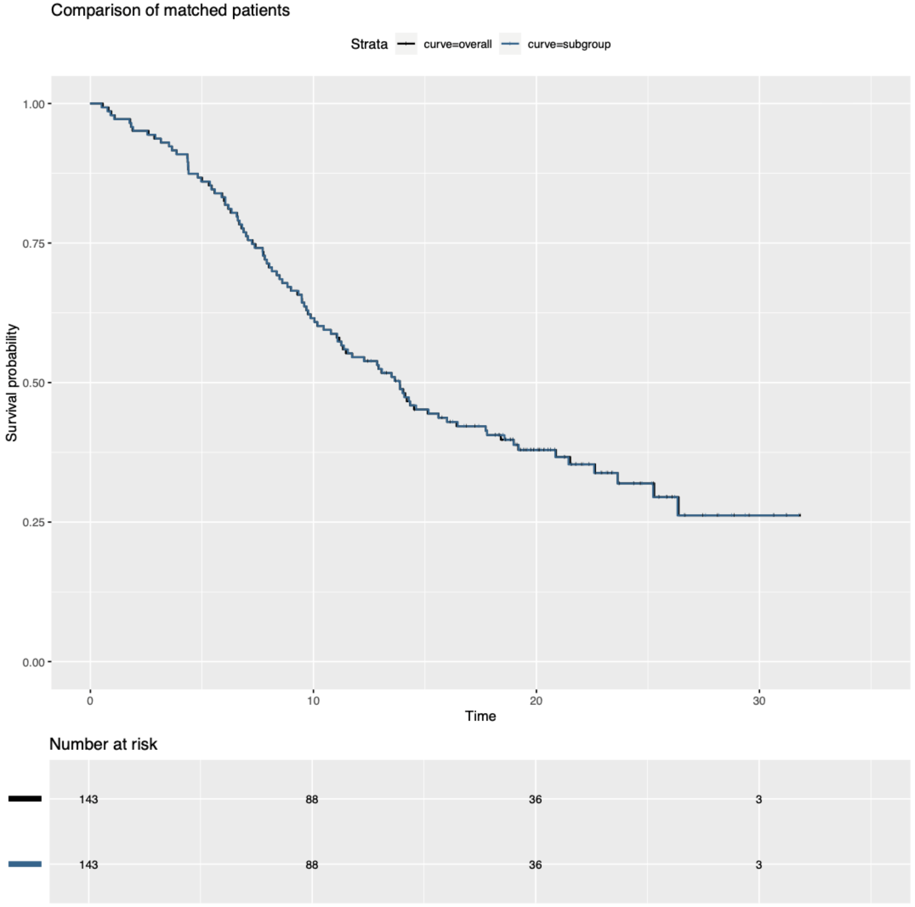

© 2022 Yap DWT et al. *JAMA Oncology*.

ATTRACTION3 OS TPS ≥1% (Chemo)

Empirical cumulative distribution between follow-up time of matched pairs and Bland-Altman plots to explore discrepancies between matched pairs	Kaplan Meier Curves and Cox-proportional hazard's model comparing matched pairs
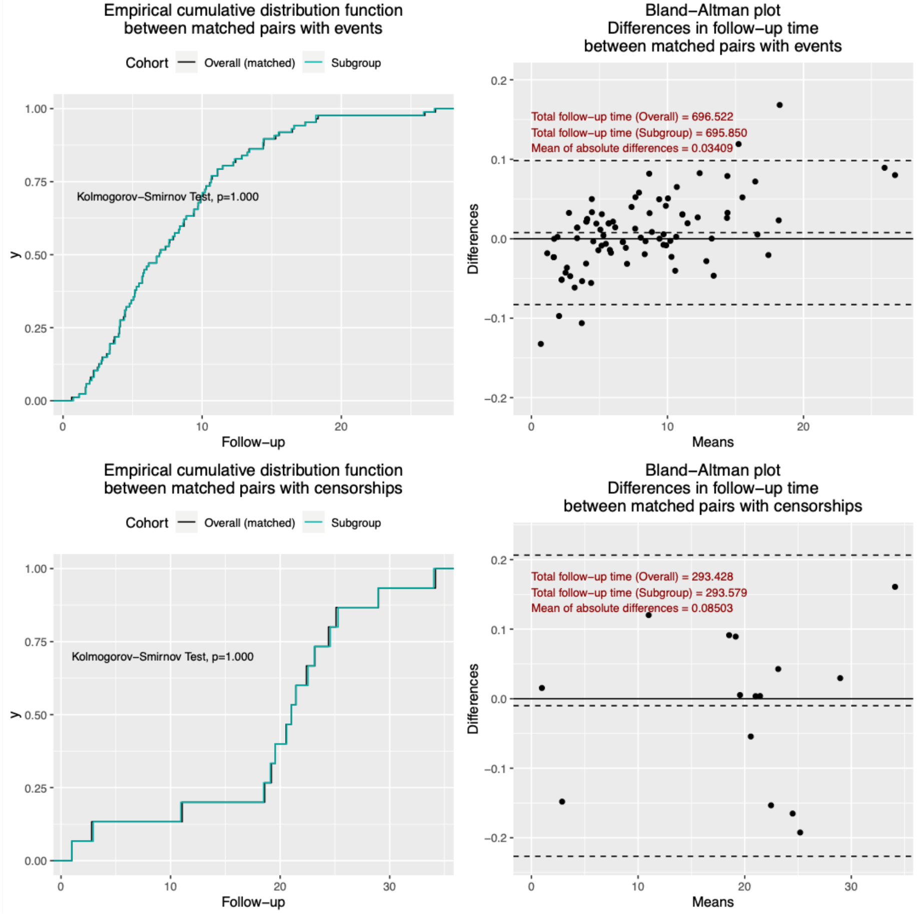	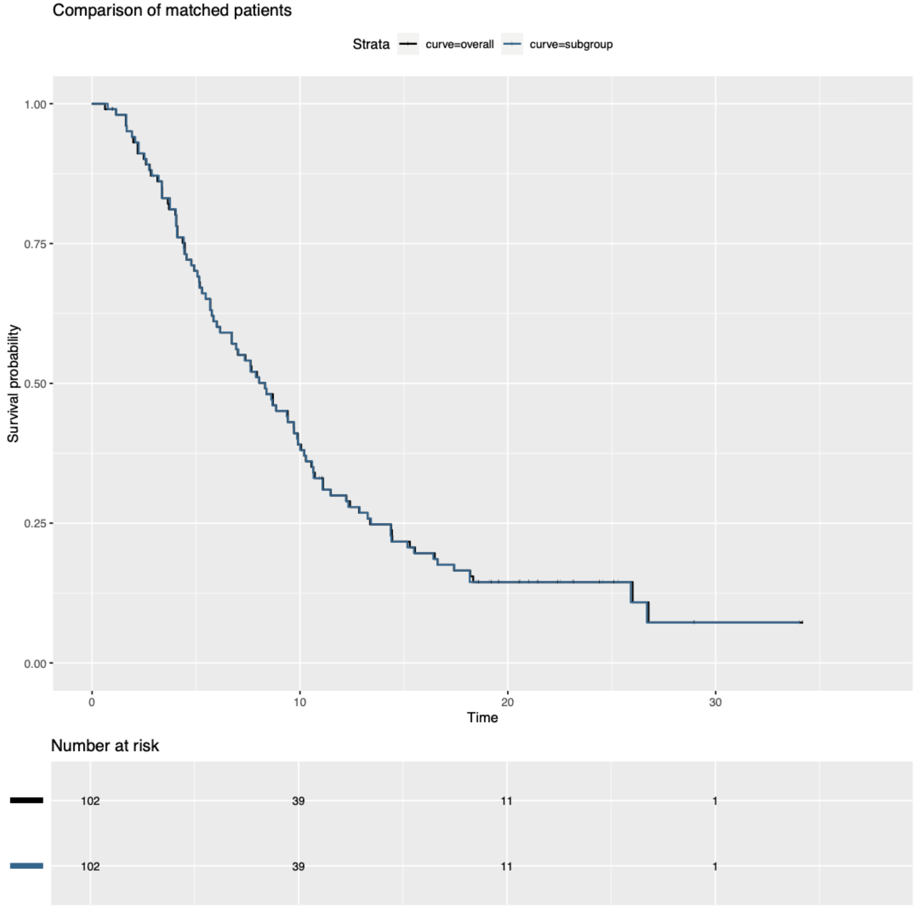

© 2022 Yap DWT et al. *JAMA Oncology*.

ATTRACTION3 OS TPS ≥1% (Nivo)

Empirical cumulative distribution between follow-up time of matched pairs and Bland-Altman plots to explore discrepancies between matched pairs	Kaplan Meier Curves and Cox-proportional hazard's model comparing matched pairs
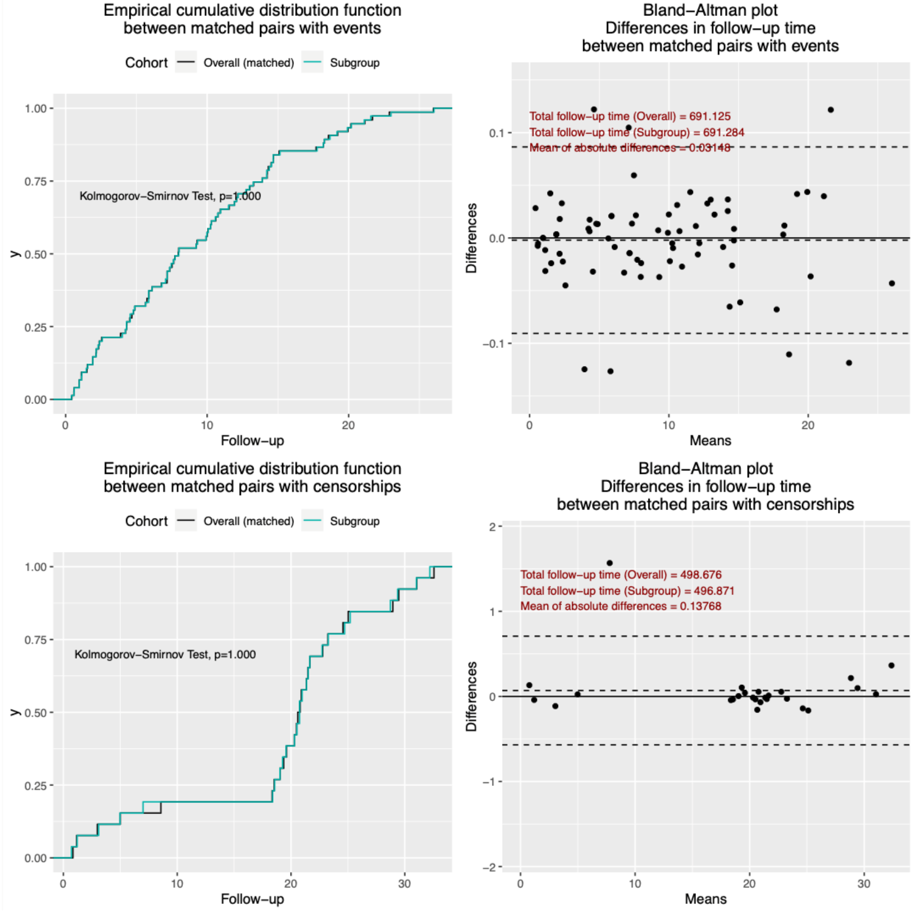	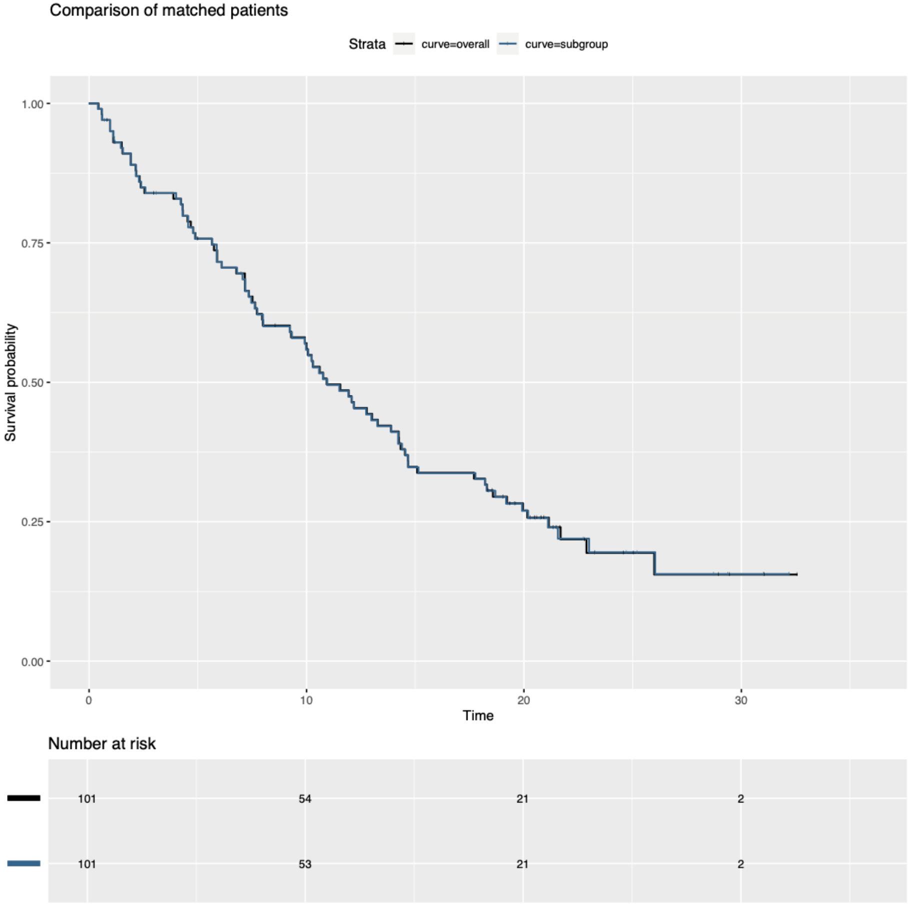

© 2022 Yap DWT et al. *JAMA Oncology*.

ESCORT OS TPS ≥1% (Chemo)

Empirical cumulative distribution between follow-up time of matched pairs and Bland-Altman plots to explore discrepancies between matched pairs	Kaplan Meier Curves and Cox-proportional hazard's model comparing matched pairs

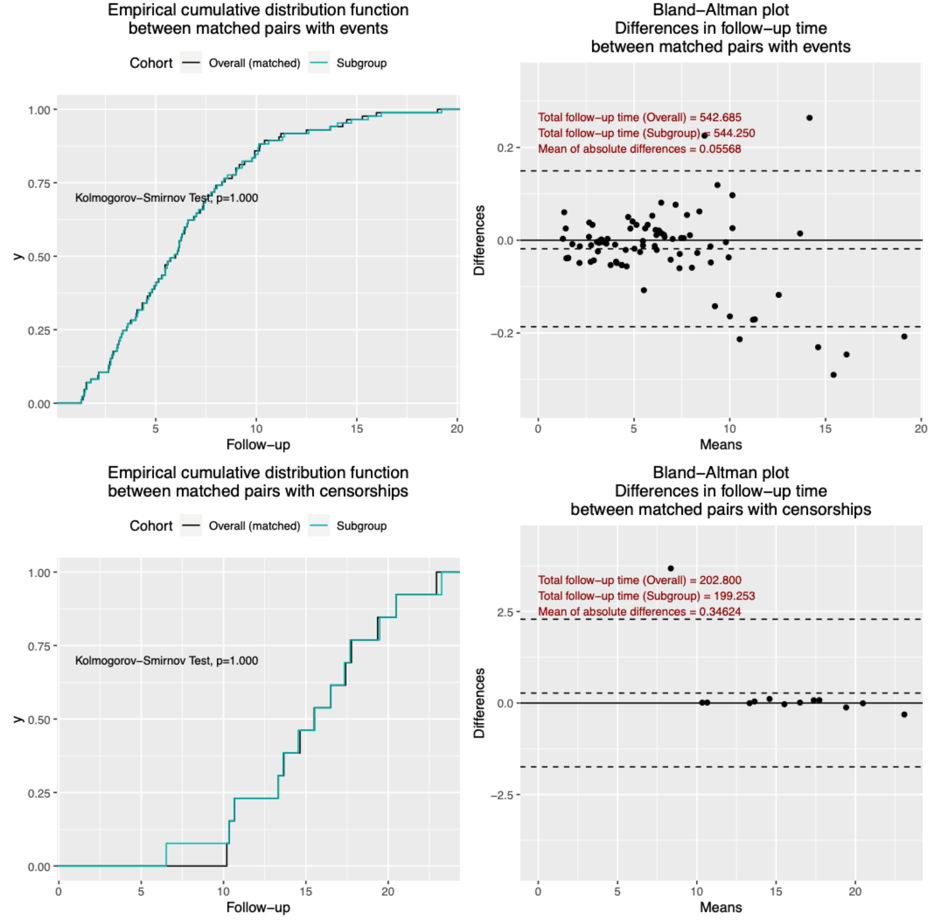

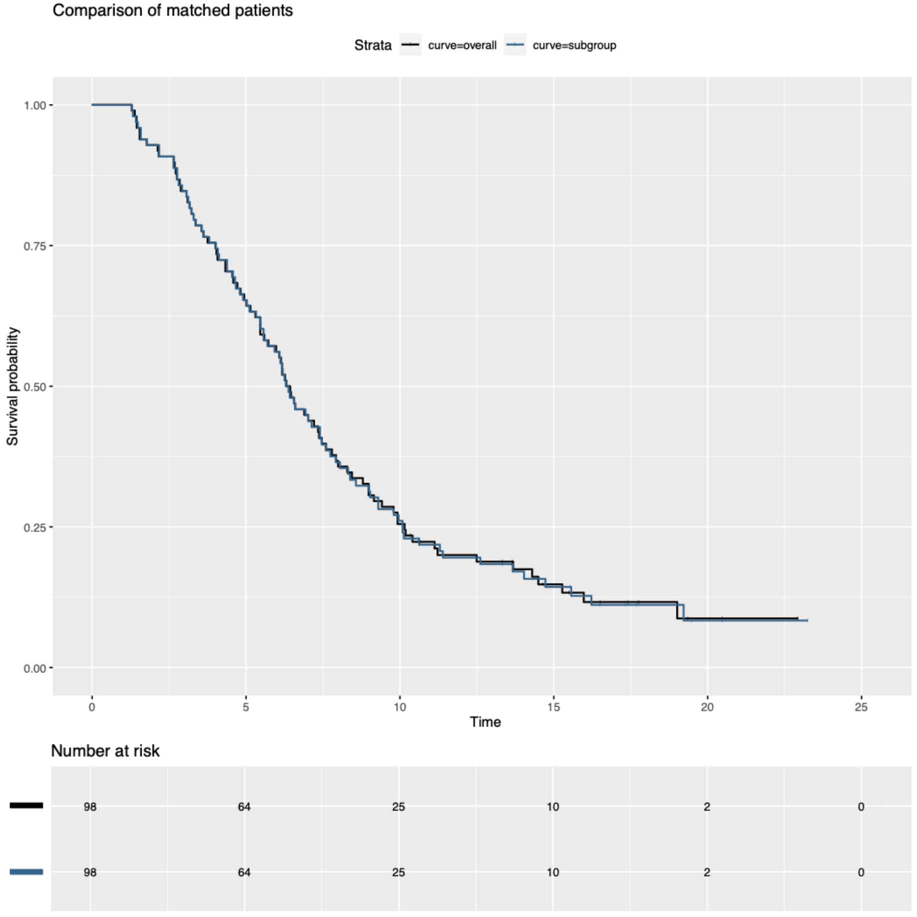

© 2022 Yap DWT et al. *JAMA Oncology*.

ESCORT OS TPS ≥1% (Camre)

Empirical cumulative distribution between follow-up time of matched pairs and Bland-Altman plots to explore discrepancies between matched pairs	Kaplan Meier Curves and Cox-proportional hazard’s model comparing matched pairs

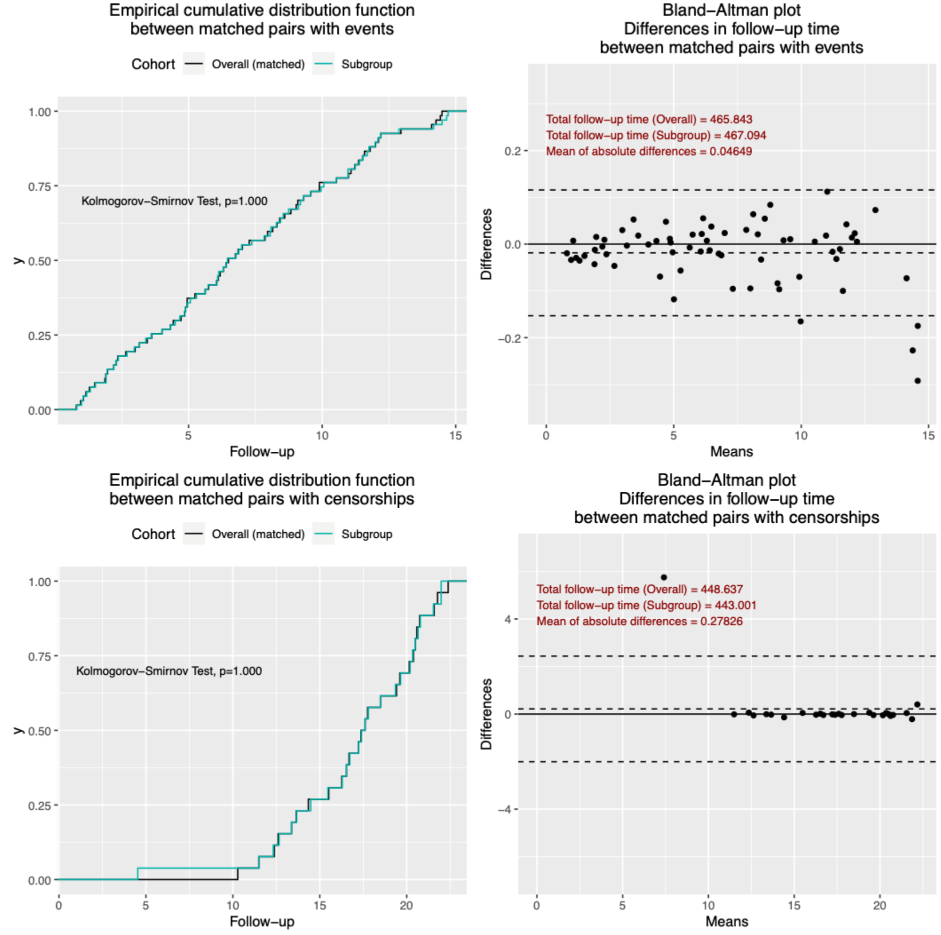

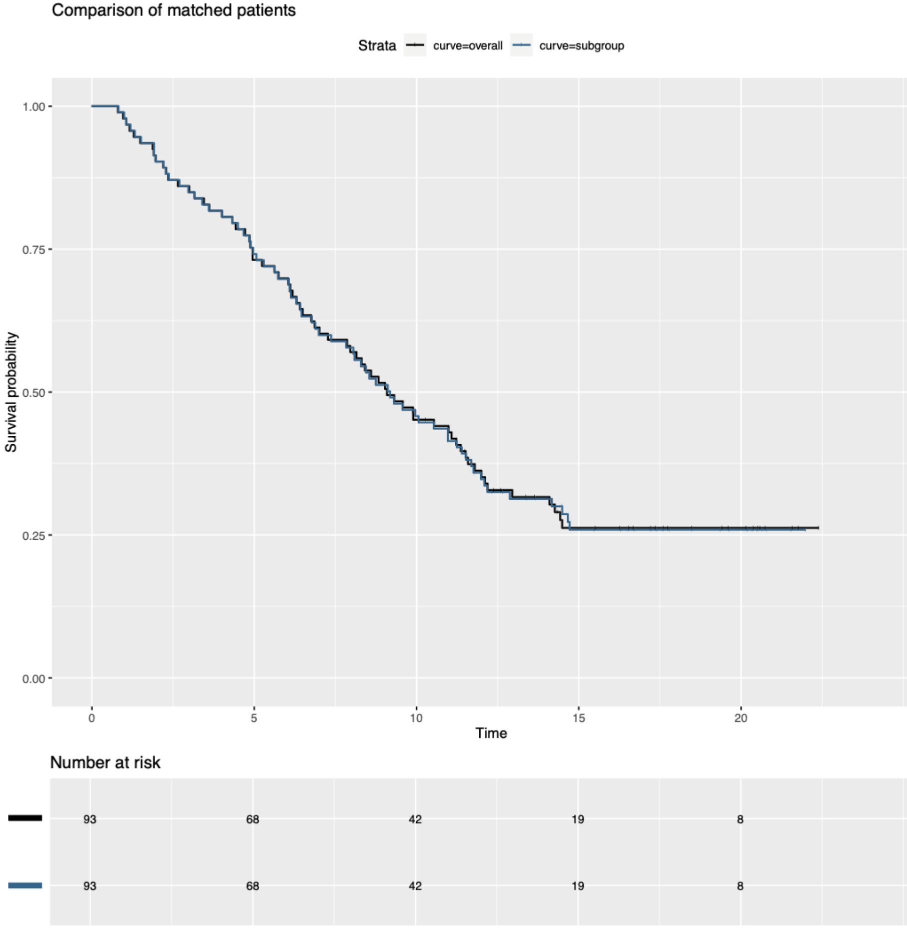

Abbreviations: Nivo, nivolumab; Ipi, ipilimumab; Camre, camrelizumab; Pembro, pembrolizumab; Sinti, sintilimab; Tisle, Tislelizumab; Chemo, chemotherapy; CPS, combined positive score; TPS, tumor proportion score; TAP, tumor area positivity; CM648, CheckMate 648; KN590, KEYNOTE-590; KN181, KEYNOTE-181.

© 2022 Yap DWT et al. *JAMA Oncology*.

eFigure 6. Convergence Plots and Histograms of Simulations

Histogram of ln(HR)<br>CM648, DOR, Chemo, TPS≥1%<br>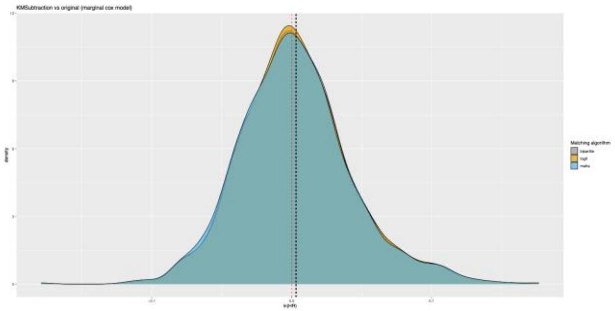	Histogram of |ln(HR)| CM648, DOR, Chemo, TPS≥1%<br>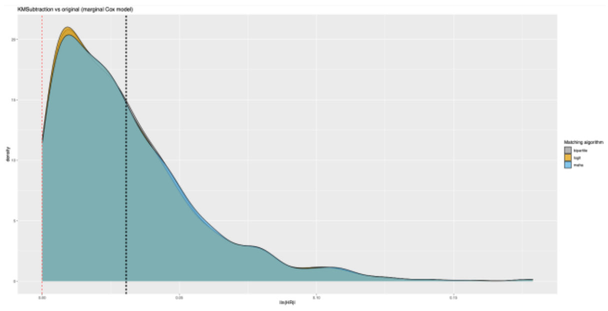	Convergence plot<br>CM648, DOR, Chemo, TPS≥1%<br>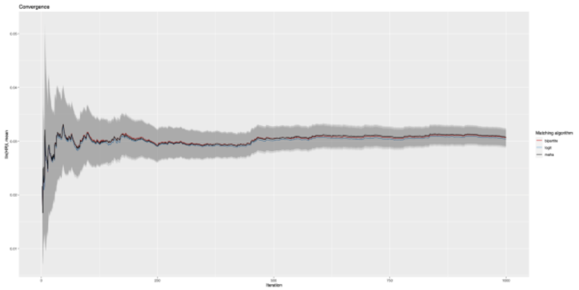
Histogram of ln(HR)<br>CM648, DOR, Nivo+Ipi, TPS≥1%<br>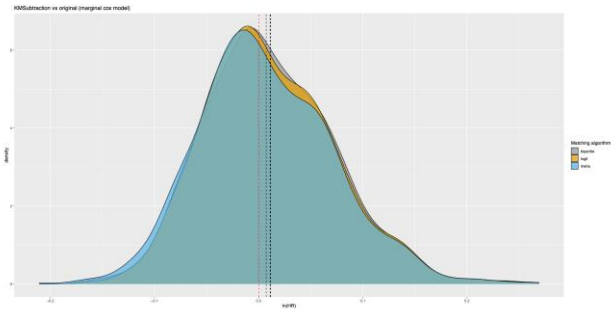	Histogram of |ln(HR)| CM648, DOR, Nivo+Ipi, TPS≥1%<br>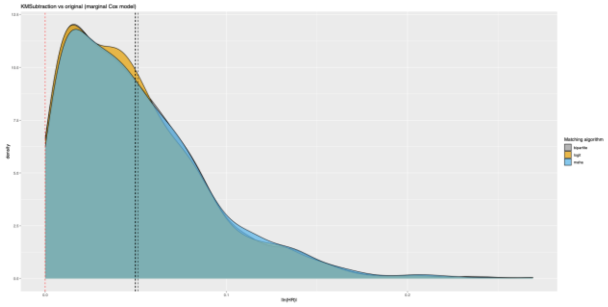	Convergence plot<br>CM648, DOR, Nivo+Ipi, TPS≥1%<br>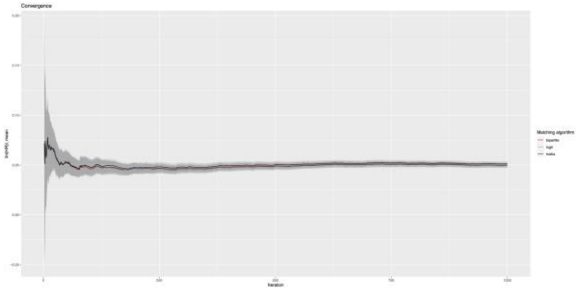

© 2022 Yap DWT et al. *JAMA Oncology*.

Histogram of ln(HR)<br>CM648, DOR, Nivo+Chemo, TPS≥1%<br>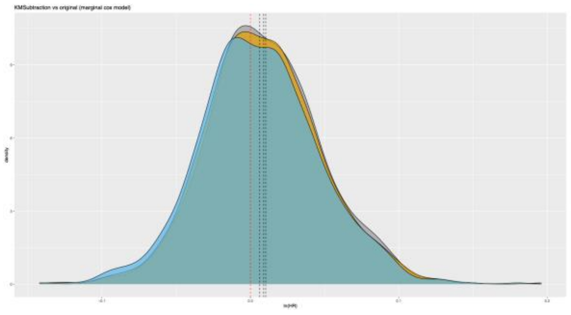	Histogram of |ln(HR)| CM648, DOR, Nivo+Chemo, TPS≥1%<br>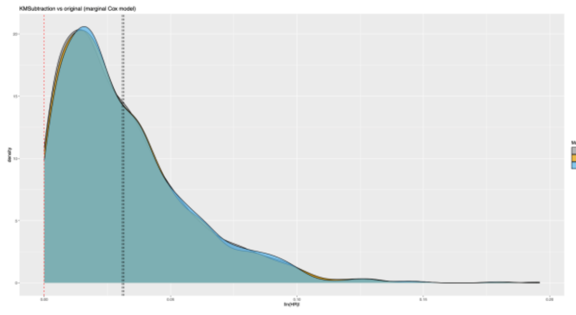	Convergence plot<br>CM648, DOR, Nivo+Chemo, TPS≥1%<br>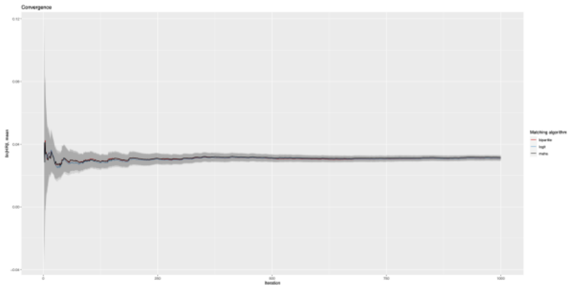
Histogram of ln(HR)<br>CM648, OS, Chemo, TPS≥1%<br>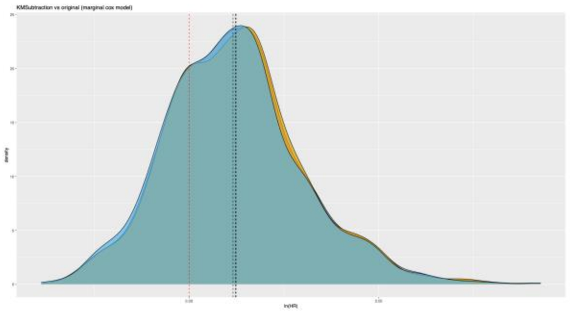	Histogram of |ln(HR)| CM648, OS, Chemo, TPS≥1%<br>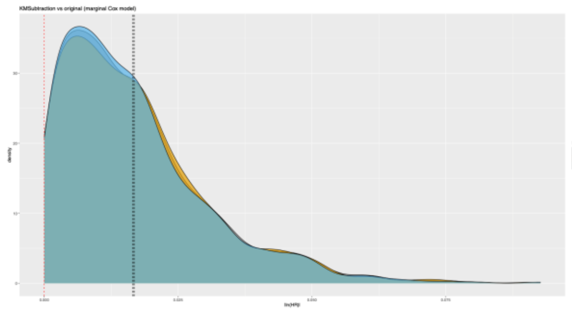	Convergence plot<br>CM648, OS, Chemo, TPS≥1%<br>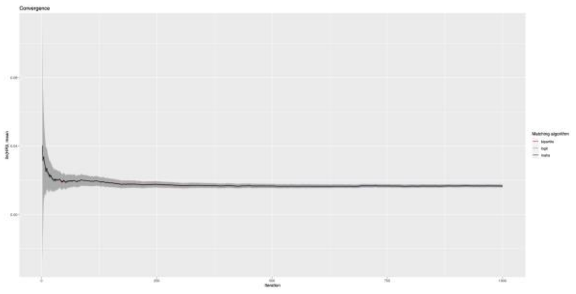
Histogram of ln(HR)<br>CM648, OS, Nivo+Ipi, TPS≥1%<br>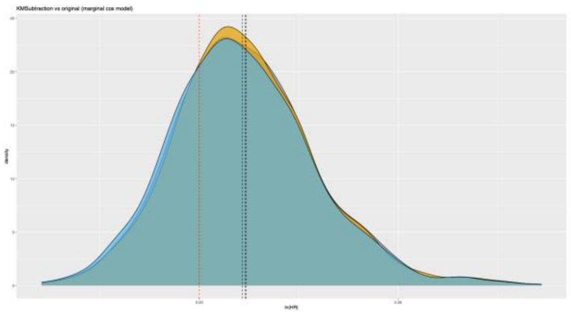	Histogram of |ln(HR)| CM648, OS, Nivo+Ipi, TPS≥1%<br>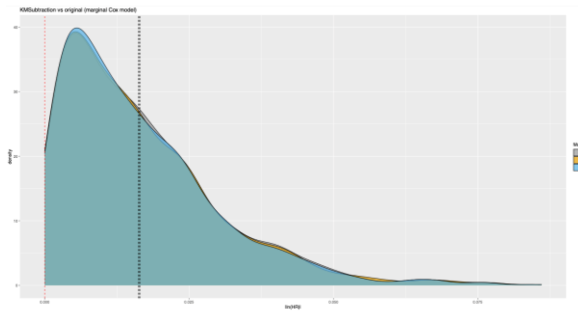	Convergence plot<br>CM648, OS, Nivo+Ipi, TPS≥1%<br>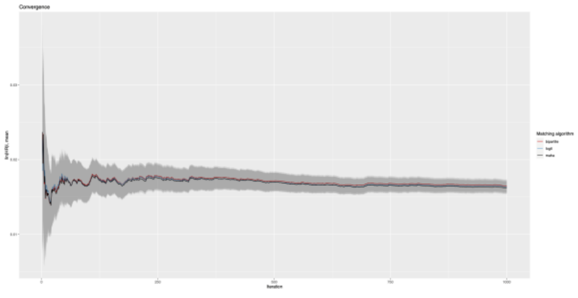

© 2022 Yap DWT et al. *JAMA Oncology*.

Histogram of ln(HR)<br>CM648, OS, Nivo+Chemo, TPS≥1%<br>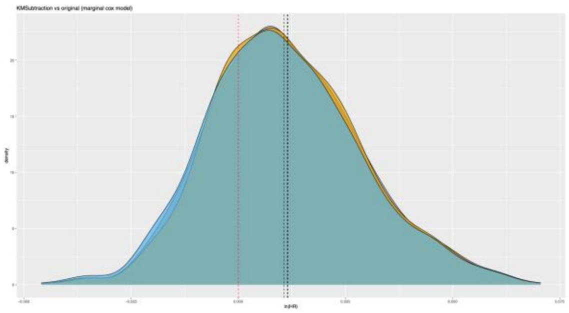	Histogram of |ln(HR)| CM648, OS, Nivo+Chemo, TPS≥1%<br>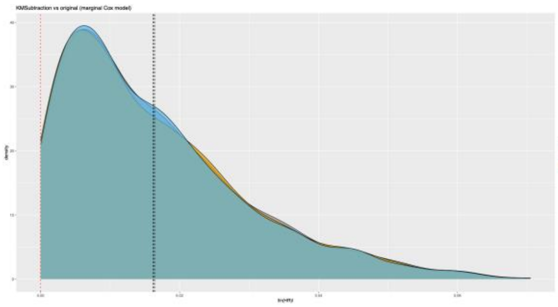	Convergence plot<br>CM648, OS, Nivo+Chemo, TPS≥1%<br>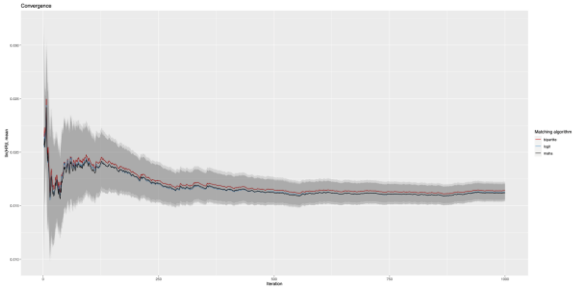
Histogram of ln(HR)<br>CM648, PFS, Chemo, TPS≥1%<br>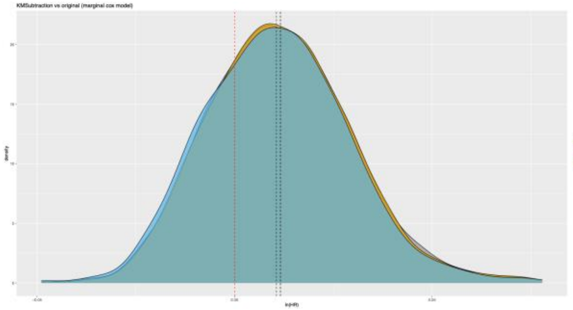	Histogram of |ln(HR)| CM648, PFS, Chemo, TPS≥1%<br>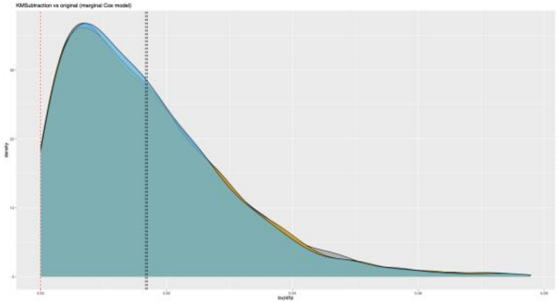	Convergence plot<br>CM648, PFS, Chemo, TPS≥1%<br>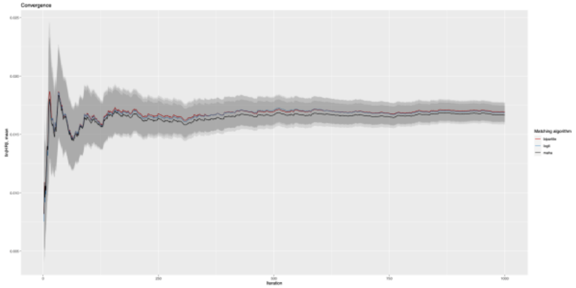
Histogram of ln(HR)<br>CM648, PFS, Nivo+Ipi, TPS≥1%<br>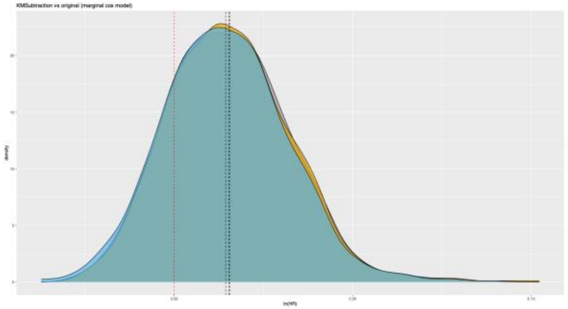	Histogram of |ln(HR)| CM648, PFS, Nivo+Ipi, TPS≥1%<br>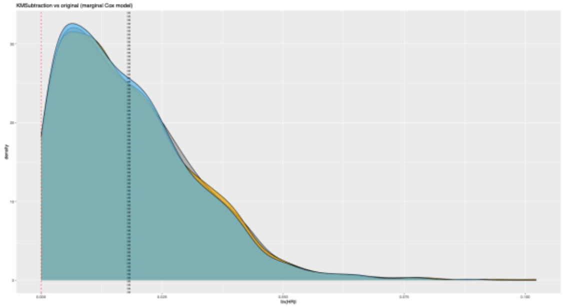	Convergence plot<br>CM648, PFS, Nivo+Ipi, TPS≥1%<br>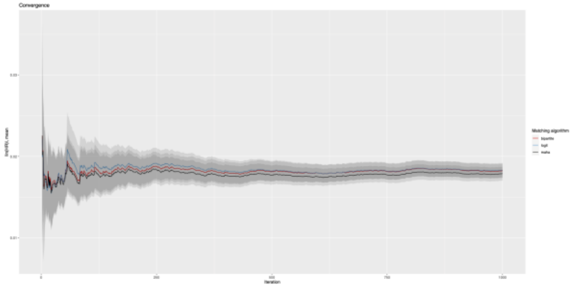

© 2022 Yap DWT et al. *JAMA Oncology*.

Histogram of ln(HR)<br>CM648, PFS, Nivo+Chemo, TPS≥1%<br>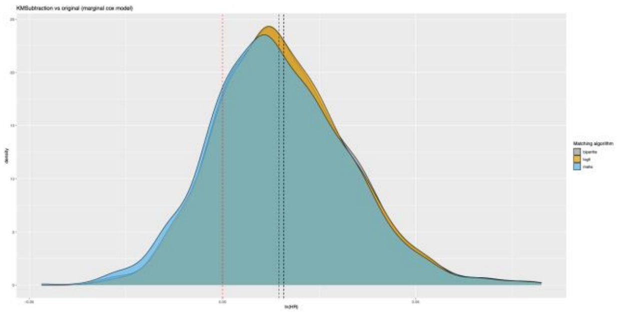	Histogram of |ln(HR)| CM648, PFS, Nivo+Chemo, TPS≥1%<br>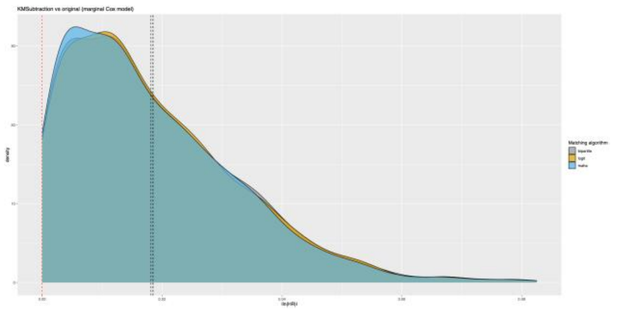	Convergence plot<br>CM648, PFS, Nivo+Chemo, TPS≥1%<br>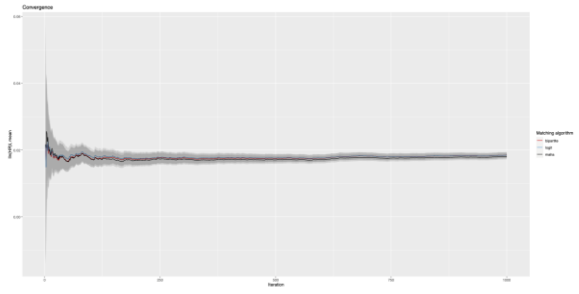
Histogram of ln(HR)<br>Escort1st, OS, Chemo, TPS≥1%<br>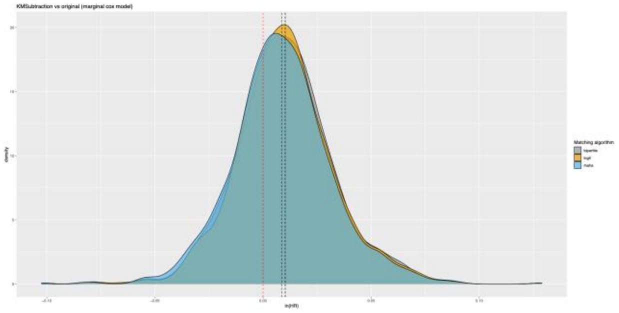	Histogram of |ln(HR)| Escort1st, OS, Chemo, TPS≥1%<br>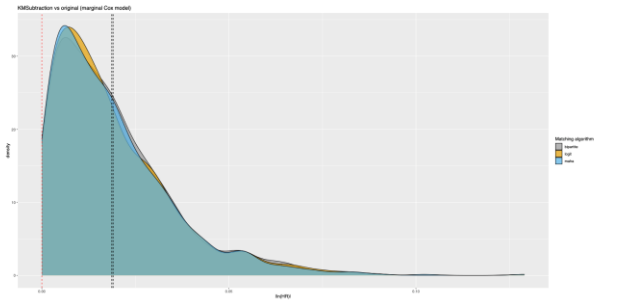	Convergence plot<br>Escort1st, OS, Chemo, TPS≥1%<br>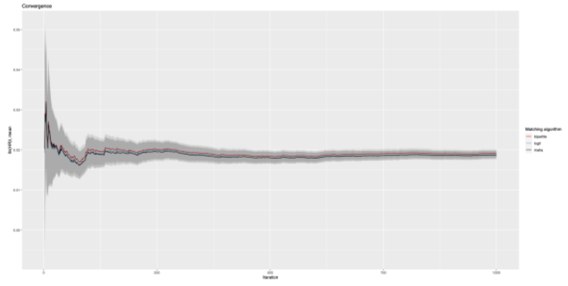
Histogram of ln(HR)<br>Escort1st, OS, Camre+Chemo, TPS≥1%<br>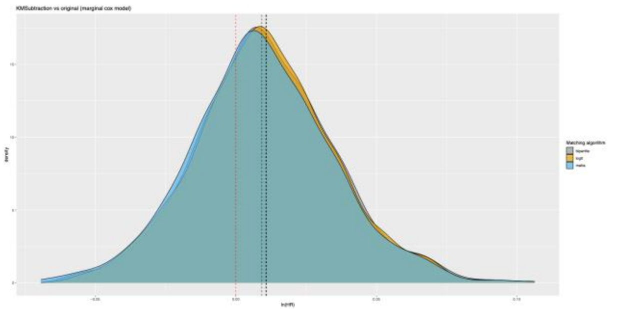	Histogram of |ln(HR)| Escort1st, OS, Camre+Chemo, TPS≥1%<br>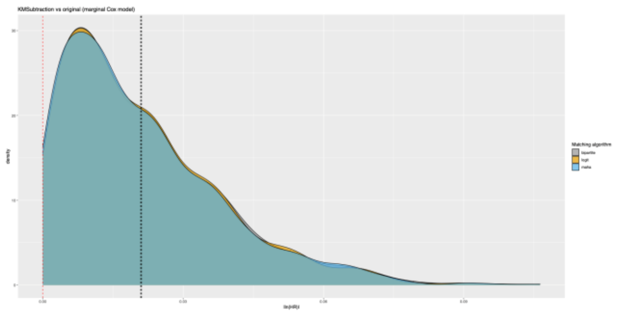	Convergence plot<br>Escort1st, OS, Camre+Chemo, TPS≥1%<br>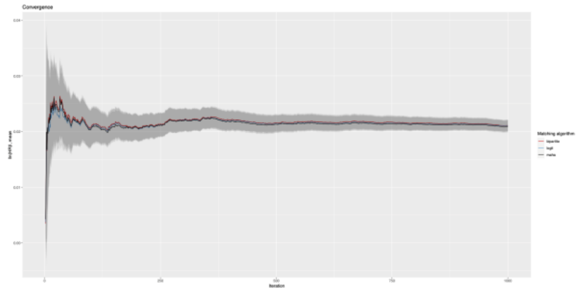

© 2022 Yap DWT et al. *JAMA Oncology*.

Histogram of ln(HR)<br>KN181, OS, Chemo, CPS ≥10%<br>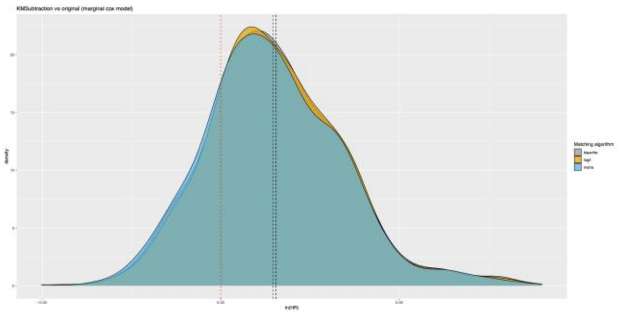	Histogram of |ln(HR)| KN181, OS, Chemo, CPS ≥10%<br>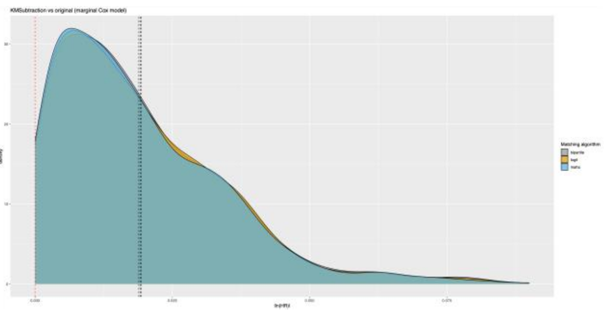	Convergence plot<br>KN181, OS, Chemo, CPS ≥10%<br>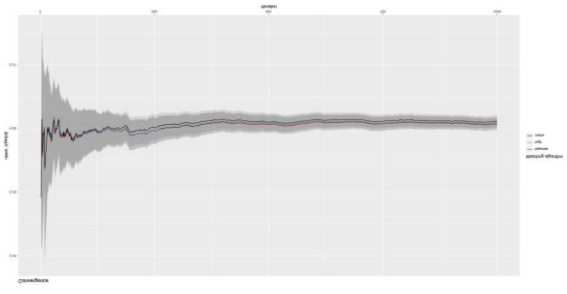
Histogram of ln(HR)<br>KN181, OS, Pembro, CPS ≥10%<br>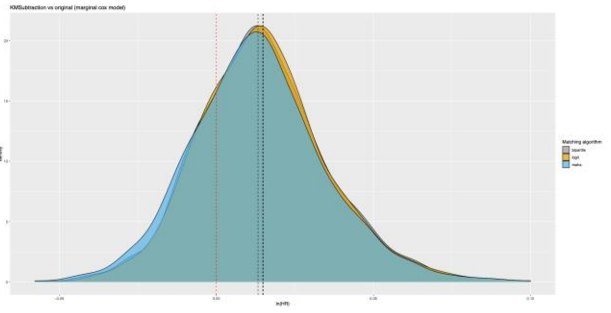	Histogram of |ln(HR)| KN181, OS, Pembro, CPS ≥10%<br>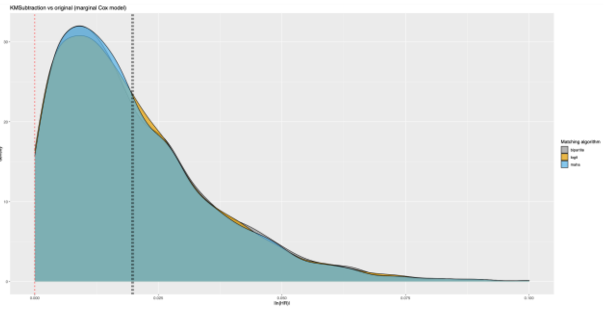	Convergence plot<br>KN181, OS, Pembro, CPS ≥10%<br>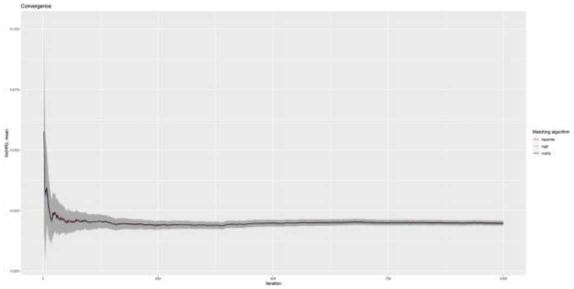

© 2022 Yap DWT et al. *JAMA Oncology*.

Histogram of ln(HR)
Attraction3, OS, Chemo, TPS≥1%

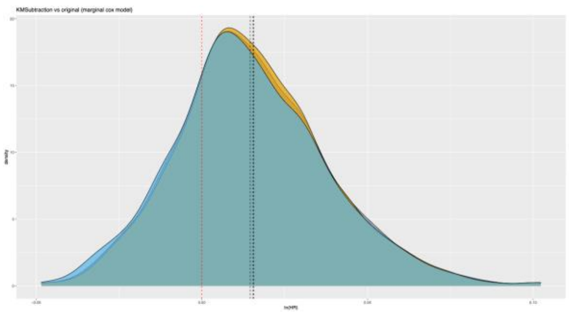

Histogram of |ln(HR)|
Attraction3, OS, Chemo, TPS≥1%

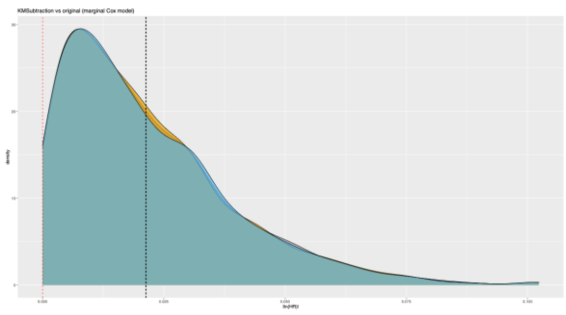

Convergence plot
Attraction3, OS, Chemo, TPS≥1%

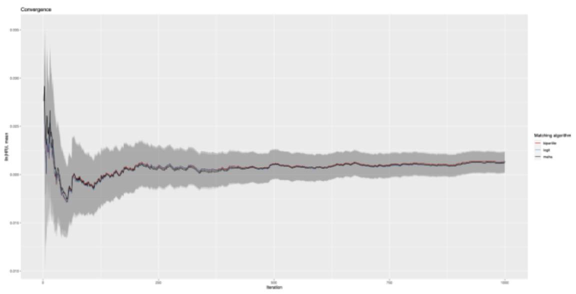

Histogram of ln(HR)
Attraction3, OS, Nivo, TPS≥1%

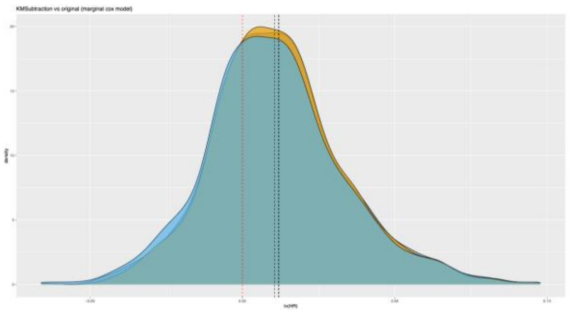

Histogram of |ln(HR)|
Attraction3, OS, Nivo, TPS≥1%

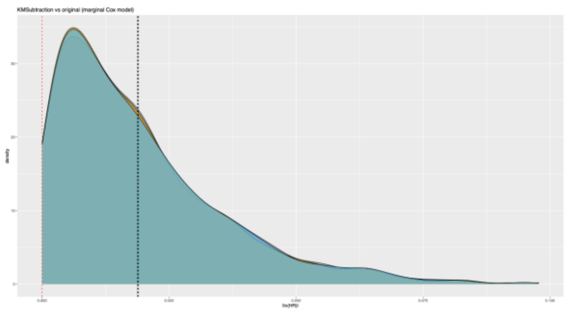

Convergence plot
Attraction3, OS, Nivo, TPS≥1%

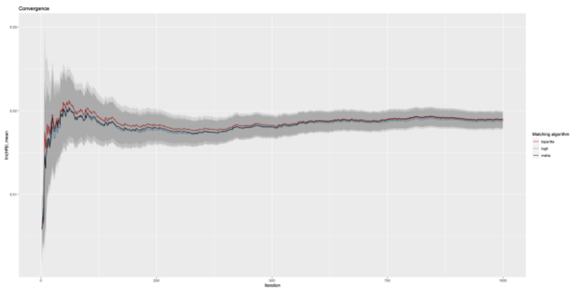

© 2022 Yap DWT et al. *JAMA Oncology*.

Histogram of ln(HR)<br>KN590, OS, Chemo, CPS ≥10%<br>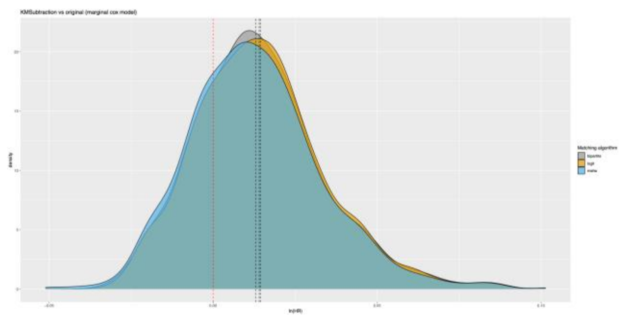	Histogram of |ln(HR)| KN590, OS, Chemo, CPS ≥10%<br>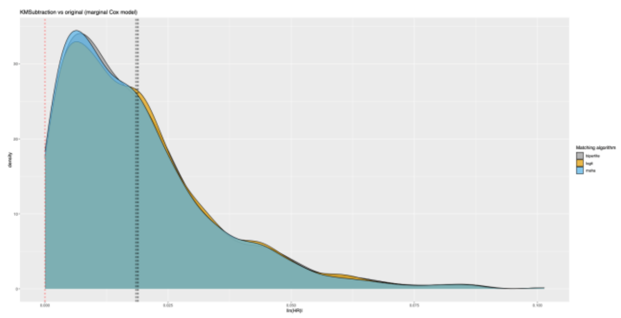	Convergence plot<br>KN590, OS, Chemo, CPS ≥10%<br>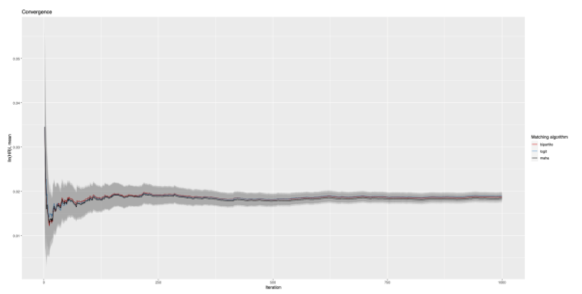
Histogram of ln(HR)<br>KN590, OS, Pembro+Chemo, CPS ≥10%<br>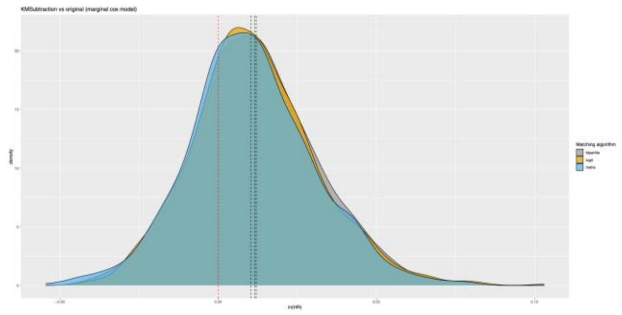	Histogram of |ln(HR)| KN590, OS, Pembro+Chemo, CPS ≥10%<br>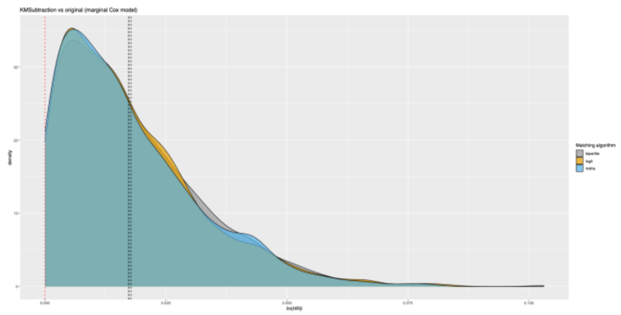	Convergence plot<br>KN590, OS, Pembro+Chemo, CPS ≥10%<br>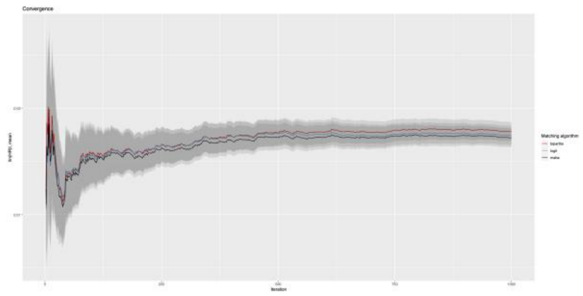

© 2022 Yap DWT et al. *JAMA Oncology*.

Histogram of ln(HR)<br>RATIONALE302, OS, Chemo, TAP ≥10%<br>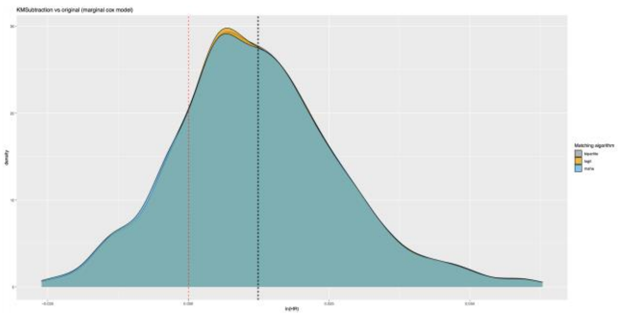	Histogram of |ln(HR)| RATIONALE302, OS, Chemo, TAP ≥10%<br>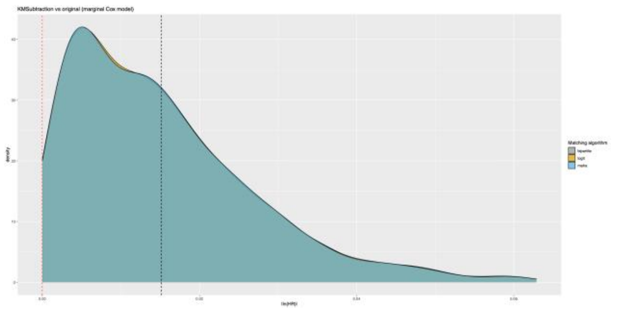	Convergence plot<br>RATIONALE302, OS, Chemo, TAP ≥10%<br>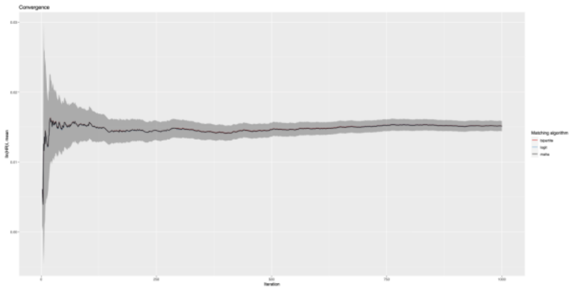
Histogram of ln(HR)<br>RATIONALE302, OS, Tisle, TAP ≥10%<br>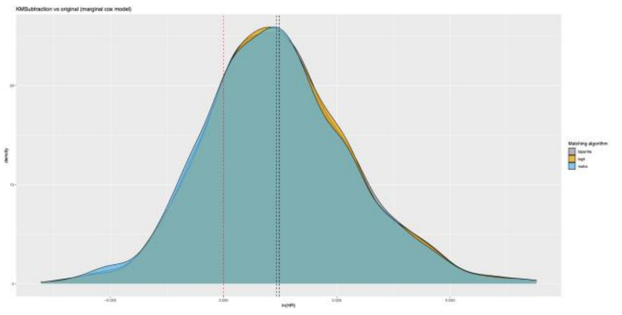	Histogram of |ln(HR)| RATIONALE302, OS, Tisle, TAP ≥10%<br>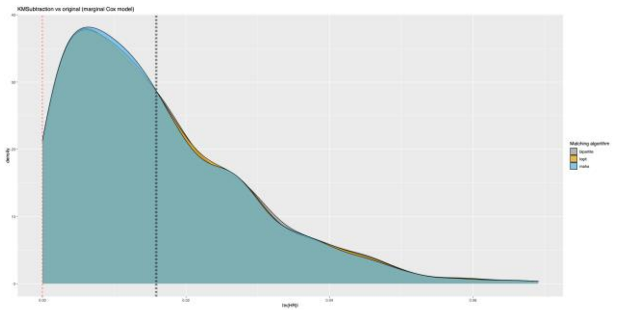	Convergence plot<br>RATIONALE302, OS, Tisle, TAP ≥10%<br>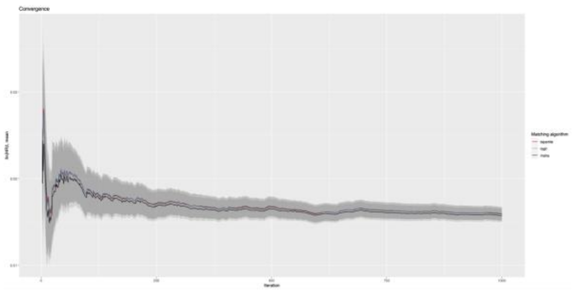

© 2022 Yap DWT et al. *JAMA Oncology*.

Histogram of ln(HR)<br>ORIENT15, OS, Chemo, CPS ≥10%	Histogram of |ln(HR)| ORIENT15, OS, Chemo, CPS ≥10%	Convergence plot<br>ORIENT15, OS, Chemo, CPS ≥10%
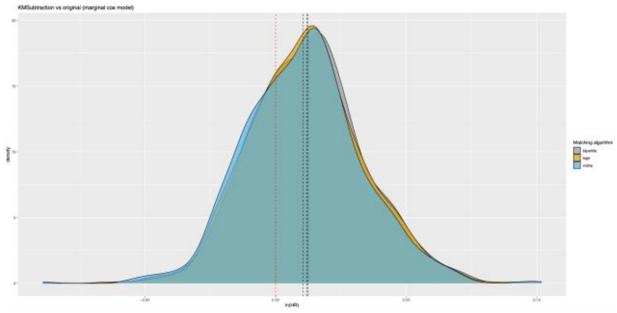	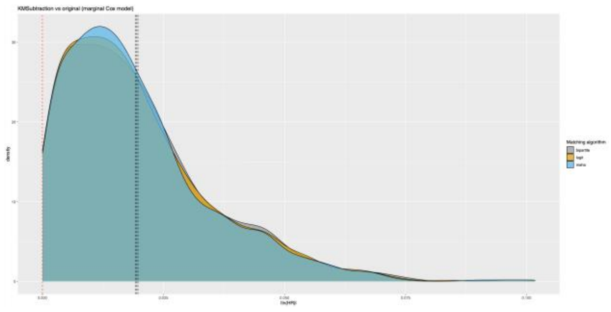	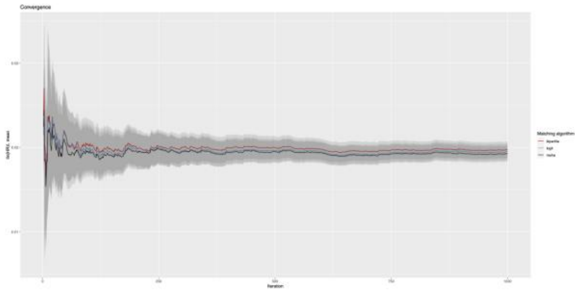
Histogram of ln(HR)<br>ORIENT15, OS, Sinti+Chemo, CPS ≥10%	Histogram of |ln(HR)| ORIENT15, OS, Sinti+Chemo, CPS ≥10%	Convergence plot<br>ORIENT15, OS, Sinti+Chemo, CPS ≥10%
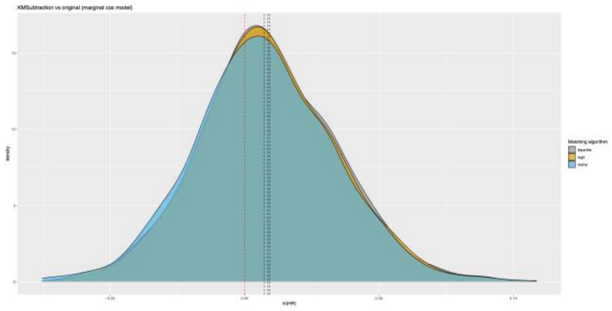	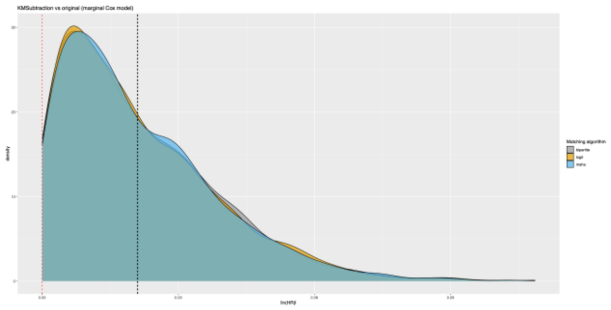	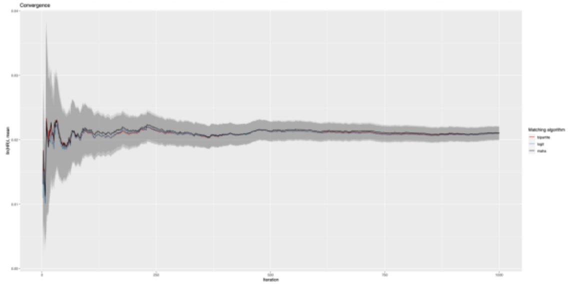

© 2022 Yap DWT et al. *JAMA Oncology*.

Histogram of ln(HR)<br>ORIENT15, PFS, Chemo, CPS ≥10%<br>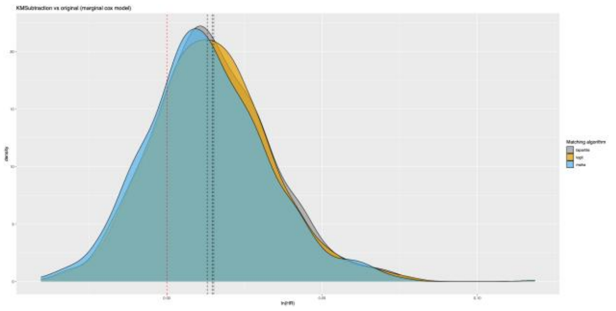	Histogram of |ln(HR)| ORIENT15, PFS, Chemo, CPS ≥10%<br>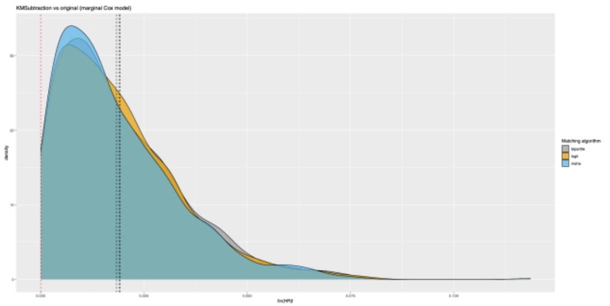	Convergence plot<br>ORIENT15, PFS, Chemo, CPS ≥10%<br>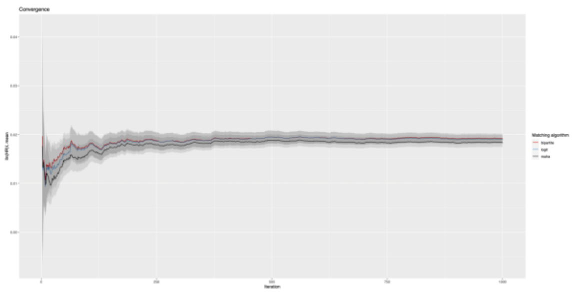
Histogram of ln(HR)<br>ORIENT15, PFS, Sinti+Chemo, CPS ≥10%<br>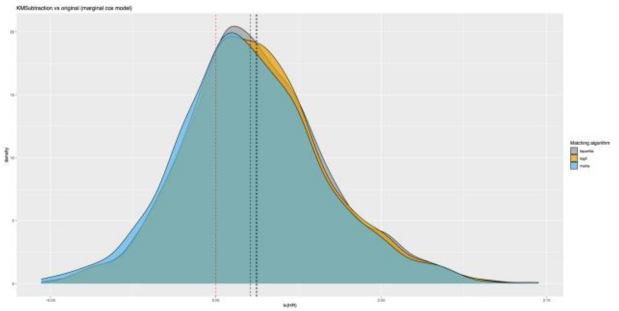	Histogram of |ln(HR)| ORIENT15, PFS, Sinti+Chemo, CPS ≥10%<br>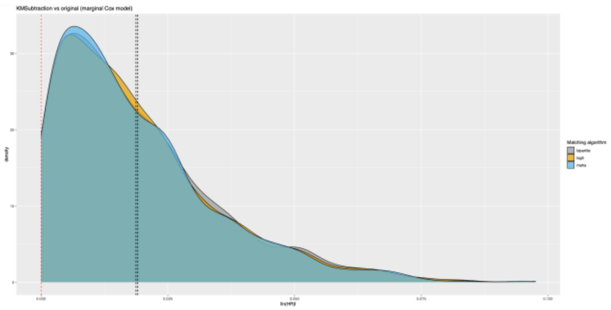	Convergence plot<br>ORIENT15, PFS, Sinti+Chemo, CPS ≥10%<br>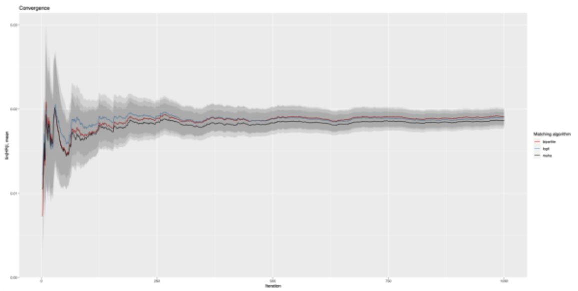

© 2022 Yap DWT et al. *JAMA Oncology*.

Histogram of ln(HR)
ESCORT, OS, Chemo, TPS ≥1%

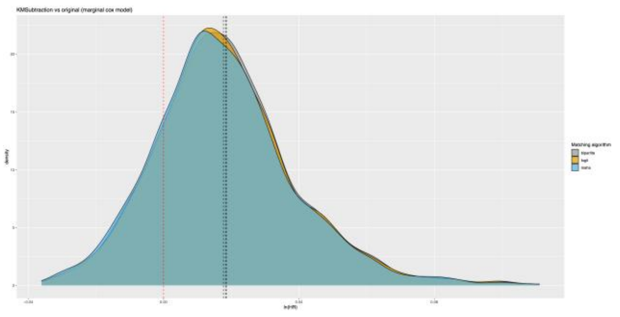

Histogram of |ln(HR)|
ESCORT, OS, Chemo, TPS ≥1%

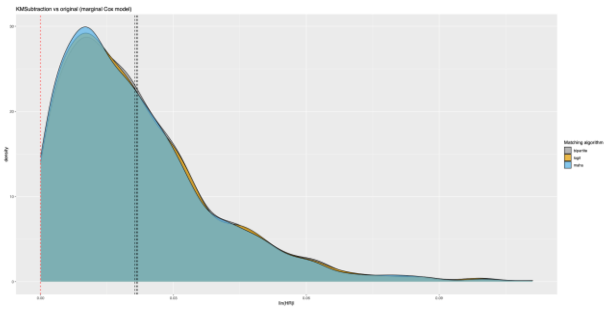

Convergence plot
ESCORT, OS, Chemo, TPS ≥1%

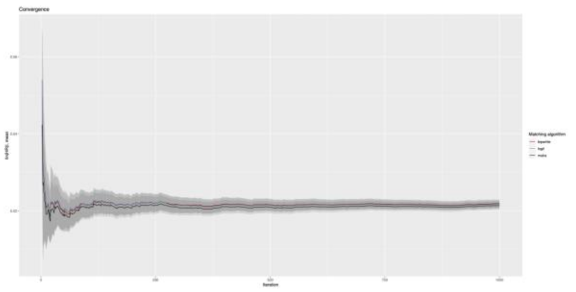

Histogram of ln(HR)
ESCORT, OS, Camre, TPS ≥1%

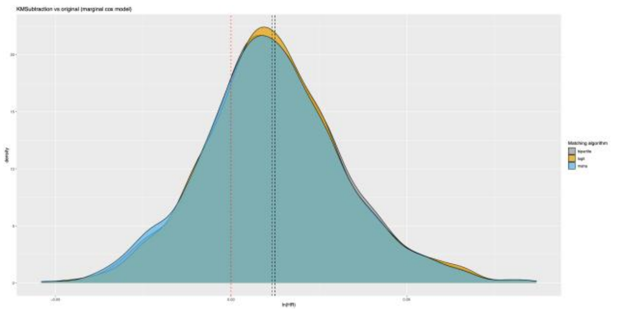

Histogram of |ln(HR)|
ESCORT, OS, Camre, TPS ≥1%

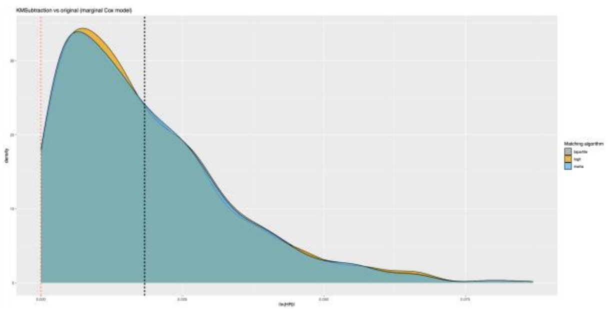

Convergence plot
ESCORT, OS, Camre, TPS ≥1%

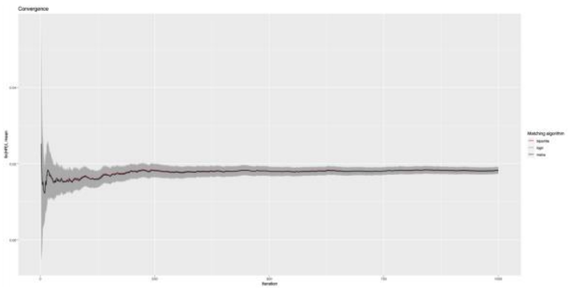

Convergence and histograms of simulations conducted for **eFigure 5** is demonstrated here. Abbreviations: Nivo, nivolumab; Ipi, ipilimumab; Camre, camrelizumab; Pembro, pembrolizumab; Sinti, sintilimab; Tisle, Tislelizumab; Chemo, chemotherapy; CPS, combined positive score; TPS, tumor proportion score; TAP, tumor area positivity; CM648, CheckMate 648; KN590, KEYNOTE-590; KN181, KEYNOTE-181.

© 2022 Yap DWT et al. *JAMA Oncology*.

eFigure 7. One-Stage Pooled Analysis of First-line and Second-line Studies

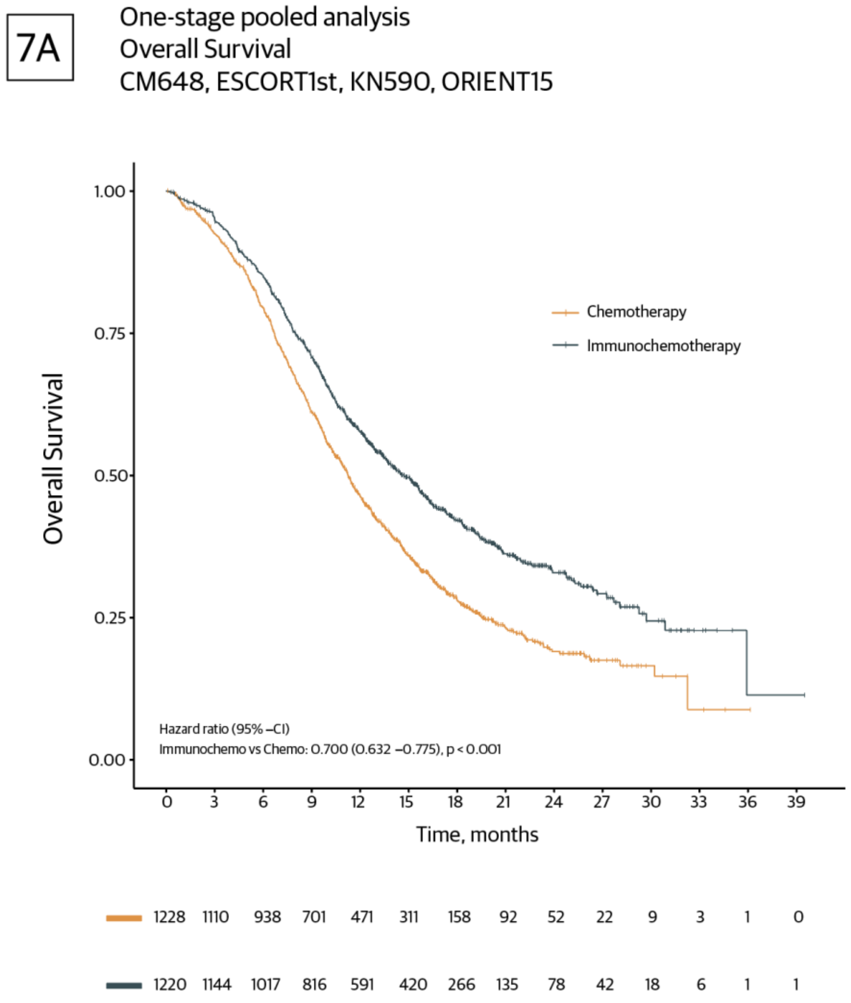

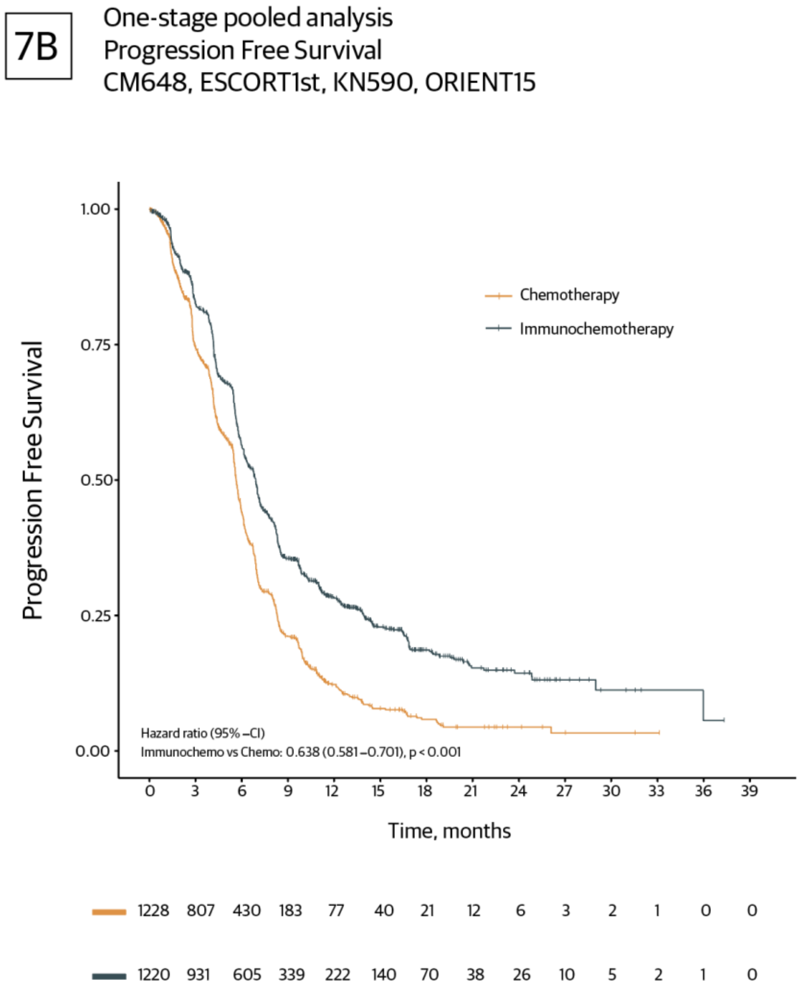

© 2022 Yap DWT et al. *JAMA Oncology*.

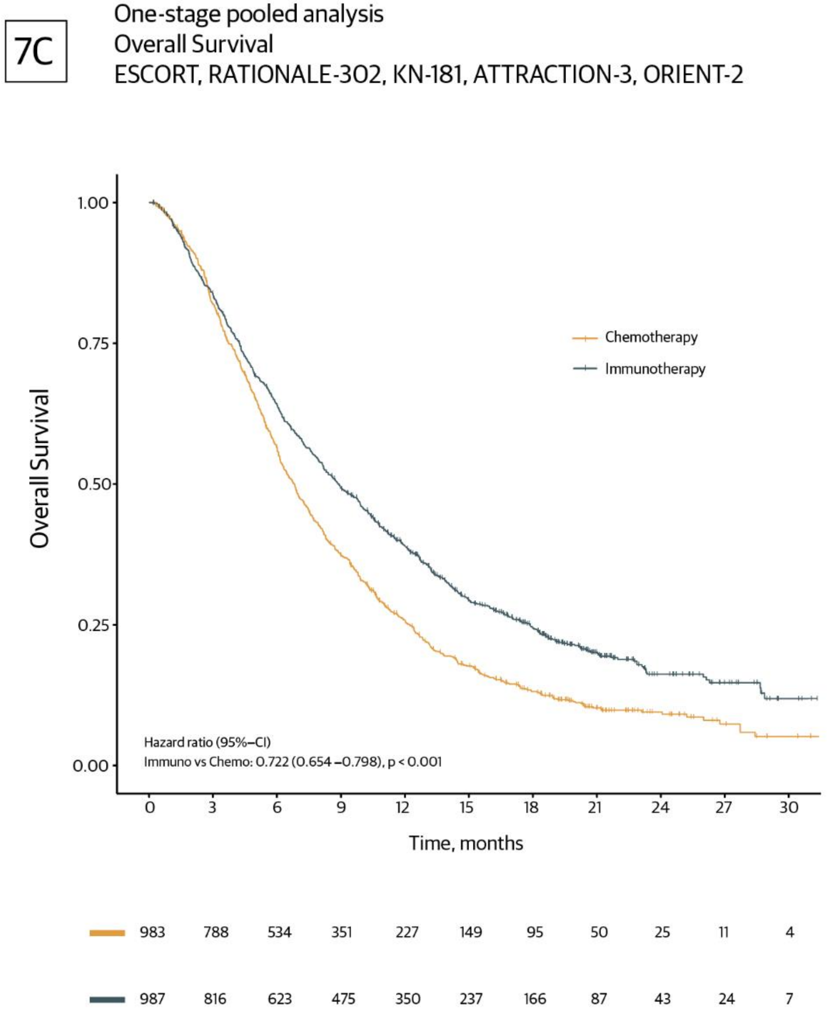

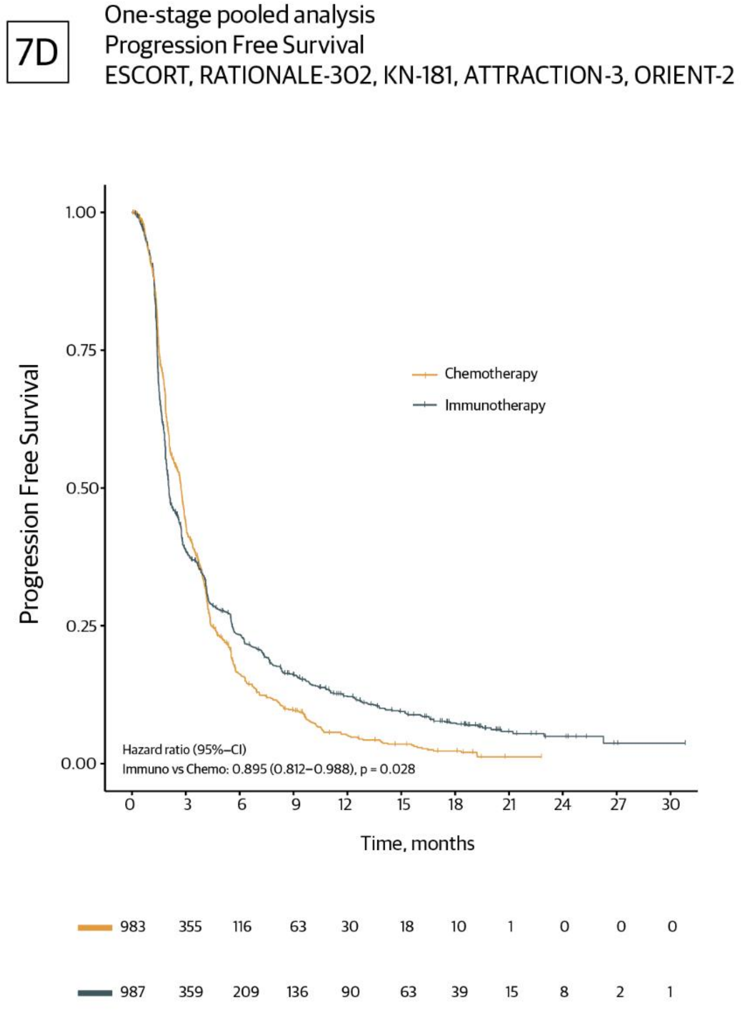

7(A) One stage meta-analyses of Overall Survival in first-line studies **(B)** One stage meta-analyses of Progression Free Survival in first-line studies **(C)** One stage meta-analyses of Overall Survival in second-line studies **(D)** One stage meta-analyses of Progression Free Survival in second-line studies

© 2022 Yap DWT et al. *JAMA Oncology*.
